# Supplementary material for: Pregnant and breastfeeding women’s prospective acceptability of two biomedical HIV prevention approaches in Sub Saharan Africa: A multisite qualitative analysis using the Theoretical Framework of Acceptability
Source: PLoS One. 2021 Nov 16;16(11):e0259779. doi: 10.1371/journal.pone.0259779 (PMC8594804; doi:10.1371/journal.pone.0259779)
Supplement: S1 File — (DOCX) [file pone.0259779.s001.docx]

**MTN-041**

Pregnant and Breastfeeding Women Focus Group Discussion (FGD) Topic Guide

**INSTRUCTIONS for the Facilitator: How to use the FGD Guide**

1. There are two levels of questions:

- Primary discussion questions: appear in **bold** text and represent all the topics you will need to cover by the end of the interview. The questions are written to ensure some consistency across FGDs but you are not required to read them verbatim. You may adapt the questions and/or ask them in a different order, depending on how the discussion develops.
- Probing topics: are indicated with a bullet. If you find that the participants provide little information in response to the primary question, these probing topics may be used to encourage further discussion. You are not required to cover every topic listed.

1. *Instructions/suggestions to facilitator are in italics and [brackets].*
2. P = pregnancy & BF = breastfeeding, which are used throughout the guide
3. The FGD guide is not meant to be used to take notes. Rather, you should use the separate notes form, where you will also insert your initials, the PTIDs of all participants, as well as the date, start and end time of the discussion.
4. Should there be need to conduct an in-depth interview (IDI) in lieu of an FGD, per approval from the management team, this FGD guide can be used for an in-depth interview (IDI) guide.

**Before starting the FGD, ensure that all participants have provided written informed consent.**

| 1. Introduction |
| --- |
| [*Facilitator should explain the following points – PLEASE DO NOT READ VERBATIM:*]   - Purpose of FGD:   - Discuss practices and taboos during pregnancy and breastfeeding   - Get your opinions about using vaginal products and oral medications during pregnancy and breastfeeding   - Understand what may influence pregnant and breastfeeding women’s use of two HIV prevention methods, a vaginal ring and oral tablet - Affirm to the participants that they are the experts and that all answers are valid: no right and wrong - Invite differing opinions - Remind participants that the discussion is confidential, so personal information won’t be shared outside of the study - Tell participants to use pseudonyms for themselves and anyone else they mention so as to preserve confidentiality - Participants should identify themselves with their pseudonym each time prior to raising a point and speak one at a time so that the audio recorder can capture everything - Remind participants to keep cellphones silent throughout discussion to avoid disruption of audio recording - *[Turn audio recorder on]* Ask participants to confirm for audio recorder that they agree to participate in FGD *[be sure to get a verbal okay from all members before continuing]* |
| 1. Health, HIV Worry, and Decision Making while Pregnant or Breastfeeding |
| *Purpose: To ask about health practices and restrictions for pregnant and breastfeeding women including HIV risk and worry.*   1. **What do women in your community do to stay healthy while pregnant?**   *Possible probing topics:*   - KEY PROBE: How about while breastfeeding? - KEY PROBE: What restrictions/taboos are there for P & BF women in your community? - What practices or restrictions are for the purpose of the mom’s health versus the baby’s health? - What changes do you make to your diet or activities? Why? |
| *Purpose: To understand who makes or influences health-related decisions for women while pregnant or breastfeeding.*   1. **Who makes decisions about what a woman should or should not do while pregnant?**   *Possible probing topics:*   - KEY PROBE: How about while breastfeeding? - From whom do women ask for advice about P & BF?   - What happens if you don’t follow that person/those people’s advice?   - When is it OK to deviate from their advice?   - From where do they get their views about P & BF? - How important are your [husband/male partner’s] opinion on what you do while P or BF?   - Does the importance of his opinion differ if you are married or not? |
| 1. **What fears or concerns do women in your community have about their pregnancies?**   *Possible probing topics:*   - *KEY PROBE*: How about fears or concerns while breastfeeding? - Where do you think these concerns/ fears come from? - With whom do you discuss these concerns? - How do you discuss these concerns with your spouse/partner? |
| 1. **While pregnant, from whom and for what do you seek care?** (e.g. medical doctor, traditional birth attendant, traditional healer, chief, religious leader, family members, or other key influencer)   *Possible probing topics:*   - KEY PROBE: How about while breastfeeding? - *KEY PROBE*: If you were given conflicting guidance, whose recommendation would you follow? Why? - What would prevent you from going to the clinic / seeing a medical doctor? |
| 1. **What kinds of products do women take, eat, rub on their body or insert to prepare the birth canal?**   *Possible probing topics:*   - *KEY PROBE*: How about while breastfeeding? - *KEY PROBE*: What medications, herbs, or products have you taken while pregnant? What for? - When is it okay or not okay to take medications, herbs or products while P or BF?   - Who provides guidance to you about these “rules”?   - If you know of conflicting “rules,” how do you decide which to follow? |
| 1. **How much do you think pregnant women are at risk of getting HIV?**   *Possible probing topics:*   - *KEY PROBE*: What about while breastfeeding? - *KEY PROBE*: How much do P or BF women worry about getting HIV while P or BF? Why/why not?   - How do these worries change from throughout pregnancy and post-delivery (during breastfeeding)? - How important is it for a woman to protect herself from HIV before, during and after pregnancy? Why so? - What HIV prevention practices do you or your partner use while you are P or BF? |
| 1. Sexual Activity and Vaginal Practices while Pregnant/Breastfeeding |
| *Purpose: To understand changes in sexual activity and vaginal practices while pregnant or breastfeeding.*   1. **What is “acceptable” in your community regarding having sex while pregnant?**   *Possible probing topics:*   - *KEY PROBE*: What is “acceptable” when you are close to delivery? - *KEY PROBE*: What is “acceptable” during breastfeeding? - *KEY PROBE:* Explain differences in sex practices from before, close to delivery, after delivery and while breastfeeding.   - How soon after delivery do you resume sexual activity with your husband/partner?   - What influences your sexual activity while pregnant or breastfeeding?   - What do you do to prepare for sex while pregnant or breastfeeding? - How acceptable is it for men to have other sex partners while their partners are P or BF?   - What about after delivery, while you are breastfeeding? - After pregnancy, when do you start using contraception, if at all? Why so? |
| 1. **How do women in your community prepare your vagina/birth canal for delivery? Why?**   *Possible probing topics:*   - What practices do women do to clean their vaginas while pregnant or breastfeeding? - What products are used every day versus on occasion?   - How are these used (inserted into vagina, swallowed, etc.) - Are women supposed to take or use different products for different times throughout your pregnancy or during breastfeeding? Please explain. |
| 1. Ring and PrEP Discussion |
| *Purpose: 1.) To understand what women know and think about using oral PrEP or the vaginal ring for HIV prevention while pregnant and, 2.) To hear suggestions about how to get pregnant or breastfeeding women to use these products.*  **As explained before, we are interested in getting your opinion about two different products that women can use for HIV prevention, daily oral PrEP tablets and the monthly vaginal ring.** [*Give information on products and/or show or provide samples of products to the group*]   1. **Let’s start by talking about daily oral PrEP tablets. What is your first thought when you think about taking oral PrEP to prevent HIV while pregnant?**   *Possible probing topics:*   - *KEY PROBE*: What about while breastfeeding? - *KEY PROBE*: Would oral PrEP be something you’d be interested in using while P or BF? Why or why not? - *KEY PROBE*: What worries do you have about oral PrEP and why? (about your health, your baby’s health, your partner’s health, stigma of taking a daily tablet/ARV, etc.) \   - - What about the tablet would make it hard to take? e.g. its size, taste, timing of use - What would facilitate taking oral PrEP for P and BF women? - What period of time during P or BF would it be most feasible to take oral PrEP? - How would the tablet interfere with your daily life? (sexual activity, contraceptive use, etc.) |
| **Depending on the culture, it may be permitted or taboo to take bitter medicine while pregnant. How would this interfere with women in your community’s ability to take oral PrEP while pregnant?**  *Possible probing topics:*   - *KEY PROBE*: What about while breastfeeding? - What other local taboos or practices would make taking oral PrEP while pregnant difficult? |
| 1. **Now let’s talk about the vaginal ring. What is your first thought when you think about using a vaginal ring to prevent HIV while pregnant?**   *Possible probing topics:*   - *KEY PROBE*: What about while breastfeeding? - *KEY PROBE*: Would the vaginal ring be something you’d be interested in using during P or BF? Why/why not? - *KEY PROBE*: What worries do you have about the ring and why? (about your health, your baby’s health, fear of feeling it or partner feeling it during sex, other concerns, etc.)   - What about the ring would make it hard to use? e.g. its size, timing, comfort/discomfort, etc. - What would facilitate using the vaginal ring for P and BF women? - What period of time during P or BF would it be most feasible to use the vaginal ring? - How would the ring interfere with your daily life? (sexual activity, contraceptive use, etc.) |
| 1. **Depending on the culture, it may be permitted or taboo to insert things in the vagina while pregnant. How would this interfere with women in your community’s ability to use the vaginal ring while pregnant?**   *Possible probing topics:*   - *KEY PROBE*: What about while breastfeeding? - What other local taboos or practices would make using the ring while pregnant difficult? - How would using these products clash with your cultural practices and beliefs around P & BF? - What would help you overcome these clashes with your cultural beliefs or practices? |
| 1. **Would pregnant women in the community want to use products like these? Why or why not?**   *Possible probing topics:*   - *KEY PROBE*: What about breastfeeding women? - *KEY PROBE*: What preferences would women have for oral PrEP versus the vaginal ring? - *KEY PROBE*: How interested in these products would women be if they are about as effective as condoms?   - What is the minimum level of protection that these products should provide? - How would using these products clash with your cultural practices and beliefs around P & BF?   - What would help you overcome these clashes with your cultural beliefs or practices? - Do you think P & BF women would be able to use these products successfully? Please explain. |
| 1. **Besides your doctor(s), who should be involved in deciding whether a woman uses one of these products while pregnant or breastfeeding?**   *Possible probing topics:*   - Should your male partners be involved? How? - Should your mothers/mothers-in-law be involved? How? - Who else (traditional birth attendants, traditional healer, religious leaders… )? |
| 1. **What do you think is the most important factor that would motivate a pregnant or breastfeeding woman to use the ring or the tablets for HIV prevention?**   *Possible probing topics:*   - *KEY PROBE*: What could be done to help facilitate or encourage women to use these products? - How do you suggest we remove the barriers she may experience to using these products? |
| 1. **What suggestions do you have on how we can recruit pregnant and breastfeeding women for a future study in which they will use these products?**   *Possible probing topics:*   - *KEY PROBE*: What do you think about the information we shared about the two products earlier? [*Show product information again if necessary]*   - What can we change or add so people who have never heard of oral PrEP or the vaginal ring for HIV prevention understand the products? - What kind of messages would appeal to P and BF women in your community?   - How could we gain their trust so women will be willing to join these studies? |
| Wrap Up |
| 1. **Thank you for taking the time to share your opinions with us today. We truly appreciate your willingness to participate and discuss your thoughts and ideas with us. We’ve now reached the end of our discussion. Do you have any additional comments about your pregnancy and breastfeeding experiences?** |
| 1. **Do you have any additional comments about oral PrEP or the vaginal ring?** |
