# Supplementary material for: Pregnant and breastfeeding women’s prospective acceptability of two biomedical HIV prevention approaches in Sub Saharan Africa: A multisite qualitative analysis using the Theoretical Framework of Acceptability
Source: PLoS One. 2021 Nov 16;16(11):e0259779. doi: 10.1371/journal.pone.0259779 (PMC8594804; doi:10.1371/journal.pone.0259779)
Supplement: S2 File — (DOC) [file pone.0259779.s002.doc]

**Code reports**

**Acceptability code report**

**CODES:** ACCEPTABILITY (T) **+** EFFICACY (T) + SAFETY & SIDE EFFECTS (T) + USE/TIMING (T) + PREFERENCE (T) + WILLINGNESS TO USE/RECOMMEND USE (T)

**TRANSCRIPTS:** ALL P&BF WOMEN FGDs

**MALAWI:**

**FGD Number:** FGD_41-F81

**Site:** Malawi

**Excerpt Range:** 29587-30276

I: What kind of worries can the women have in taking these tablets daily and why can they have those worries? [Baby crying irritably though softly].

Lucy: Yes worries are inevitable. Taking these tablets to protect yourself from HIV while you are pregnant can be worrisome. This can be so, because you don't know how the drugs will work in your body while you are pregnant. They may come with so much strength that may lead to fatigue, or can even destroy the baby you are expecting. So, yes it is good that the drugs will protect from HIV, but they may bring some undesirable side effects while you are pregnant; as it is said that when one is pregnant, she should not be taking drugs.

**FGD Number:** FGD_41-F81

**Site:** Malawi

**Excerpt Range:** 31453-32868

I: When do you think is the right time for the woman to take the tablets…when she is pregnant or breastfeeding, what is the time that she can be taking the tablets?

Alice: When she is expectant.

I: Yes, the woman is pregnant and is breastfeeding, but the thing is when do you think is the time, say morning, afternoon, evening or bedtime. Which is the right time for her to take the tablets?

Alice: At bedtime.

I: At bedtime. Thank you. Ethel, you wanted to say something?

Ethel: When she is about to go to bed, she should take the tablets because the time she is sleeping, the tablets work better in the body.

I: Are there any different thoughts from the ones that have been given?

Esther: Just to add on the time you go to bed. You cannot be taking the tablets daily. It may happen that the husband is hardly home because he is involved in field work mostly. Is it okay to continue taking them? When the husband is out in the field?

I: From what we saw in the video, it said we have to take the tablet each and every day, not so? It does not matter if the husband is gone for field work, eh? Just like someone said that it may happen that a woman can get pregnant and not stay together with the man but they can be meeting say once a month or even once in two months… She still has to take the tablets daily because if you don't take the tablets daily, your protection levels will be compromised...

**FGD Number:** FGD_41-F81

**Site:** Malawi

**Excerpt Range:** 34475-35714

What do you think are the things that can prohibit from taking these drugs, these tablets, from practises and cultural beliefs that are there in our communities? What cultural beliefs can prohibit a woman from taking the tablets that can protect them from HIV the time they are pregnant?

Favour: What can prohibit women from taking these tablets is that, in our communities there a people who say a lot of things, they may say a lot of discouraging things about the PrEP tablets. But as an individual, you just have to see how things are going now in this world and listen to what the hospital personnel are saying and also how our health is these days, so that you should use these tablets.

I: What kinds of discouraging things?

Favour: It may happen that people can say lots of things about these tablets that it can make you think of not using them.

I: I want you to tell me the exact discouraging words that can be said.

Favour: They may say the tablets are bad and may destroy you inside.

I: Alright, Is there any addition?

Ethel: There are some who believe in religion. They may go collect the tablets then just dump them home and they say you should just be praying and the Lord is going to deal with the infection.

**FGD Number:** FGD_41-F81

**Site:** Malawi

**Excerpt Range:** 38606-38943

Favour: And also this vaginal ring I feel is a good method because when you put it once, it means it is there for the whole month and you will be using that very same ring while with tablets you may forget due to being occupied, while with the ring, once you insert it, you3 are done and you just have to remember the date to change it.

**FGD Number:** FGD_41-F81

**Site:** Malawi

**Excerpt Range:** 40107-40761

Inserting the ring in the vagina, in our communities, how acceptable or not acceptable can it be? Or I should say how can people accept it or how acceptable can it be? [Babies’ voices heard playing in the background]

Ethel: It all depends on what the family agrees on, because when you hear about things like these, you may explain it to your husband, and according to how you have agreed in your family, you can then use this thing.

Kheliwe: How about when someone is not married?

Ethel: It all depends on how she feels about it. The way she has heard and understood it. She’s free to decide on what she wants to do depending on her understanding.

**FGD Number:** FGD_41-F81

**Site:** Malawi

**Excerpt Range:** 40796-41211

Let us not just concentrate on what we think but also on how our different communities can take it? How do we think people can take it?

Favour: I feel people in the communities can accept it nicely, because to someone who is HIV negative, she is supposed to accept it, because if she takes this method, wherever she goes, she knows she is protected. So, I feel people in our communities will welcome this method.

**FGD Number:** FGD_41-F81

**Site:** Malawi

**Excerpt Range:** 43063-43787

I: Is there any different thought from the two that have been given?... Thank you. So, women in our communities, would they want to use these products? What can make them not want to use them? Taking from what we have shared on the ring and oral tablets and we look closely in our communities, what do we think, would women want to use them?

Patricia: May be some would not want to use them as they would not want to take the daily tablets. They would say it is just the same as taking ARVs. They may not differentiate between the two

I: Thank you very much. Nabanda there!...[Laughs]

Nabanda: Some people might want to use the ring rather than taking tablets daily.

I: It all depends on what someone likes eh?

R: Yes.

**FGD Number:** FGD_41-F81

**Site:** Malawi

**Excerpt Range:** 45894-46543

I: Thank you very much. Which methods do you think we can use in removing barriers that can prevent women from using these products? After being taught about these products, there are some things that can prevent people from accepting them from, what methods can we use? What can we do to remove these barriers?

Favour: it is hard for people to accept something if they only hear about it for the first time. When something is new, it might be impossible for people to accept that. So likewise, for these methods that we have just learnt today, there is need to be discussing on them frequently so that people should accept them little by little.

**FGD Number:** FGD_41-F81

**Site:** Malawi

**Excerpt Range:** 48241-48343

Kheliwe: I just want to ask, are the tablets only for women and not for men? Men cannot be protected?

**FGD Number:** FGD_41-F81

**Site:** Malawi

**Excerpt Range:** 49017-49459

Ethel: When you insert the ring, is it going to protect the woman only?

I: Yes. What happens is that when you use the ring, the medication that is in the ring is released into your body and then act as soldiers to fight against HIV if it enters in your body.

Ethel: And not help the man?

I: No. [Laughs]

Ethel: Some say when you have received an injection, it also works on the man?

I: Which injection?

Ethel: The one for contraception.

**FGD Number:** FGD_41-F81

**Site:** Malawi

**Excerpt Range:** 49596-50183

Lucy: I have this concern. Is it not possible to make the tablets work for a longer time? If they can combine the strength of the tablets into one so that it can be used for say, a month.

I: Meaning a tablet should work for a month?

Lucy: Because taking the tablets daily is hectic and also you may forget easily.

Favour: And taking of the tablets daily will just be like you are taking ARVs and people will not see the difference. When you start taking them, that is it, you continue taking them maybe till the time HIV is eradicated. That is when we will stop taking the tablets.

**FGD Number:** FGD_41-F84

**Site:** Malawi

**Excerpt Range:** 59877-60866

I: So let us first discuss about this pill…what are our first thoughts about this pill about what we have seen and heard in the video…what are our first thoughts… While we are thinking about this pill being taken by a pregnant woman for HIV prevention? ... what are we saying?

[Triza]: From what we have seen in the video, they are saying it needs to be taken daily…it is the same as contraceptive pills…I think so…and I want to ask, is it not the same if you miss one day can’t you get the HIV infection?

I: Have we all heard [Triza’s] question?

All: Yes.

I: In the video what did they say about the correct way of taking the pill?

Unidentified participant: … daily.

I: Yeah, it needs to be taken daily without missing…so whenever you miss, there could be a risk of you contracting the virus, just as you have explained in relation to contraception pills…if you miss one day, you can conceive…it’s the same with this pill… one’s immunity can drop when they have not taken the drug.

**FGD Number:** FGD_41-F84

**Site:** Malawi

**Excerpt Range:** 60921-62442

what are our thoughts after seeing the pill?...is it something that if it can be certified we can be able to take it during pregnancy as a way of protecting ourselves from HIV?...if we can take it why would we take it and if not why would we not take it? ...those are some of the thoughts we can share here?

[Triza]: If they have come up with a drug that would protect us from contracting HIV, I think it would be acceptable for me to take it for me to protect myself from HIV, because if they have brought the drugs, they must have tested it and got assurance that it cannot harm a person…not taking the drug would be because of fear, some may think the drug might be harmful… to say the truth those who are pregnant might fear taking the drug thinking it might induce an abortion…you know people talk a lot of things.

I: Yeah, those are the things we would like to hear.

[Triza]: They may say, see medical people…they may say it is government’s plan to kill people…people talk a lot.

I: As a way to reduce population?

[Triza]: Yeah…we are many in this country and they are just introducing their drugs to kill us…so there are many views, but as it is, the government cannot allow people to just die because ‘dziko ndi anthu’ [ local expression meaning ‘a nation is recognized as a nation because it has people’]…so the government cannot say, ‘let the people die’

I: So what is your view?

[Triza]: For me I would take the drug.

I: So you would take…for you to be protected?

[Triza]: Yeah for me to be protected.

**FGD Number:** FGD_41-F84

**Site:** Malawi

**Excerpt Range:** 62523-63306

how about someone who is breastfeeding?...what are your views?...the mother is breastfeeding a small baby like this one [pointing at one of the babies in the room]…and those other babies we have left outside…is there any concern for a mother that, because am breastfeeding the drug might cause something or it is just fine?

[Triza]: Maybe the concerns could be there since people are different, because that will be entering the body, so as it enters the body something might come up…as you know Satan is clever…maybe if a mother starts taking this and breastfeeding the baby, the baby might develop some sores or do other things, and one might think ‘maybe it’s the what I inserted or the pill that I take’.

I: Is the one causing it?

[Triza]: Yea, since this is new…so …[giggles]

**FGD Number:** FGD_41-F84

**Site:** Malawi

**Excerpt Range:** 63308-63810

I: So, the way we have seen the tablet, what is it about the pill that would make the women not to take it daily?

[Triza]: The problem with the pill is being forgetful… if we forget to take the contraceptive pills…we do remember late, waking up past something…to take the pill…[laughing]…so the pill, I think for those that can remember they can take it but not for those that are forgetful like me…I can’t try it…[giggling]…it is better I opt for the ring because I will just stay with it…[laughing]

**FGD Number:** FGD_41-F84

**Site:** Malawi

**Excerpt Range:** 63830-64318

What do you think would help a woman to be able to take the pill…a pregnant woman and a breastfeeding mother…what would facilitate their taking of the pill…what would that be?

[Alinafe]: For me I would take it after giving birth and maybe my husband is going out his way because it is not possible for me to have sex with him soon after giving birth…so he would be going out his ways while am taking the pill, by the time we will resume sex I will have already started protecting myself.

**FGD Number:** FGD_41-F84

**Site:** Malawi

**Excerpt Range:** 64320-65312

I: So our friends have shared with us what would encourage them to take this pill…yes Monica, do you have different views from what our friends have shared?

[Monica]: [sighing] …no everything is okay.

I: What would encourage you to take the drug every day?

[Monica]: For me I would opt for the ring.

I: You would opt for the ring…would you share with us why you have chosen the ring and not the pill…that is your right…but we just want to know what would stop you from taking the pill every day?

[Monica]: Pills are bitter.

I: Oh bitterness…so that would make you choose the ring?

[Monica]: Yeah… it thought the ring remains inserted for a month?

I: Yes, for a month…so what is the main reason you would choose the ring?

[Monica]: The ring has pleased me and not the pills.

I: How has it pleased you?...please clarify on that.

[Monica]: It has pleased me that the ring can remain for a month.

I: A month still inserted?

[Monica]: Unlike taking the pill daily…I can easily forget.

**FGD Number:** FGD_41-F84

**Site:** Malawi

**Excerpt Range:** 65399-66728

in your views what time is the best for a woman to take the pill…the one who is pregnant, what is that best time for her to take the pill so she doesn’t forget?

[Monica]: Anytime, when the woman is pregnant or breastfeeding.

I: Yeah there we have heard…but taking into account how a day goes like from morning, afternoon and evening, when is the best time for the woman to take the pill?

[Monica]: In the evening.

I: Why have you chosen in the evening?

[Monica]: When going to bed…or does it take long to dissolve…[laughing]…

I: We just wanted to know why you have chosen the evening?

[Monica]: When the man is back from work, he might remind me…

I: Oh so for you to have someone to remind you…who else is in agreement with the evening time and your reason for choosing the evening?

[Maggie]: The reason for choosing to take the pill in the evening is that, it could be that the pill has a smell, so some people do not like drugs with smell so they will think it is better to take the drug in the evening and the smell can go away once one gets into sleep because they might not think about the smell, unlike taking it in the morning then the smell will be in the head.

[Monica]: The drug might also make you hungry, so it is better to take it in the evening when your stomach is full…and the drug will just be working.

**FGD Number:** FGD_41-F84

**Site:** Malawi

**Excerpt Range:** 66878-67277

Now how can the daily taking of the pill disrupt your day to day life?

[Debora]: I think just taking the drug only cannot have any problem.

I: Okay, just taking the drug cannot have any problem… that is your view?

[Debora]: Yeah, unless after taking the drug one starts experiencing other things that you were not experiencing like feeling dizzy after taking the drug…I think such things, may be…

**FGD Number:** FGD_41-F84

**Site:** Malawi

**Excerpt Range:** 69882-70901

others have already said their views that they would prefer the ring and they have also explained their reasons… for us after seeing the ring, what are our thoughts about the ring as a way a woman can use by inserting to protect themselves from HIV?

[Debora]: I would prefer the ring for the following reasons; with the ring on its own, once inserted one can move about with it, you can have sex with the husband just as you always do without any problem so I think the ring is much better because it is done once in a whole month…so I would prefer the ring because I can’t forget, I can just be staying until the month ends.

I: Alright…so in your view we can say that between the two you have opted for the ring and not the other method.

[Memory]: I also would prefer the ring…why for the pill, you have said that once one forgets to take it and you have sex, it means you are at risk, while the ring, once inserted it is until you remove it…[giggling]…no one will see it and you can’t forget…so I think it is good.

**FGD Number:** FGD_41-F84

**Site:** Malawi

**Excerpt Range:** 72534-73523

do you think there is anything that would encourage a woman to use this ring?...what would encourage the woman to use the ring consistently?

[Memory]: For a woman to use the ring consistently, she will prevent a lot, and she might not have many worries because she has this… whether the man goes out, she might not have worries as we have…because she has protected herself.

I: Okay, so the feeling that something inside me is protecting me, that would make the woman to use the ring accordingly?

[Triza]: It’s not that one won’t be complaining that the husband is going out…that is why we are having these things…our understanding is different…and these things are coming to protect us…it might happen that women might start being rude to the husband because they are using protection…saying no matter what I do I can’t contract the infection…but this is just to protect us so we can have long life…and be protected…it should not change us as women…our marriages should remain the same.

**FGD Number:** FGD_41-F84

**Site:** Malawi

**Excerpt Range:** 73571-75836

basing on tradition how acceptable or not acceptable is it to insert a thing in the vagina during pregnancy or breastfeeding and move about with it…based on tradition from the communities we are coming from…from what we hear or see…what we were told or what our friends talk about…do you think it is acceptable and if yes, why?

[Debora]: I would say like this, these days are different from the past days…..these days as compared to how people used to live in the past things are not the same, so for someone to prohibit that can’t work…..in the communities people have different beliefs and also those that went to school are different from those that grew up in the villages, so if one grew up in village and someone who went to school you will be different.

I: In the village there are also others who went to school.

[Debora]: Yeah.

[all participants laugh]

[Debora]: For the school in the village I mean their behavior…in the village we are too much settled on tradition…for instance this cancer disease, someone would say ‘eeeh did you see that one, she died because she was taking such a thing’…so maybe there could be some prohibitions by parents that, ‘if you are my child don’t take this because the disease is dangerous and it killed such and such a person…but this is contributing good health to you, if the medical people have provided us let’s take them to help us in that area.

[Triza]: There it can’t be because they took something… but the main issue is…for example it has been said about cancer…even contraceptives are said to cause cancer… And many are going to witchdoctors.

I: To get contraceptives?

[Triza]: Yes to get contraceptives, they tell you to take some drugs…and people bargain there and they would say I want three years…as you know those people [with doctors] do have their own spirits…so people may say 10 years and they give the drugs or we out incisions on you and you will stay 10 years, or 5 years or three years…all because the modern contraceptives are causing cancer…the uterus is developing cancer in many women…so I say that, then these things are going to kill many of us…you know there is a lot of talking in the communities…[giggling]

I: Yeah, people will never stop talking.

[Triza]: Yeah they can’t stop.

**FGD Number:** FGD_41-F84

**Site:** Malawi

**Excerpt Range:** 75838-76581

I: What do you think should be done to deal with the beliefs on the ring or the pill?

[Debora]: I think educating people, and explaining to them that there is this and that issue, what is your view or how do you see these … I think it is a good way to teach someone.

I: Yeah teaching a person… alright.

[Triza]: While we are teaching them at this works like this… there are some who cannot accept it even after educating them…it is the same as contraceptives, others do not use them, it is their choice…but if you can still educate the people and let the decision be theirs whether to go and access it or not but you should still educate them, just for the future, maybe they will regret that if I had listened maybe I would have been safe.

**FGD Number:** FGD_41-F84

**Site:** Malawi

**Excerpt Range:** 76583-77945

I: What could be the reasons that would make the women in our communities to use the methods?

[Triza]: The way that ring looks…am not sure if it is soft…[giggling]

I: Touch it…[interviewer passing the ring to the participant to feel it]

[Triza]: [giggling] Yeah.

I: You see it can be folded into the shape of the number 8.

[Triza]: Yeah… it can be folded…but the reasons could be like... people in the village would say, ‘eeeh so this thing should be inserted in the vagina, what if it goes deep…[giggling]…and never come out…it can enter in the intestines, can’t a person die?’…so such could be some reasons people can raise concerning the ring…like pills, some say that contraceptive pills just lodge in the stomach , so others might mention the same reason…that it may go deep or what if it makes you have continuous menstruation…as you have said it is a drug.

I: Yes, it is a drug.

[Triza]: Yeah, so some may insert then start having continuous bleeding … so with such challenges I don’t know how you look at it.

[Debora]: Just as [Triza] has said, some people might have thoughts like, what if insert this thing can’t I suffer from cancer of the uterus…because some people are talking about many things…some say that if one inserts a loop [Intra Uterine Device] it disappears so what of this, wont it get lost inside?...they might have such views.

**FGD Number:** FGD_41-F84

**Site:** Malawi

**Excerpt Range:** 78203-78399

you have told us some of the reasons why some people might refuse to use the methods…are there any other reasons someone would want to use the method?

[Memory]: The main reason is the protection.

**FGD Number:** FGD_41-F84

**Site:** Malawi

**Excerpt Range:** 78401-80540

I: Who else apart from the doctor, that is responsible for making the decision whether a woman can use or not use the ring or pill during pregnancy or breastfeeding in our communities?...who is also in the position to make a decision whether you use the thing or not?

[Alinafe]: The one that can help you in making the decision is the husband.

I: Okay someone’s husband…yes [Debora]…or you wanted to say the same…is there anyone with a different answer?...it could be our grandparents in the village who have more powers than any other person…is it the marriage counsellor or your neighbour?

[Debora]: It is the husband.

I: So it’s the husband apart from the doctor…how about our mothers, our mothers-in-law,…what is their role in this issue?...are they not supposed to be in the position of telling us to go and use the methods or not?

[Triza]: They can have a say…if they can hear about the availability of the methods they can take a role, as their child or child-in-law…because this thing, one might take it whether known or secretly, you might fall sick and they might say we knew it…as you know parents…the way people talk in the village…they are different.

I: How about traditional birth attendants…witchdoctors…religious leaders …do they have any role on this?...what role can they take on whether a woman uses these two methods…traditional leaders…from the way things happen in the communities?

[Triza]: They might take a role on the drug… maybe if the drug is dangerous, so don’t take it… religious members might be told…they announce in the church even about contraceptives…they do announce and would say there are some drugs and are dangerous, so women don’t use them…so the leaders can be a hindrance so that people should not like the methods.

I: Is there anyone with different views from the ones shared before we proceed … we are about to finish.

[Debora]: I think on this issue the choice rests on the woman…because even if the religious leaders announce in the church as [Triza] has said, but if you are having the conviction in your heart, you just need to do the way you feel it everyone has their own life.

**FGD Number:** FGD_41-F84

**Site:** Malawi

**Excerpt Range:** 80817-82729

[Debora]: The way I understand the question…I don’t know if I understand it well …I think reaching out to people…like in our village they can mobilize people telling them that the chief is calling for a meeting, and women can go there, and someone can explain to them that, ladies we have these, how are you receiving this as women?...just like the way you are asking us here…I think that should be the way women should be reached out…I don’t know if I understood the question.

I: Yeah, it’s like that…you understood it well…have you all understand the question?...what should be done with an aim of encouraging pregnant women and breast feeding mothers to use the products…you have already said that going through the traditional leaders…the traditional leaders should be approached and they should be the ones to talk to their subjects…is that so?

[Debora]: Yeah.

I: I heard you right… and you have responded accordingly, and so have you also [referring to the previous speaker-Memory] …Now in your views…as people who are doing this research we are asking you a question … for us to find other women to be participants in research to use these two products…those that are breastfeeding or pregnant, what should we do? ...what ways should we use to get these women?....we would like to get the views from you women who are pregnant or breastfeeding

[Debora]: I think going to the clinics especially on days when women go for clinic, pregnant and breastfeeding women, so you can educate them and explain to them what the research is all about…those that will welcome it will take it and those that will not they will ignore it.

I: So, it should be in the clinics?

[Debora]: Yes.

[Maggie]: The other way is to go to their communities …where we are coming from…like mobilizing a meeting at the chief’s place because the way they would explain it will be different from how you would do it yourself…eheee.

**FGD Number:** FGD_41-F84

**Site:** Malawi

**Excerpt Range:** 83368-83920

[Triza]: What I would add is that, it’s all up to you to do things so that people should know that the drugs are available and are being used and are really helping…if people like us go they can’t listen to us, but you should come, drop from a car and talk to the people [ all laughing ]…yeah you see people have belief in things differently…some believe because they have seen a person while others believe because they have seen the drugs…so we have different levels of belief…so the issue is thinking about how you can make people like the product.

**FGD Number:** FGD_41-F84

**Site:** Malawi

**Excerpt Range:** 84790-85345

[Debora]: I would speak like this…all this is focusing on making people’s lives healthy especially pregnant and breastfeeding women who chose to either use the pill or the ring are helping their marriages to be healthy in the area of HIV because it is better to prevent than not to… when one has built a fence or has raised a dog in they aim at protection…so if the woman has made a choice to use the pill or the ring you are protecting your life and also making your family healthy…maybe the issue of HIV will decrease as compared to how it is now…sure.

**South Africa:**

**FGD Number:** FGD_41-F21

**Site:** South Africa

**Excerpt Range:** 71164-73501

I: Okay, the video has finished playing. The vaginal ring that we have shown to you, there are women currently who are attend the study in this clinic and using the ring. We have started a long time ago to research about the ring. At first there were given two study rings, one with medicine and the other without medicine. As researchers in this clinic we didn’t know which ring a woman was given, whether with or without medicine, that we didn’t know. Then the results came out showing that the ring protects against HIV. So currently women who attend at this clinic are given a ring with medicine because it is known that it protects from getting HIV. You insert and leave it inside your vagina for a month, you change it after once month and insert a new ring, so you insert it once and change it once, you will have your periods and have sex while the ring is there inside you. Some women say they don’t feel the ring inside of them, they even forget it is there right. Oral PrEP is Truvada known to protect against HIV. Before Truvada was used by people who are HIV positive and now Truvada is used by people who are HIV negative to protect themselves against HIV, right. So now we are playing this video to get your views maybe you have some information some have heard about the vaginal ring and others have not heard about it. Some have heard about oral PrEP and others have not heard about it. So we wanted everyone to be on the same page no to say I have never heard about these thing or I don’t know what the ring looks like, yes. Asanda?

Asanda: Can I ask you say this ring has medicine…

I: It has medicine inside, so when you inserted it releases the medicine slowly into your body to block the entry points…Like in the vaginal walls to block the entry for HIV.

Apple: If you say others forget about it, what if you forget it for a month and you go into your periods with it and also have sex with it, like its normal to forget about it. What is going to happen when you…

I: Okay I am going to request Tumelo [pseudonym for a Note Taker] you to write down all your questions and we first have a discussion, when we are done we will address them. Are there more questions?

Ngwanenyana: What happens if you forget to change your ring?

I: As Apple has also asked, okay. Are there more questions?

Respondents: [Silence].

**FGD Number:** FGD_41-F21

**Site:** South Africa

**Excerpt Range:** 73502-74426

I: Are you okay, we will come to your questions right. Let’s start talking about daily oral PrEP tablet, what is your first thought when you think about taking oral PrEP to prevent HIV while pregnant?

Asanda: It’s after effects would make me worried about what it does in my body while I am pregnant, is it not affecting my baby? Yeah.

I: What kind of side -effects are you thinking of, if you can share with us, which side- affects you think a woman can experience while pregnant. I’m not only directed to her any one can say what side- affects you are thinking of?

Asanda: I think vomiting or dizziness such things.

Ngwanenyana: Maybe a headache.

I: Others what are your thoughts about taking oral PrEP while pregnant?

Red: I think because we don’t know if you take oral PrEP while pregnant whether it will affect the baby, whether the baby might come out numb, or small or what.

I: Others?

Respondents: [Silence].

**FGD Number:** FGD_41-F21

**Site:** South Africa

**Excerpt Range:** 74427-75864

I: Would oral PrEP be something you would be interested in using while pregnant or breastfeeding?

Ngwanenyana: Hence I have said before that I don’t take any medication that is not prescribed by my doctor. So I don’t think I would be interested in using it unless it is prescribed by my doctor that I must use.

Red: I think if they can say okay PrEP is allowed to be taken by pregnant or breastfeeding women I think I would be okay.

I: Why?

Red: Just because PrEP prevents someone from being infected, yes.

I: Others, would you be interested?

Asanda: For me I don’t think so because it’s the first time I hear about it and I am not quite sure how it works unless I have observed it from someone that it treated her alright, so I don’t think so.

I: What worries do you have about using oral PrEP and why, besides the side- effects, besides that it might affect the baby to come out not right?

Pink: As you know people are not the same so it might work for instance for Ngwanenyana and for me it takes its time or it doesn’t work at all.

I: PK what do you think?

PK: What was the question?

I: Taking PrEP while pregnant or breastfeeding, do you have any worries about taking it while pregnant or breastfeeding?

PK: Yeah, it might not work for me…

I: How?

PK: You will find that maybe you forget to take it.

I: Mpho?

Mpho: I don’t think I will have a big problem with it especially if it helps me prevent HIV/AIDS, yeah.

**FGD Number:** FGD_41-F21

**Site:** South Africa

**Excerpt Range:** 75865-77950

I: Do you think it can be difficult to take the tablet every day?

Apple: Do you take it twice or…

I: No, once a day?

Apple: It won’t be because as I said earlier if you start taking it let’s say you will take it at 8 a.m. it will be your daily thing, every day at 8 a.m. you know that I am supposed to take this pill, so I don’t think it’s going to be difficult. Hence probably for two to three days it’s going to be difficult but you need to tell yourself and set a reminder for yourself so that you can know that at 8 a.m. you have to take it, then it won’t be difficult.

Ngwanenyana: I was going to say it can be difficult for you to take your pill daily because sometimes you found that at 8 a.m. as Apple just said you are at work and busy, you have too much work, you cannot go to the kitchen and get yourself some water to take the pill, so it can be difficult at some point.

Apple: Not really because you know that every day at 8.a.m. so you have to have a bottle of water with you, so it’s not that difficult. If it’s a daily thing then you can’t.

I: Okay it’s her view and your view. You have spoken about the timing right, so now what do you think of the size and taste of the pill?

Apple: We don’t know the size of the pill.

I: They did show it here, didn’t you notice?

Apple: Which one?

I: Yes, Red?

Red: I don’t think that pill is a pill that you can be scared to swallow as you can see it’s not that long or big, it’s like a disprin, like when you take a disprin…

Respondents: No, it’s not like a disprin.

Red: You can swallow it fast, it doesn’t have after taste or make your mouth taste bad, and it is just a pill.

Lerato: I think size doesn’t matter as long as you know you are safe and you benefit from it, yeah things like that.

I: You were talking this side, what were you saying Apple?

Apple: Mmh size, yhoo! [An exclamation] No.

R: Do you think you will get sick?

Apple: Not really but it will take time to adjust to it because whenever you are just looking at it you just get sick then how much more when you swallow it, so it will take time.

**FGD Number:** FGD_41-F21

**Site:** South Africa

**Excerpt Range:** 79768-80896

I: Now let’s talk about the vaginal ring what is your first thought about using the vaginal ring to prevent HIV while pregnant?

Asanda: I think of discomfort, I just cannot be comfortable because it looks like it’s hard so I might feel it inside. I don’t think so.

I: Okay, Asanda says so, what do others think?

Apple: For me personally I wouldn’t use it during pregnancy because obviously you would be uncomfortable and so on but after pregnancy since I have watched the video it’s more like a tampon in a way. So when you are used to a tampon the ring won’t be that difficult hence I say you can go on your periods while it’s still inside so it won’t be that difficult.

I: Mmh, how would the ring cause discomfort feelings during pregnancy, what is the difference during pregnancy?

Apple: Because you are uncomfortable already about everything, your body changes more often, the baby is moving and so on so your thoughts are filled with what if I am going to labor and maybe I push this thing [the ring] hard and it disturbs the baby, you have got all those things in your head so yeah you won’t be comfortable throughout.

**FGD Number:** FGD_41-F21

**Site:** South Africa

**Excerpt Range:** 80974-82449

I: What about using the vaginal ring while breastfeeding, when a breastfeeding mother uses a vaginal ring to protect herself against HIV?

Ngwanenyana: Since it was explained that the ring protects only the vaginal part so I don’t think it’s going to have an effect on the child while breastfeeding because the medicine doesn’t go up to the breasts it only settles on the vagina.

I: Who wanted to say something this side, Lisa?

Lisa: My worries are only about cleanliness that this thing is going to be in there for the whole month, I am breastfeeding, I am a new mother…There is some blood coming out, there is some dirt coming out how will it be inside there, what does it contain and when all those things come together will that not cause me vaginal infection, yeast [thrush] or something.

I: Okay. PK what do you think of inserting this vaginal ring while breastfeeding to protect against HIV, PK?

I: Yes, Mpho?

Mpho: Because I heard on the video they say if you insert it at times you forget that it is there, I think we worry about discomfort but if you don’t feel it there, then there is no problem.

I: Okay. What worries do you have about the ring and why, do you have any worries about using the vaginal ring. Lerato?

Lerato: Not really because its 100% guaranteed that it is safe I just worry about the discomfort that’s all.

I: Yes Mpho?

Mpho: I am not sure how big it is but what if it falls out during the month or cause the breastmilk to be bitter.

**FGD Number:** FGD_41-F21

**Site:** South Africa

**Excerpt Range:** 82450-83875

I: Do you think using the vaginal ring will interfere with your daily life?

Lisa: If the vaginal ring is inside and people are able to forget about it because…As Apple said it is like a tampon, so I don’t think it can interfere. Even the tampon is worse because it gets full and you can feel it is full but the vaginal ring stays there for a month so I don t think it can interfere with your life. But as Mpho has said if it can fall out, yeah.

I: So when you look at pregnancy what do you think is the right time for someone to use the ring, first, second or last tri-mester?

Ngwanenyana: I think since pregnant women are not allowed to insert anything in your vagina while pregnant so I don’t think you can use it while still pregnant.

I: Mmh, oh pregnant woman is not supposed to insert things in the vagina while pregnant?

Ngwanenyana: Yes.

I: What do they say happens? I am almost done I am going to the last page of my questions so if you can talk now and we will finish soon?

Ngwanenyana: It is not like they explain what happens when you insert things in your vagina while pregnant but doctors and gynecologist always advise us not to insert anything in the vagina.

I: Okay. Red?

Red: Some doctors do say if you insert some things here, those things do affect the head of the baby and the baby might become slow and the baby’s eyes might have a discharge, and it can lead do wounds and a baby will end up blind.

**FGD Number:** FGD_41-F21

**Site:** South Africa

**Excerpt Range:** 86105-87417

I: Okay. What are other local taboos or practices will make using vaginal ring while pregnant difficult, are there any taboos you know of in your community that may make a person or you unable to use the vaginal ring while pregnant?

Ngwanenyana: I don’t think there are because I think the ring is the new product in our communities so most people don’t know about it, that’s why there are not.

I: Okay. Would pregnant women in the community want to use products like this, in your opinion about other women where we live?

Apple: Not really because there are many stereotypes, there was this female condom it still exist but then most of the women don’t know about it. Or how to use it because it is a stereotype of saying you have to wait for certain hours before you sleep with the guy, so which they say it is useless because the[male] condom you just insert and have sex. Why with the female you have to wait for certain hours to use it, so hence for this one it will also take time for people to intend to use it, because there is a female condom that people don’t use it at all. And now they have introduced Max male condoms with flavors and so on and they are like boom, everyone knows about them because they have got flavor and so on, while female [condoms] they don’t take them into consideration.

**FGD Number:** FGD_41-F21

**Site:** South Africa

**Excerpt Range:** 87418-89284

I: Okay. Do you think women would prefer to use a vaginal ring or take oral PrEP.? If they are told to choose do you think they would prefer a vaginal ring or oral PrEP?

Apple: It will depend okay since this oral [vaginal] ring is not famous yet so a lot of people need to be educated about it and also about the pill [oral PrEP] they need to be educated about it. About the ring sometimes guys don’t understand, this thing is going back to the female condom they don’t understand it and totally disagree when it comes to it. Then when you use this VN [vaginal ring] one you won’t tell your partner you are using it, by doing that you can be able to use it. And with the pill [oral PrEP] hence sometime they are saying others will say you will forgot to take the pill because you have to take it like daily, you don’t have to skip a day, so even with that people need to get more information about both of them [these products].

I: Do you have a different view?

Pink: To add on what Apple said about people forgetting the pills as people…Let me make an example about the contraceptives. There is a pill and an injection, and the injection you choose you want it for how many months. So when it comes to a vaginal ring and PrEP, there will be people preferring PrEP and those preferring the vaginal ring to avoid saying I forgot to take it, you see.

I: Mmh, okay.

Apple: And the other thing with that we as people tend to be ignorant in a way because okay this month you will put it on, you will comfortable and don’t feel anything then you say let me try it, you try the ring and go on for the whole month and you have your periods and having sex all that. And in the next month you forget to remove it because everything was normal with you, you didn’t feel anything, so the problem will be there that you experience side-effects if you forget to remove it.

**FGD Number:** FGD_41-F21

**Site:** South Africa

**Excerpt Range:** 89285-90292

I: How much protection would you like these products to have?

Apple: Meaning…

I: If they use PrEP or vaginal ring, how much would they want it to protect them against HIV?

Apple: Hence it goes back to there is nothing that is 100% right.

I: I don’t know I am here to learn?

Apple: Yeah there is nothing that is 100%, by saying that the PrEP and the VN [vaginal ring], the PrEP they are saying it protects the whole body system, and then the VN [vaginal ring] is only protecting the vagina when you are having sex only, so which is PrEP is more accurate than the VN because VN is only used by the females.

I: The vaginal ring?

Apple: Yes to protect the vagina only which is not 100% so the PrEP is the one that we can say is more related to ARV in way is No. 2 ARVs because if you take it on regular basis life goes on as normal.

I: Do you have a different view form Apple’s this side Asanda?

Asanda: No.

I: You are tired you don’t want to talk anymore?

Asanda: The brain is no longer functioning.

**FGD Number:** FGD_41-F21

**Site:** South Africa

**Excerpt Range:** 90293-90986

I: We are done, I am left with a few questions?

Mpho: Because the ring protects on the outside so that it doesn’t spread on the whole body, what if you get like a blood infection and you get a cut thing like that, will it also help? I don’t know it will only help…

I: Okay.

Apple: No the ring is only for the vagina when you are having unprotected sex, from other disease like when you are catching or you get somebody who is…

I: Lisa?

Lisa: Now they are going back and forth about…Apple was saying the ring only protects the vagina part of your body and the pill is 100%, and Mpho’s concern was that you can even contract HIV from the blood so now she was saying it is the same thing.

**FGD Number:** FGD_41-F24

**Site:** South Africa

**Excerpt Range:** 77823-80703

I: Okay as explained before we are interested in getting your opinions about the two different products that women can use for HIV prevention, the daily oral PrEP tablets and the monthly vaginal ring. Let’s start by talking about daily oral PrEP tablet, what is your first thought when you think about taking oral PrEP to prevent HIV while pregnant?

Nonhlanhla: I think it’s alright to prevent while you are pregnant and after…The pill is alright.

I: After when?

[Nonhlanhla]: Like after you have found out that you are pregnant, and you have tested and know about your status then its fine, the pill is alright to take.

I: Why do you say the pill is alright?

[Nonhlanhla]: I fear the other one… [The vaginal ring].

I: What are you scared of?

[Nonhlanhla]: What is it…?

I: The ring…?

[Nonhlanhla]: Yes, it scares me, so I prefer the tablets.

I: Others what do you think of taking the pills to prevent HIV while pregnant?

[Makhosi]: I also prefer the pill I am also scared of it [the ring] what if…Isn’t that the sizes of the vagina are not the same, what if you can’t get it in or while inside it doesn’t come out.

I: Why do you say you prefer tablets, I want us to talk about the pills now and we will then discuss the ring afterwards? I want to talk about whatever things that may make you prefer to use the tablets or not prefer the vaginal ring, let’s talk about those?

[Grey]: Okay earlier when we were sitting on that side and the lady who was reading the informed consent for us she said these pills can help for both oral and vaginal sex because there are those who prefer oral sex to vaginal sex. Then you can help for both oral and vaginal sex.

I: How can they help with that?

[Grey]: They prevent infections through both vaginal and oral sex that is what she said to us. I think it is best to take the pills for when your partner wants to do oral sex they will help you because the ring protects only the vagina.

I: Okay we have heard [Grey], what do others say about taking daily oral PrEP to prevent HIV? Let’s talk so that we can finish.

[Pink]: We agree that it is better to take the pills to prevent…

I: While pregnant…

[Pink]: While and after because when you are pregnant you still have sex with your partner, even after delivery you still have. And you will never know whether sometimes he has got another partner, so it is alright to use pills to protect yourself and the baby.

I: Can everyone take the daily oral PrEP while pregnant?

[Nonhlanhla]: No, not everyone there are people who don’t do well on tablets especially while pregnant, some vomit when they drink something, so it’s not everyone.

I: Let’s talk?

[India]: I am of the same opinion as [Nonhlanhla] has said that many people don’t like the tablets maybe they would prefer the ring.

[Juicylips]: I agree with [Nonhlanhla] people don’t like the pill when they are pregnant.

**FGD Number:** FGD_41-F24

**Site:** South Africa

**Excerpt Range:** 80714-81755

What about while breastfeeding what do you think about taking daily oral PrEP?

[Nonhlanhla]: I think the pills are alright you just take it and swallow it, it doesn’t go to the breast, not this saying that everything you consume goes to the breast I don’t think it is going to go there, maybe it will just protect, it is airtight.

I: I heard [Makhosi] is responding…?

[Makhosi]: The pill is alright because…

I: We are talking about while breastfeeding and taking this pill...?

[Makhosi]: Yes, because it will protect the baby also.

I: I heard you commenting about whether its goes to the breast while [Nonhlanhla] was still talking…?

[Makhosi]: Yes, it does go because it melts and goes there. It goes through the veins right, yes, it goes there, and it is alright.

[Grey]: I think it should be the clinic or hospital that tell you whether you should take it or not while breastfeeding. There might be chances that it affects or not, so I think it should be the professionals who tell you whether it is okay for you take it or not.

**FGD Number:** FGD_41-F24

**Site:** South Africa

**Excerpt Range:** 81766-83241

Would oral PrEP be something you would be interested in using while pregnant or breastfeeding, you here and not other people outside this discussion?

Respondents: Yes.

I: I noticed [Juicylips] seems to be surprised, so as you are pregnant would you take this pill to prevent HIV?

[Juicylips]: It will depend on how it [PrEP] treats me because I may say that I will take it while it will not treat me well. It will need nurses’ involvement so that they could intervene if it does not treat me well.

I: [Pink] What do you think?

[Pink]: With me pills is not my thing, so I cannot promise when I will take or not take it. I think the ring would be better for me.

I: This pill if you take it at 8.am. you must then take it at 8.p.m. all the time?

[Pink]: I might forget you see. It is better if you inserted the ring that’s it, but with the pill no.

I: Do you think you might forget to take these pills at the same time every day?

[Nonhlanhla]: I might forget.

[Makhosi]: I would not forget, I would have to set an alarm to remind me to take them, when the alarm goes off, I would take it.

I: I see [Grey] is like…?

[Grey]: I think you might forget because sometimes you would sleep out, and it would be hard to leave someone’s house you are visiting.

I: Someone’s house, you mean at a boyfriend’s house?

[Grey]: Yes, and then maybe you didn’t take them with you, or you forgot them at home and 8 a.m. has passed you have not taken them, so you will be defaulting.

**FGD Number:** FGD_41-F24

**Site:** South Africa

**Excerpt Range:** 83258-84894

what worries do you have about oral PrEP and why, would women be worried about the baby’s health about taking these pills every day while pregnant or breastfeeding?

[Grey]: Maybe the side -effects from the pills might affect the baby while pregnant.

I: Others?

[Makhosi]: I think the pill is alright because it will protect the baby as well, during pregnant and while breastfeeding, it will protect the baby because they developed it knowing what effect it will have.

[Nonhlanhla]: I am siding with [Makhosi] because we do take pills while we are pregnant like the vitamins and they tell you it is for blood or high blood pressure, so this one will be treated the same, for high blood [pressure] to protect the child and make the baby grow, so there will be no worries.

[Grey]: But the immune systems are not the same what if it works for somebody else and it doesn’t work for me…

I: During pregnancy or breastfeeding?

[Grey]: Both. What if it treats another person okay and with me it doesn’t, maybe she can take it and when I take it I have side- effects even though they know that its best it doesn’t mean it’s going to work for everyone.

I: What kind of side- effect do you think of that the pill might have?

[Grey]: Maybe vomiting or maybe severe headache, yeah, fatigue…

I: Are there things you think the pill can cause to the baby if taken during pregnancy or breastfeeding?

[Pink]: You might be worried that the pill will make the baby disabled.

I: While pregnant or breastfeeding?

[Pink]: During pregnancy and after delivery. The baby may be disabled, so yeah.

I: Okay. Any other worries?

Respondents: [Silent].

**FGD Number:** FGD_41-F24

**Site:** South Africa

**Excerpt Range:** 84895-85797

I: What do you think about the size of the pill?

Respondents: Too much… [all talking and laughing]

[Grey]: Bitter…...

I: [Grey] says its bitter, lets listen to [Pink]?

[Pink]: I am saying the size of the pill is too big, and for me I don’t like pills generally. I think it’s too big to swallow and you might feel it, no ways.

[Nonhlanhla]: I am siding with [Pink] its big, you might need to crush it before.

I: Okay, so you think crushing it can help?

[Nonhlanhla]: Yes.

I: Others what do you think you can do to overcome the problem about the size of the pill, what can you do if you were to take it?

[India]: I would break it into two pieces, take one and take another one.

[Pink]: It could have been better if they made it in a powder form, than this pill form.

I: Okay. [Juicylips]?

[Juicylips]: The size is big maybe if you can put it in water.

I: To dissolve it?

[Juicylips]: Yes.

**FGD Number:** FGD_41-F24

**Site:** South Africa

**Excerpt Range:** 85798-86564

I: When do you think it would be suitable to take this pill, during the 1st, 2nd, or 3rd trimester?

[Grey]: From 2nd trimester.

I: Why 2nd?

[Grey]: When the foetus develop and it has become strong a bit.

I: Why at such a time?

[Grey]: Is not that the foetus during the 1st trimester is still small, they say it’s small as a size of a plum. I think in the 2nd trimester around four months when the foetus has developed a bit.

[Nonhlanhla]: I think immediately when you go and book [at the antenatal clinic], after you found out about your [HIV] status, if you are [HIV] negative you must start taking the pill. And if you found out that you are positive they start with the pills to protect the foetus, so they are fine after you found out you must take it.

**FGD Number:** FGD_41-F24

**Site:** South Africa

**Excerpt Range:** 86565-87302

I: How do you think taking this pill everyday would interfere with your daily life?

[Grey]: You must make it a priority because you must take it at the same time, right, so you must now know you have to take it, it’s your life because it’s going to protect you and the baby so…

I: So how are you going to make it a priority?

[Grey]: Whatever you are doing must wait, whether you have to go somewhere you must first take the pill.

I: Does anyone have a different view from [Grey]?

Respondents: No.

[Makhosi]: I think it’s okay if you set an alarm, wherever you go you take your pill and put it in a purse, when the alarm goes on you know what that is for, because worrying about other people will not help you at all, it’s your life.

**FGD Number:** FGD_41-F24

**Site:** South Africa

**Excerpt Range:** 87303-89025

I: How do you think this pill will interfere with women’s use of contraceptives?

[Pink]: I think if we can ask a doctor how it can interfere with what you will be using whether pills or injection, so it would be better to talk to a doctor first.

I: But do you think daily oral PrEP can interfere with your use of contraceptives?

[India]: I don’t think so because with oral PrEP, you will use it daily and contraceptives you will use them once a month and you will skip other months, so I don’t think there will be a problem.

I: Okay. If a woman is using this pill daily [PrEP] and use the contraceptive pill daily, do you think that can cause a disturbance or a problem?

[Nonhlanhla]: There could, because you will have to set a certain time for one pill and another time for the other pill, you see now you must set the alarm for one pill and another time for the other pill…

I: If someone is using the contraceptive pill…?

[Nonhlanhla]: Yes, the contraceptive pill.

I: What about a person who is using contraceptive injection like the 2 months or 3 months one. Will there be a disturbance or problem?

Respondents: No.

I: If someone is using the injection and the pill?

Respondents: Yes.

I: Why then should there be a disturbance or a problem when you take PrEP and the contraceptive pill?

[Nonhlanhla]: Because you will be taking the pills every day, and you will have a time for the PrEP and a different time for the oral contraceptives, so for the injection, you only have it after two months and you know on which date you must go for your injection, that’s it, you are only busy with the PrEP.

I: But what kind of disturbance would be experienced?

[Nonhlanhla]: There is a possibility of forgetting.

**FGD Number:** FGD_41-F24

**Site:** South Africa

**Excerpt Range:** 89035-90345

Depending on the culture it may be accepted or a taboo to take bitter medicine during pregnancy. How will that interfere with women’s ability in your community to take oral PrEP while pregnant? [Grey] said the pills are bitter, will it be acceptable or taboo for a woman to take this pill every day to prevent HIV while pregnant, what do you think?

[Makhosi]: I think that with the pill you just swallow it and you are done.

I: As people in our communities have certain beliefs, will be a taboo or acceptable to take this pill as one of the bitter medicines, while pregnant?

[Pink]: I think if its bitter the community will think it’s working, because normally people perceive a bitter medicine as effective and working, that’s what I think.

I: Mmh, are there bitter medicines that pregnant women in our communities take while pregnant, let’s talk about that?

Respondents: Yes.

[Grey]: Yes there is this thing…Baboon’s urine, it has a bitter taste, but it is well known and common knowledge that people use it and it helps them.

I: I hear [Makhosi] talking about “Isihlambezo,” is it also bitter?

[Makhosi]: Yes.

I: Okay, what do you think of what [Pink] has said that the community will think this pill is working, because people think bitter medicine or pill is working?

Respondents: Yes, we agree.

**FGD Number:** FGD_41-F24

**Site:** South Africa

**Excerpt Range:** 90346-93099

I: Now let’ us talk about the vaginal ring, what is your first thought when you think about using the vaginal ring to prevent HIV while pregnant?

[Nonhlanhla]: It is scary…

I: How is the ring scary?

[Nonhlanhla]: It looks like when you try to insert it you won’t be able to remove it, I just don’t understand it.

I: Mmh?

[Nonhlanhla]: How do you remove it, what if you can’t, what if you inserted it wrongly and it affects you.

[Makhosi]: Or during sex, he pushes it further…

[Nonhlanhla]: And it blocks...

[Makhosi]: And it blocks…

I: It blocks where?

Respondents: We don’t know.

I: What do you think?

[Pink]: I think the ring is alright and I don’t think it can block because it’s a rubber and it is round, so when you insert it and it gets there it will be the way it is as it is round, so I don’t think it can block. And, when you are having sex I don’t think it can block. I also think every time you insert or remove it you need to wash your hands because you don’t know whether your hands might be dirty, and it can go in with infections, because the vagina is sensitive.

I: How do you feel about washing your hands first before you remove it, do you feel like it’s a right thing to do or its going to be a burden?

[Makhosi]: It’s a burden

I: [Makhosi]: Says it’s a burden…?

[Pink]: For me it’s not a burden like setting up an alarm for a pill, I think that’s a burden. I think the ring you will just have to wash your hands because the vagina is sensitive you can’t just put in your dirty hands.

I: What do you think?

[Juicylips]: I prefer the ring.

I: Why do you prefer the ring?

[Juicylips]: I think it’s safer than taking the pill, because with the pill you will forget to take it.

I: For now, we are talking about using the ring while pregnant, what do you think of that?

[Pink]: I think it’s not right while pregnant because you will have to always insert your fingers every month. I am just thinking, maybe you will never know whether you are hurting the foetus, I don’t know whether when you insert the finger the womb will bleed and at the hospital they tell us you can’t always put the fingers in. I don’t know whether you might be hurting the foetus, so while pregnant I don’t think it’s right, but after delivery you can use it.

I: What do others think of using this ring while pregnant?

[Nonhlanhla]: I think it’s better to use it after delivery.

I: Why?

[Nonhlanhla]: I fear it.

I: What are you scared of?

[Nonhlanhla]: Always inserting fingers, no I don’t like it.

[Juicylips]: It is right because when you insert your finger in the vagina you will be removing dirt.

I: Mmh?

[India]: I agree with [Nonhlanhla] and [Juicylips], it is alright but putting the finger inside the vagina doesn’t go well with me.

**FGD Number:** FGD_41-F24

**Site:** South Africa

**Excerpt Range:** 93100-93926

I: What do you think of using the vaginal ring while breastfeeding?

[Pink]: I think it will never affect you while breastfeeding because you will be inserting it in your vagina instead of swallowing it, as they say whatever you consume comes to the breasts [breast milk]. With this one you insert it in your vagina and it cannot affect the baby when you are breastfeeding.

I: Others?

[Makhosi]: I prefer pills, you not always inserting fingers in your vagina, no.

I: Okay.

[Makhosi]: The pain you will feel in the vagina…

I: Do you think inserting the ring will be painful?

[Makhosi]: Yes.

I: What do others think of the ring, is it something you can use, or other women can use while breastfeeding?

[India]: I think I can use it while breastfeeding as long it stays where it stays for that month, then it will be okay.

**FGD Number:** FGD_41-F24

**Site:** South Africa

**Excerpt Range:** 93927-94964

I: What worries do you have about the ring and why?

[Nonhlanhla]: My first worry is that you will insert it and then it doesn’t want to come out, and you will be searching for it and don’t know where to search then you must go to the doctors to search for it…They will be navigating your vagina more.

[Makhosi]: And you might be hurting yourself.

[Nonhlanhla]: You might be hurting yourself, then you must go to the doctors and they will be navigating your vagina more, that’s my main worry.

[Grey]: I agree with her. Do you remember the time they had just introduced the Implant? Others were saying it disappeared from their arms they could not locate it. So, what if it dislodges and you can’t find it, I agree with her.

I: Okay, any other worries about using the vaginal ring?

[Makhosi]: Maybe while having sex he will be pushing it further and further, it goes up and up…

[Nonhlanhla]: Until it gets lost.

[Pink]: I want to know if you have inserted it how will you know that its sitting properly where it’s supposed to sit.

**FGD Number:** FGD_41-F24

**Site:** South Africa

**Excerpt Range:** 96815-97106

how do you think inserting the ring in the vagina can interfere with sex?

[India]: I think your mind as you are having sex, your mind will be on the ring, and while he is busy performing [sex], you will not be comfortable.

I: So, it will affect the sex mood.

[India]: Yes, your sex mood.

**FGD Number:** FGD_41-F24

**Site:** South Africa

**Excerpt Range:** 97107-99120

I: How do you think the ring will interfere with your daily life, you said with the tablets someone needs to set up an alarm and when it goes on she must go and take the tablets, what about the vaginal ring?

Respondents: [Silent].

I: I heard [Pink] earlier saying you can insert the ring for months and it stays there that’s it, unlike the pill that you must take every day, but what do others think, do you think it can affect your life?

[Grey]: I don’t think so because the video had explained that it will never interrupt your daily life, I don’t think it will cause any discomfort.

I: During pregnancy or while breastfeeding?

[Grey]: I am not sure about that.

[Makhosi]: I don’t want to lie…

[Grey]: What if you have it inside and you go into labour…

[Makhosi]: And the baby comes out holding it in the hand.

I: [Makhosi] thinks during delivery the baby will come out holding it in the hand, and [Grey] what are your concerns if a woman goes into labour while she has the ring inside.

[Grey]: I think it will affect…Unless maybe you tell them when you arrive at the hospital that you have the ring and then they will take it out.

I: Let’s say there is no time and the labour pains are so severe you can’t even talk, how do you think the ring will interfere with delivery?

[Juicylips]: Before you deliver, they do check you how many centimetres, obviously, when they put a finger in they will feel it.

I: Okay.

[Nonhlanhla]: What if I am at home and have labour pains and deliver there, it will strangle the baby, how will the baby come out, the baby must come through that tube, yes it will interfere.

I: If you say the baby is supposed to come out of that tube what do you mean?

[Nonhlanhla]: It will block the baby, cause of infections and all that, and hurt the baby because you would have forgotten because labour pain will be heavy on you.

[Makhosi]: Yeah you will forget it, you know when you experience labour pains you are just not focussed.

[Nonhlanhla]: And you are busy pushing.

**FGD Number:** FGD_41-F24

**Site:** South Africa

**Excerpt Range:** 99392-101772

I: Depending on the culture it may be accepted or a taboo to insert things in the vagina while pregnant. How will this affect women’s ability in your community to use the vaginal ring while pregnant?

Nonhlanhla: I think it may be taboo because people will not understand you inserting things in the vagina while pregnant.

I: What will people think?

[Nonhlanhla]: They will think otherwise…

I: How?

[Nonhlanhla]: Because culturally when you are pregnant you don’t insert anything, you can only drink things, so when you insert things in the vagina is just something else.

I: What do you mean by saying people will think otherwise?

[Nonhlanhla]: They will have the same questions we have like what if you go into labour, how will the baby come out, you insert tubes, you see those things, and it will affect you.

I: What do others think, will it be taboo to insert the ring in the vagina while pregnant?

[India]: I agree with what [Nonhlanhla] is saying because in the first place they will be surprised what this woman is doing, they will ask many questions. Or they won’t even think otherwise, instead they will ask you if you want to abort the baby, that’s another thing, they will ask what is it, do you want to abort…

I: Okay so they will think you are doing termination of pregnancy?

[India]: Yes, that’s why you must go to the toilet and lock yourself [when inserting the ring].

[Juicylips]: Okay how are they going to see you inserting it because you insert it alone? And if you are going to deliver the baby at home the ring is like rubber it can shift, and the baby can come out. When the baby comes out obviously, it will also come out.

[Makhosi]: No, it won’t because they say it stays like this

[Juicylips]: Yes, they say it stays like this, but as the foetus head pushes out it will also turn like so…

[Makhosi]: How will it turn…?

[Juicylips]: It will go down and the baby will follow.

[Pink]: Can I ask a question? What time are we finishing the discussion?

I: We are finishing… [Nonhlanhla] what do you say?

[Nonhlanhla]: [Silent]

[Juicylips]: [Silent]

[Makhosi]: She says it’s going to turn, that’s what I don’t understand, how does it turn.

[Juicylips]: As you insert it, it stays there. This thing is a rubber, the baby’s head is here, and the baby is coming and pushing it, and what the ring does, its slides on the side as baby pushes out…

**FGD Number:** FGD_41-F24

**Site:** South Africa

**Excerpt Range:** 101798-102237

what do you think about using the ring while breast-feeding?

[Nonhlanhla]: I think it’s alright while breastfeeding…

I: Why do you say so?

[Nonhlanhla]: Yeah because you are done with everything, maybe after one month your stitches have healed, you can be able to insert it and remove it.

I: After the stitches, have healed?

[Nonhlanhla]: Yes, when you are alright.

I: Others, any different views?

Respondents: No, we agree with her.

**FGD Number:** FGD_41-F24

**Site:** South Africa

**Excerpt Range:** 102248-103580

would pregnant women in your community want to use products like these, I am talking about oral PrEP and the vaginal ring?

Respondents: Yes.

[Pink]: Two products at the same time…

I: No, one product by one woman and another product by another woman?

Respondents: Yes, they can.

I: Why?

[Pink]: Because, we also agree here that we can, as I also said, I can also use this ring, others said they can use the pill, so other women out there will have a choice whether to use the ring or the pill.

I: What about while breast-feeding?

[Pink]: I think they can as I said I could use the ring while breastfeeding, it doesn’t have a problem, but I think the pills might cause a problem to the baby...

I: When…?

[Pink]: When breastfeeding and taking those pills [oral PrEP].

I: Mmh, what problem can they cause?

[Pink]: The baby might get sick or might not treat the baby well so it’s better to use the ring.

[Nonhlanhla]: I think the pill is okay because after delivery we drink “Imbiza” [herbal mixture]and what not, and say we are cleansing the blood, it must all come out, so the pill is also okay because it will not affect the foetus now.

[Makhosi]: I think women will choose what they want to use, I think you are not going to choose for them, they will have to choose and at what time they want to use these products.

**FGD Number:** FGD_41-F24

**Site:** South Africa

**Excerpt Range:** 103581-103753

I: Amongst yourselves here, how many would prefer to use the ring, Two

Respondents: Yes.

I: One has gone out to the bathroom, and this other three.

Respondents: Pill.

**FGD Number:** FGD_41-F24

**Site:** South Africa

**Excerpt Range:** 103764-108532

besides your doctors, who should be involved in deciding whether a woman uses one of these products while pregnant or breast-feeding?

[Pink]: Your partner or boyfriend must have a say when you are pregnant, you shouldn’t do things alone and maybe at the end the baby gets hurts and he blames you, you both need to discuss and agree whether you should use the products maybe after delivery or while pregnant.

I: So, during pregnancy, then who should make decisions for while breastfeeding, your doctor or your partner, or other people, or family members, or whoever you want to involve in your decision?

[Pink]: I think the doctor if you must use it or not use it.

I: You are saying the doctor and your partners should be involved?

[Pink]: Yes.

I: And others would you take your own decisions without the approval of the doctor, or partners, or whoever you normally involve in your everyday decisions specifically now about the vaginal ring or oral PrEP?

[India]: I think the way [Pink] has said it, I think both your partner and the doctor…

I: While pregnant or breastfeeding?

[India]: Both periods.

I: Okay. What about your mother or mother-in-law?

Respondents: No.

I: Why are you saying no?

Respondents: They have no part in this.

I: They have no part?

Respondents: Yes.

[Nonhlanhla]: But you must inform them so that they can help you….

I: [Nonhlanhla] has a different view?

[Nonhlanhla]: I think you must inform them because after delivery, you go to your parents’ home to nurse the baby and there are pills that you need to take at a certain time and you had difficult time sleeping at night, and there was someone who was helping you. Now at 8 p.m. you must take the pill, so if don’t inform her what is she going to think about the pill, always at 8 p.m. you must take it. That’s when they will gossip about you and suspect that you are positive, why is she not telling us, you see.

I: When you take oral PrEP?

[Nonhlanhla]: Yes.

I: What about the ring?

[Nonhlanhla]: With that one its okay even if you don’t tell them because it’s something inside you, it’s okay.

I: What about traditional healers, if I consult a certain female or male traditional healer is it important for me to tell them that I am using oral PrEP?

Respondents: No.

I: Or the vaginal ring, why?

[Makhosi]: What business of them is there?

I: In your vagina? What about the pill, maybe they want to help you with certain things, do you think it’s important to tell them that I am taking the pill or inserting the ring to prevent HIV?

[India]: I think it is right to tell the traditional healer you are using the ring or a pill, imagine she will give you that concoction to drink and it will take away the effectiveness of the pill.

I: What about the pastors, we previously mentioned that pregnant women also go to the prophets to get prayers for them and for the baby. Is it important to tell them that you are taking the pill or using the ring?

[Juicylips]: You must tell the prophet because she or he will prepare something too strong for you and it will take the medication out.

I: The prophet?

[Juicylips]: Yes.

I: What about the churches that don’t use “Isiwasho” (cleansing mixture)?

[Nonhlanhla]: You shouldn’t tell them because they also don’t tell us, they just say we will pray, they don’t tell you, they are not even interested to know what we eat they just concentrate on prayer.

I: [Grey]?

[Grey]: I think it doesn’t make a difference whether you tell or not tell them, in other churches they will give you something that will affect…

I: In other churches they give you something, they have just mentioned “Isiwasho,” now?

[Grey]: Eish…

I: You are saying is the same whether you tell them or not tell them, traditional healer or a pastor?

[Grey]: Yes.

[Makhosi]: My sister was attending this thing, which also appears on TV [television set], these things of traditional healers, you know them, on SABC 1[television channel]. Like when they take on an initiate, they first test them as it is now approved that they do that when they have initiates. If she gives this person, something to drink it will not harm the person, if the person is HIV positive they know what to do, because they also get trained…

I: So, what are you saying about the pill and the ring, are you saying people should inform the traditional healers they are consulting, or pastor or prophets, is that what you are saying?

[Makhosi]: I am saying…I am just advising, I would tell them that I am taking a pill for HIV prevention so please don’t give me a strong medicine so that it doesn’t affect me, it doesn’t have a negative effect on me.

I: That’s concerning the pill, what about the ring, would you also tell them that you are using the ring?

[Makhosi]: Yes.

**FGD Number:** FGD_41-F24

**Site:** South Africa

**Excerpt Range:** 108557-109916

What do you think is the most important factor that will motivate pregnant or the breast-feeding woman to use the ring or the tablets for HIV prevention?

[Makhosi]: I think the pill because they might be afraid to insert the ring while pregnant, but they can take the pill.

I: Others, we are done, and this is my last question, do you have your last closing comments?

[Grey]: Because human safety comes first, so to know that the ring and the pill can prevent HIV will motivate people to use the ring and the pill.

I: Anyone with a different view from [Makhosi] and [Grey]?

Respondents: No, we agree with them.

I: Alright, what should we do to encourage women to use these products?

[India]: I think you should encourage them by educating them about these products, as much as we have heard about them when we leave here we should also tell people about them.

[Makhosi]: There must be pamphlets that people can read.

[Grey]: I think it should be put up on social media because many people use the internet and social media almost every day, so people will go into the internet, learn about these products and gain knowledge.

I: What social media are you talking about?

[Grey]: YouTube and these posts we always put up on WhatsApp, and there must be a Facebook page about these products.

[Pink]: And they must educate about them in the clinics.

**FGD Number:** FGD_41-F24

**Site:** South Africa

**Excerpt Range:** 109930-112137

what suggestions do you have on how we can recruit pregnant and breast-feeding women for future studies in which they will use these products, you have mentioned the clinic, pamphlets and social media, are there other ways we can use to invite women to use these products in our studies?

[Nonhlanhla]: You may want to go to the clinics where women book [antenatal clinic] and maybe everyday people come to speak to women because sometimes the nurses are busy, they don’t have time. There must be educators who will tell the women about these products.

I: Pregnant women, what about the breast-feeding women, how can we reach them?

[Nonhlanhla]: They are found in the same place in the clinic.

[Makhosi]: There are also those who are found putting up gazebos who do HIV testing…

I: You mean HIV campaigns?

[Makhosi]: Yes, things like that can help.

[Grey]: I wanted to say what [Nonhlanhla] has said, and again you can use the TV and the radio adverts.

[Pink]: Door to door campaigns.

I: Okay. How can we earn trust of our communities because if you just show up people might be scared, what do you think we can do to earn the trust of the community, let’s say we go to Orlando or Diepkloof [pseudonyms for places] and when we get there to explain about these products to the community while they don’t know us? What can we do for communities to trust us?

[Pink]: Maybe if you as the sister who was teaching us said there are/were women using these products right, and they are fine, so if you can bring them to speak to those people to confirm that they have used these products, and these were their experiences. That would be better rather than you just coming and speaking about these products and people don’t know whether you have used them, and you don’t know whether they will work or not.

I: Any more comments on how we can earn trust in our communities besides using the ambassadors for the ring and the pill as [Pink] has said?

[Grey]: Maybe go to churches as they sometimes have these social events, maybe if someone can go and speak about these products there. The church is the place that people are used to and feel comfortable, then on Fridays some have these social events.

**Uganda:**

**FGD Number:** FGD_41-F61

**Site:** Uganda

**Excerpt Range:** 48586-48824

I: Now, the vaginal ring which we have been talking about is here. You can hold it. Did we understand how the vaginal ring works?

Chorus: Yes.

I: Where do we insert it?

Chorus: Inside the vagina.

I: For how long?

Chorus: For a full month.

**FGD Number:** FGD_41-F61

**Site:** Uganda

**Excerpt Range:** 48825-50309

I: How about the pills?

Chorus: Take them every day for a month.

I: Now, I want us to first concentrate on the pills. What comes to your mind when you think about taking pills for HIV prevention every day during pregnancy? Ritah

Ritah: What comes to my mind is that I am one hundred percent sure that I will give birth to an HIV negative baby. If my partner comes with HIV and we go for sex, immediately after I just get my pill and take. I am one hundred percent sure that I will give birth to a healthy baby (with no HIV).

Angel: What comes to my mind first is that I have taken tablets and I am not going to get HIV.

I: Annet?

Annet: The first thing that comes to my mind is that I have taken tablets and I am safe because I cannot get HIV but I also say, “ what if it has effects and it makes me get a miscarriage?”

I: What if it has some side effects?

Annet: Yes, and I get a miscarriage.

Samantha: I heard that when you take those pills you get a miscarriage. Now, how sure am I that when I take it I will not get a miscarriage. I have seen someone before who started taking it and got a miscarriage. She was about three months pregnant and she got a miscarriage.

I: So, what comes to your mind first is whether you will not get a miscarriage…

Samantha: Yes because I have seen someone who took those pills for prevention and she got a miscarriage.

Pamela: The thing that comes to my mind is that I will give birth to my children without any worries of infecting them with HIV.

**FGD Number:** FGD_41-F61

**Site:** Uganda

**Excerpt Range:** 50310-50900

Sarah: I will know that I will stay safe [HIV negative] even when my partner engages in sex with other women because it will not be possible for him to infect me.

Shanitah: I will be sure that I will stay safe [HIV negative].

Vanessa: I will be sure that I am safe [HIV negative] and my baby is also safe [HIV negative].

Aida: I would stop worrying [about getting infected with HIV] even when my partner engages in sex with other women.

I: How about taking that pill during breastfeeding, would it be the same?

Chorus: It is the same.

I: Maybe there is a difference…

Chorus: It is the same.

**FGD Number:** FGD_41-F61

**Site:** Uganda

**Excerpt Range:** 50901-52138

I: Now, would that pill be something you would have wanted to use during pregnancy?

Chorus: Yes.

R: No.

I: During pregnancy?

Samantha: I would think that a vaginal ring is better.

I: I want us to first talk about the pill, we will also talk about the vaginal ring.

Sarah: I think I would take it because it would stop me from worrying about getting infected with HIV.

Annet: I would think it is not a good option for me because I do not like pills.

I: Why don’t you like pills?

Annet: Just thinking about it I vomit.

Pamela: It is not something I would like to use because I can forget to take it.

Aida: I do not have any problem with taking pills but my question is what if I forget to take it on that day.

I: If you forget to take it?

Aida: If you forget to take it on that day, what do you do?

Samantha: On my side, I would say no to that pill, after seeing the other woman’s experience who got a miscarriage when she started taking those pills I can’t take them.

Vanessa: It might not work for me because during pregnancy I vomit a lot and that makes me doubt whether it can stay inside when I vomit.

Shanitah: I think I would take it to stay safe in case my partner engages in sex with other women.

Angel: I also think I can use it.

**FGD Number:** FGD_41-F61

**Site:** Uganda

**Excerpt Range:** 52139-53745

I: What are you worried about this tablet?

Sarah: We are told that if someone starts taking that medicine and then stop you can die. My question is “Does that happen with only those who are infected with HIV or even those who are not infected?”

I: What did we say the tablet is for?

Chorus: Prevention.

Samantha: I heard that when the blood gets used to medicine it weakens [immunity reduces] if one stops taking that medicine. So, that would mean that in case I start taking that medicine and then stop my immunity will also reduce where it can’t fight off the virus. That would mean that I have to take it forever just like HIV positive patients.

Pamela: What might stop me from taking that tablet, I might have not told my partner that I am taking it and we get misunderstandings when he finds out.

I: What might he think in case he found out?

Pamela: That maybe I have a certain infection that I am treating.

I: How about the rest, what worries do you have about this tablet?

Ritah: Now, in case you are pregnant and you have to take them daily, don’t they have some side effects? Won’t they affect the baby who is inside the womb?

Annet: We are told that HIV can take some time before you get to know that you have it. What if I start taking it when I have it, won’t that cause some problems?

I: That is what you are worried about?

Annet: Yes.

Aida: Doesn’t that tablet cause some side effects if taken daily?

I: Anyone else with some worries?

Sarah: We are told that when you take that tablet it causes dizziness, you lose energy in your body…does that also happen to me who is not infected with HIV?

**FGD Number:** FGD_41-F61

**Site:** Uganda

**Excerpt Range:** 54654-56224

I: At what point should a pregnant woman start taking that tablet?

Ritah: To start taking that tablet depends on when a woman goes to the hospital for antenatal and gets tested whether she is HIV positive or not. When you get to know that you are HIV negative that is when you start taking those tablets.

I: What do the rest think?

Shanitah: I would think that she should have started taking that tablet when she has had some time during pregnancy.

I: How long?

Shanitah: About four or five months of pregnancy.

I: Why four or five months?

Shanitah: Because during those early months the baby is still so young.

I: What do the rest think?

Vanessa: She should start right when she gets to know.

I: When she gets to know what?

Vanessa: That she is pregnant.

I: How many say that a woman should start right when she gets to know that she is pregnant?

Annet: That is what I also think.

Sarah: Me as well.

I: How about Samantha?

Samantha: When the pregnancy is a bit old; I think at month five.

I: Why at month five?

Samantha: I have seen someone who started taking it when she was about three months pregnant and she got a miscarriage.

I: That is why Shanitah also thinks that it should be at least at month four or five to avoid a miscarriage…

Shanitah: Yes.

I: How about a breastfeeding mother, when should she start?

R: She can start right immediately.

R: Because you get to deliver when you had already started taking it. you just continue taking it.

I: How about that woman who got to learn about it when she has started breastfeeding?

Chorus: Should start immediately.

**FGD Number:** FGD_41-F61

**Site:** Uganda

**Excerpt Range:** 56904-58211

I: Now, let us talk about the vaginal ring. What first thoughts do you get when you think about inserting a vaginal ring to prevent HIV during pregnancy?

R: My first fear is that in case I am having sex with my partner can’t that vaginal ring get lost within my body?

I: Getting lost within your body…

R: It moves from where I insert it and it extends inside.

I: That is the first thing that comes to your mind.

R: Yes.

Samantha: What I think of first is whether it doesn’t work like the one for family planning which might prevent me from giving birth [getting pregnant].

Angel: Won’t you experience some diseases [side effects] when you have it? Things like cancer, candida and the like…

Vanessa: What I fear about it…I feel it is big and hard. Can I really insert such a thing? You are telling us that we remove it ourselves but how do I do that? I also think it is large.

Ritah: Is that vaginal ring tied somewhere or it just fixes itself somewhere?

Aida: I would fear to have sex with a man because I would think he might extend it inside.

Pamela: My first thought is, will I stay the same way I am? It is so big and so hard.

I: What do you mean by staying the same way?

Pamela: Won’t my vagina become narrow?

Chorus: [Laughing].

R: The vaginal ring has ARVs in it. Can’t that medicine affect the baby?

**FGD Number:** FGD_41-F61

**Site:** Uganda

**Excerpt Range:** 58212-58906

I: How about during breastfeeding, what do you think? Do you have the same views or they are different?

R: During breastfeeding my baby might not be affected but won’t it affect me? Won’t it cause any infection to me? Remember it stays inserted for the entire month.

Sarah: When you insert it, does it get there and softens or it stays hard?

Pamela: When you insert it, does it melt and reduces in size or it remains in the same size?

I: Is there anyone else with questions about that vaginal ring?

Annet: Won’t I insert it and at the time of removing it I fail to locate it?

Angel: Do I insert it myself or it is a health worker who helps me to insert it?

Pamela: Where is it supposed to stop?

**FGD Number:** FGD_41-F61

**Site:** Uganda

**Excerpt Range:** 58907-59755

I: Is there anything else that you are worried about that vaginal ring?

Sarah: If I have been using it during pregnancy, do I stay using it during that time after delivery when I am not having sex with anyone? So that I resume using it when I resume sex with my partner.

R: You know, everyone has her own opinion. If I have used it for say three years and then one time I do not have it and I happen to have sex with a man when it is not inserted, do I get infected with HIV or the medicine is still in my body? Remember when you use the injectable for about two years, the moment you stop it can take you about a year without getting pregnant not until the medicine is finished in your body. So, is this the same or this is different? If I remove it say now and I get to have sex with a man, do I get infected or the medicine is still in the body?

**FGD Number:** FGD_41-F61

**Site:** Uganda

**Excerpt Range:** 59756-61104

I: But is it something you would have liked to use?

Chorus: Yes.

I: Why would you like to use it?

R: To prevent HIV.

Angel: It doesn’t worry like pills and it keeps it confidential because your partner cannot know that you have a vaginal ring or not.

Samantha: I think it is good because it doesn’t worry like tablets. And in a way you would be saved from the gossip of neighbours because if she entered your house and saw the tablets she will talk about it with the entire village. She will not think that you are taking it for prevention; she will think you are infected. But a vaginal ring keeps it confidential; no one will know that you have it.

Vanessa: It would work for me as an individual but it is too big.

Chorus: [Laughing].

I: How big is it?

Vanessa: It is big in size and it is hard.

I: Why are you scared of the size?

Vanessa: It is big and the man’s penis can just pass through it

Chorus: [Laughing].

I: Tell us.

Vanessa: You told us that we insert it ourselves but how can you insert such a big thing? That thing is hard and big.

R: Do you insert it using something or just the way she is holding it?

R: It is inserted the way she is holding it.

R: So, when it gets inside it unfolds?

R: Yes.

R: What if it fails to unfold?

R: Can’t that vaginal ring fail to unfold?

R: It can’t fail to unfold.

Chorus: [Speaking at the same time].

**FGD Number:** FGD_41-F61

**Site:** Uganda

**Excerpt Range:** 61105-62197

I: Samantha says that it is not bigger than the head of a baby and a baby’s head can go through the vagina. Remember we said that we take all your views because they are important to us. Now, at what time should a pregnant woman start using it?

Samantha: When you get to know that you are pregnant you insert it because I do not think it can cause a miscarriage.

R: When the pregnancy is due, doesn’t it push out that vaginal ring?

Annet: When I am about to deliver, do I remove it or leave it there?

I: When you are about to deliver?

Annet: Yes.

Vanessa: What if it extends inside or fails to get out?

I: I want you to first respond to my question. I asked when a pregnant woman should insert the vaginal ring and Samantha said that it should be immediately after knowing that she is pregnant. What do the rest think?

Angel: When she gets to know that she is pregnant.

Pamela: It would be better when she gets to know that she is pregnant.

I: Is there someone with something different from that? Is there anything different or both of us think in the same way?

Chorus: We all think that way.

**FGD Number:** FGD_41-F61

**Site:** Uganda

**Excerpt Range:** 62198-62589

I: How about a breastfeeding mother, when should she start using it?

R: She should start immediately she gets the vaginal ring.

Pamela: The breastfeeding woman should start at around month four, when the baby is four months because her vagina is by then fully healed.

R: The moment she gets to know that she wants to have sex with a man.

I: Is there anyone with something different?

[Silent]

**FGD Number:** FGD_41-F61

**Site:** Uganda

**Excerpt Range:** 63469-64833

I: Now, would you think pregnant and breastfeeding women from where you stay might want to use such things?

Ritah: It is acceptable the moment you know the purpose of it like if you get to know that it is used to prevent HIV then you will be allowed to use that medicine or the vaginal ring.

Pamela: People will be so happy about it when they get to know it because they are tired of the HIV infection. They will not be worried anymore.

Angel: Some women will decline to use it saying that, “the Whites have come to kill us just like it is for family planning methods.” When they give birth to babies who have some disabilities they say that it was due to family planning methods. So, I think some women will say that it is about Whites being trying to get involved in everything.

Samantha: To add on what she has said, some women will say that Whites have always tried to kill us; those are Whites’ tricks. So, some will accept and others will decline.

Annet: I think people will like it so much but won’t it increase promiscuity? Because they won’t be worried of anything.

I: It might increase promiscuity…

Annet: Yes.

I: Who else hasn’t told us anything?

Sarah: I think most people will accept [to use it] because we are many mothers and we are the ones who are in most need of staying healthy. I think women will accept to use it because they do not trust men.

**FGD Number:** FGD_41-F61

**Site:** Uganda

**Excerpt Range:** 64834-66006

I: Now, we have looked at the two products; the tablets and the vaginal ring. Which would be most preferred?

Angel: A vaginal ring.

I: You should also tell us the reason why women will like that product the most.

Angel: A vaginal ring because you insert it once for a month.

Ritah: Tablets because even men will be able to use them. They will be for both women and men.

I: Both women and men will be taking them…

Ritah: Yes, yet a man can’t insert a vaginal ring.

Annet: I think it is a vaginal ring because you insert it once and you do not have to keep inserting it every day.

Vanessa: I think it should be a vaginal ring because it doesn’t keep me worried of anything.

I: Which kind of worries are you talking about? How do

Vanessa: Having to remember to take medicines every day.

Sarah: I think it should be a vaginal ring because it is confidential.

Shanitah: Pills because I will be able to talk to my partner so that I do it he is in the know and we keep it to ourselves.

Samantha: I think it should be a vaginal ring because you won’t have to be worried whether you inserted it or not. And for any rumours that so and so found you taking ARVs will be eliminated.

**FGD Number:** FGD_41-F61

**Site:** Uganda

**Excerpt Range:** 66007-66753

I: Now, what level of protection would you want these products to provide? For condoms we are told that they provide a certain level of protection, what about these products, what level of protection would you prefer?

Samantha: I feel that at least they should be providing ninety nine percent.

Sarah: I would also say that it should provide ninety nine percent because when you decide to use it they should be able to use it.

Pamela: I think it should be ninety nine percent for the people to know that they can use it and avoid dying [getting infected with HIV].

I: How about the rest? Ritah, what level of protection do you think it should provide?

Ritah: Ninety nine percent.

I: Ninety nine percent. Angel?

Angel: That is what I also suggest.

**FGD Number:** FGD_41-F61

**Site:** Uganda

**Excerpt Range:** 67925-70122

I: What do you think is important in motivating a pregnant or breastfeeding woman to use a vaginal ring?

Shanitah: You will be so motivated to use it knowing that you will not get infected with HIV.

I: What might motivate pregnant and breastfeeding women to use a vaginal ring?

Pamela: Educating them about these products so that they get to know them.

I: How should they be educated?

Pamela: Through media like radios, televisions, so that for those who do not have a television can listen to a radio.

Samantha: I would also think that peer educators can go out in villages and educate women the same way we have been educated here might also motivate women.

R: I would also think that when women come for immunization they should also be educated about the ring in the same way we are educated about family planning methods.

I: When they go to hospitals…

R: For immunization or delivery.

I: How about those who might have the same fears like you do that “If I insert it for a whole month won’t it cause some side effects, won’t my baby be affected…”

Sarah: They should put some gifts to motivate women. They should give women gifts so that they listen to them and you get to explain very well.

I: How about if they raise fears just like you? What might be done so that women are able to use these products?

R: That will eventually disappear with time. It is just like where someone is told that she would be given a bottle of soda and she accepts to donate blood.

Angel: I would think that we first need to see someone who has used it and I think that might make us accept to use them.

Annet: I think if women are educated just like you have educated us they will like it.

Pamela: They should educate women in all the different languages and put in a lot of effort they will eventually use them.

Ritah: If they get to educate people over the radios and televisions they will use them because she would like to prevent HIV. If you educate them that in case one inserts a vaginal ring it prevent HIV, then, anyone who wants to prevent HIV will use it.

Annet: Especially prostitutes; they will use it.

Angel: I would think that counsellors should go back to their communities and explain to people.

**FGD Number:** FGD_41-F61

**Site:** Uganda

**Excerpt Range:** 70123-73057

I: Now, we would like you to advise us on how we could recruit pregnant and breastfeeding women in future research to see how these products work?

Ritah: Teaching them about the benefits of the tablets and the vaginal ring.

Annet: Identifying one of them who is either pregnant or breastfeeding and is using one of the products to speak to them. Someone who is using it to educate them when they come for their antenatal.

I: What do the rest think?

R: Those who have used it should convince them.

I: Now, looking at the video we have just played for you, do you think it would convince a woman to participate in this research or there is something that should be added?

Aida: I think we need to add what we have just said that someone who has used it might motivate women.

I: Someone who has used it to speak to people…

Aida: To speak to people and explain what she is experiencing.

Samantha: I also think that the video is lacking but if we include someone who has used it showing exactly when she is inserting it but not these cartoons you have just showed us I would think someone cannot decline to use them.

I: A video showing someone who has used it.

Samantha: Exactly and how she is inserting it.

I: And how she is removing it?

Samantha: Removing it would not be a problem but how she is inserting it is most important.

Aida: It is just like when you come to a hospital for delivery and you have fears but when you see someone delivering it can encourage you.

R: Those who have used it should testify that they have used it and it can prevent HIV.

I: Anything else? Angel?

Angel: What I was suggesting is that they should show how a person who has used the ring or tablet looks like, like we see how the HIV infected looks like and how the one who is not infected looks like.

I: How should the video show the person who has used the vaginal ring?

Angel: They should let us see who has used it before because there are people who have used family planning methods and they either gained weight or lost weight, so we need to see someone who has used the vaginal ring and someone who has swallowed the tablets before.

Sarah: We need to see a couple whereby a man is infected with HIV and they have used it and the woman hasn’t been infected, and then people will use it.

I: They should bring a couple…

Sarah: Yes…where one is infected and the other is not.

I: Where one who is HIV negative takes tablets?

Sarah: Yes…whereby they engage in sex and the one who is HIV negative remains negative. That is when it will become easy for them to understand that those products are effective.

Pamela: They should come to our communities and tell the people.

Samantha: I have a witness who was raped by an HIV positive man and she was given that medicine and within a few weeks she was tested and found HIV negative. The man eventually died.

I: Is there anyone who wants to add on something before we end?

[Silent].

I: Is that all?

R: Yes.

**FGD Number:** FGD_41-F62

**Site:** Uganda

**Excerpt Range:** 46392-48270

I:Now, we are going to look at our products; a pill that is taken daily and the vaginal ring to prevent HIV. Now, you have had an opportunity to touch the pill and the vaginal ring. Now, let us first talk about the pill. What comes to your mind when you think about using a pill every day to prevent HIV during pregnancy?

[Sandra]:The first thing that comes to my mind are the side effects because if you take tablets every day by the end of the month you would have taken about thirty tablets yet you do not have HIV…So, I would first think of the side effects that come out of that.

[Sharon]:Taking that tablet every day during pregnancy helps to prevent HIV but you can forget to take it and have engage into sex with a man and when you test you find yourself infected with HIV.

[Agatha]:The first thing I think about is whether it won’t affect the baby because you have told us that we can take it during pregnancy but won’t it affect the baby and probably deliver when is disabled? The other question, if I miss taking a tablet, is it possible to take two tablets the following day?

[Carol]:The first thing I get is fear asking myself when I will have to stop taking that tablet, would it be after delivery or I have to take it until I die? The other question, won’t it cause side effects like other diseases that experience when we take family planning methods, diseases like pressure…?

[Sandra]:I also think that it might make people believe that it prevents entirely and they might start engaging into sex recklessly knowing that they will not be infected yet when you do not follow instructions well like if someone misses a day, anyway, what I mean is that it brings some sort of freedom which might not be good.

[Barbra]:I also want to add to what she said that whether it won’t cause side effects if taken daily.

I:That is what you are asking yourself?

[Barbra]:Yes.

**FGD Number:** FGD_41-F62

**Site:** Uganda

**Excerpt Range:** 48271-49604

[Marion]:Does someone have to continue taking it after she has delivered or not? Won’t those who have HIV also take it thinking that since it prevents it might also work to treat?

[Suzan]:Do those tablets have advantages and disadvantages and do they require someone to eat well like how HIV patients are told to do and whether you are not supposed to be worried? In case I missed a day, does that have an effect on me and do I have to take it on a specific time like how family planning pills are required?

[Esther]:What comes first to my mind, do those tablets have an expiry date? Because I might take it when it is already expired and I am contented yet it won’t work and I end up getting infected with HIV.

[Maureen]: My question is whether it is possible to take it when someone is not pregnant.

I:After delivery?

[Maureen]:Yes.

R:You said that we have to keep this information confidential…

I:Yes.

R:Does that mean that when we go back home we shouldn’t tell our husbands? The other question, we are going to start taking these tablets but won’t our partners get to know about it and they start engaging into sex with anyone [because they are taking oral PrEP]?

I:You have told us what would come to your minds when you are pregnant, would that be the same during breastfeeding?

Chorus:Yes.

R:It is the same.

R:It is the same.

**FGD Number:** FGD_41-F62

**Site:** Uganda

**Excerpt Range:** 49605-50147

I:What are you worried about this tablet the most?

[Carol]:What we are mostly worried of, when we come for family planning we experience challenges, sometimes you may bleed or you may forget to take the tablet and you get pregnant…such things. And the other thing, some people fear taking tablets every day…

I:When you use family planning methods…

[Carol]:You bleed, you get back pains…so many things.

I:So, what do you then say about this tablet?

[Carol]:We are just asking whether it will not reach a time and cause some side effects to us.

**FGD Number:** FGD_41-F62

**Site:** Uganda

**Excerpt Range:** 50148-50411

I:Anyone else with other fears?

[Esther]:My question is, what procedures are supposed to be followed?

I:When you talk about procedures what exactly do you mean?

[Esther]:The terms to be followed; if you do not take it every day or if you miss a day, what happens?

**FGD Number:** FGD_41-F62

**Site:** Uganda

**Excerpt Range:** 50412-51684

I:But do you think that women might use that tablet? Pregnant and breastfeeding women, will they use that tablet?

Chorus:Yes, they can use it.

I:Would you think that there are certain things that might stop them from using the Oral PrEP?

[Barbra]:I think that tablet might increase a woman’s appetite yet she doesn’t have what to eat and she is pregnant. She might also swallow it and it causes some side effects like dizziness and others…but if it doesn’t have side effects then women might be able to take it.

R:Why I think we might be able to take it, men claim that they do not have time, when you ask him to go for testing they will always say that they do not have time.

I:What might stop women from using that tablet?

[Carol]:Some women fear taking daily tablets…and the other thing, her partner might ask “Why are you taking that tablet?” he might think that “This woman got infected and she never told me.” You know you can’t hide something forever to a partner you stay with at home, he might get to know about it and it brings trouble to you. You might have hidden it from him because you do not want him to engage into sex with other women after knowing that you are taking ARV tablet to keep you safe but if you hide it that might also cause problems at home.

**FGD Number:** FGD_41-F62

**Site:** Uganda

**Excerpt Range:** 52023-52999

I:Now, at what month should a pregnant woman start taking Oral PrEP?

[Carol]:Since we do not know how they can affect us, I think we are supposed to get that advice from you. We can’t decide on that…

I:But we just want to get what you think.

[Carol]:When she has just gotten pregnant.

[Suzan]:I think when she has just gotten pregnant the baby is still so young, I would think that it is better to start taking it when at least the womb is about three months. I think tablets might affect it and end up with a miscarriage if you take them at one week or a month. By that time it is still so young.

I:What do the rest think?

R:When you have just gotten pregnant.

I:How about [Esther]?

[Esther]:I would after two months when the baby has grown otherwise during those early days pregnancies are so delicate.

I:What happens during those first days?

[Esther]:That is when a woman vomits a lot and tablets also have their side effects…I think it should be started after four months.

**FGD Number:** FGD_41-F62

**Site:** Uganda

**Excerpt Range:** 54042-54948

I:Now, let us look at using a vaginal ring while pregnant.. what comes to your mind first when you think about using the vaginal ring?

R:How clean is that vaginal ring? If it stays inserted for a full month, won’t that cause some infections?

[Agatha]:Now, when it gets to that time when I am going to deliver, do I remove it or still keep it inserted inside?

R:I might be using it when my partner is not aware, won’t he be able to feel it?

[Carol]:I still have the same issue I talked about that if men get to know about it, won’t they become promiscuous?

[Sharon]:I would like to know at what month should someone start inserting during pregnancy?

I:At what month would you like that to happen?

[Sharon]:I want to get your opinion and then I will be able to tell you.

I:And that is why I am asking “You as a pregnant woman, at what time would you like to insert it?

[Sharon]:When I have just got pregnant.

**FGD Number:** FGD_41-F62

**Site:** Uganda

**Excerpt Range:** 55083-56405

[Esther]:Let me add to [Barbra]’s contribution. This vaginal ring is going to stay inserted for a full month, we have seen how to insert it, but won’t I scratch myself while removing it and it causes some problems to me?

[Suzan]:I fear inserting it myself because I might insert it wrongly and it goes to a different part from where it is supposed to go. I feel I might not be able to insert it the way a health worker would have done it.

I:[Maureen], what do you have to say about this vaginal ring?

[Maureen]: All has been said.

[Carol]:What I want to add on, in case I inserted it, doesn’t it shift? There is a time when my family planning method shifted from where it had been inserted. Now, for this vaginal ring, you fold it and insert it but in case I am having sex with a man, can’t that vaginal ring move out?

I:That is the question you have…

[Carol]:Yes…and in case it fell out, do I have to reinsert it at that moment or…what happens?

I:Anyone with another question?

R:For a breastfeeding woman, remember it stays inserted for a month, you know she will go into her menstrual periods when it is still inserted, won’t the blood make her stay dirty?

I:Won’t the blood…

R:Maybe stick on the vaginal ring and a lady develops a bad odour?

I:That is the question you have…

R:Yes. Anyone with anything to say?

[Silent]

**FGD Number:** FGD_41-F62

**Site:** Uganda

**Excerpt Range:** 56407-57898

:Now that you pregnant and breastfeeding women have seen it; what worries do you have about it?

[Carol]:What we are worried about it that we have seen it has medicine in it, I know the medicine is for preventing HV but won’t that medicine affect our health?

I:That is the worry you have…

[Carol]:Yes.

I:What exactly are you worried about?

[Barbra]:I wonder whether it doesn’t have side effects…the other is that it is so hard and it is supposed to be inserted inside the vagina and how you can keep it clean when you remove it out and then reinsert it…or is it used once and you replace it with a new one?

[Suzan]:My question is about breastfeeding women, won’t it have some side effects like…you know some family planning methods affect breastfeeding mothers and they can’t have enough breast milk…so my question is, won’t breastfeeding mothers lose breast milk?

I:Anyone with something else? [Maureen], do you have anything to say?

[Maureen]: When I am inserting it, I can’t see there [the vagina] and you said that I have to make it in form of number eight, remember you can’t see, how will you know it has made that shape of eight?

I:So, if you can’t be able to see, what challenge might that bring?

[Maureen]: You will definitely get infected because you told us that when you are inserting it you have to make sure it is in shape of number eight yet when you are inserting it you can’t tell whether it is in shape of number eight. How will you tell that it is in shape of eight or not?

**FGD Number:** FGD_41-F62

**Site:** Uganda

**Excerpt Range:** 57938-59468

I:But would you like to use it? Let me start with [Sharon].

[Sharon]:My question is, do I need to first inform my partner about it or I can still use it when is not aware?

I:How would you like that to be?

[Sharon]:It is better for him to know that you are protecting yourself and there is something you are using…

I:Why?

[Sharon]:You might insert it and it moves from position and he gets to see it.

I:Will it cause problems when he sees it?

[Sharon]:Yes.

I:Would you like to use it during pregnancy? [Carol]?

[Carol]:On my side, since I trust my partner I might not use it but I feel pity for these young girls that maybe instead of using Oral PrEP they should use a vaginal ring…

I:Why?

[Carol]:It does not worry like tablets [No need to worry about having to remember to insert it daily] that you have to take every day. The other thing is what we talked about that when it comes to tablets her partner might ask her “Why are you taking those tablets?” but a vaginal ring might be simpler to use.

I:But would you use it?

R:I can use it if I have understood it so well.

I:Like what?

R:Like the side effects, infections that might come from being dirty…and the other thing, when both of us together with my partner have understood it so well…maybe I can use it but as for now I can’t.

I:[Barbra], what do you say?

[Barbra]:I wouldn’t want to use it because I do not know about its side effects. I want to use it when I have clearly understood everything well.

I:You want to use it when you have understood it well?

[Barbra]:Yes.

**FGD Number:** FGD_41-F62

**Site:** Uganda

**Excerpt Range:** 59469-60586

I:[Agatha], what do you have to say, can you use it?

[Agatha]:I can use it if I have understood clearly well that I will deliver well.

[Suzan]:What I want to add to what [Sharon] said that you need to first talk to your partner, I think you can talk to him and he gives you money to go buy it and he ends up engaging into sex the way he likes but then it gets to a point when you are tired of using it just the same way someone might get tired of using a family planning method and then when you stop using it he then comes to you when is already infected and you end up getting infected.

I:So, what is your suggestion on that?

[Suzan]:That we shouldn’t first tell them [partners]. You can use it without him knowing to avoid him from engaging into sex with other women.

I:Okay, [Esther]?

[Esther]:I would like to use a vaginal ring because it does not put you on pressure like tablets that you have not yet swallowed them. Sometimes you may have travelled for a function and you fear people to see you swallowing them but for the vaginal ring no one would tell.

I:But would other women want to use it?

R:Yes.

R:Yes.

**FGD Number:** FGD_41-F62

**Site:** Uganda

**Excerpt Range:** 60587-61216

I:Remember some of you said that you would have wanted to use it if you have understood everything around it like its effectiveness, side effects…Would you think that other women might also want to use it?

R:Yes.

I:What do you think might help other women to use it?

R:They might be helped by some of the views we have given; it gives them peace because people fear tablets because when they are seen by someone they might think that she is infected yet the vaginal ring can be kept as a secret but tablets can’t be kept as a secret and people might say “She is lying, she has HIV but she is saying that she wants to prevent it.”

**FGD Number:** FGD_41-F62

**Site:** Uganda

**Excerpt Range:** 61217-62119

I:But it is acceptable for a pregnant woman to insert something inside her vagina?

[Esther]:It is acceptable because it is about someone’s health; there is no problem if it is about someone’s health.

[Carol]:I think it is acceptable because sometimes you can get an infection when you are pregnant and you are given some tablets to insert inside a vagina. So, if it is medicine to help you and it is not going to affect you but it is about your health then you can do it.

I:Can others also do it?

[Carol]:It is possible.

I:Do you think women from where you stay might also accept to use these products; the Oral PrEP and the vaginal ring?

Chorus: It is possible.

I:But what do you think they might prefer?

R:Vaginal ring.

R:Vaginal ring.

R:Most women fear taking tablets.

I:They will prefer a vaginal ring?

Chorus:Yes.

I:That they fear taking tablets?

Chorus:Yes.

I:That is what we all say?

Chorus:Yes.

**FGD Number:** FGD_41-F62

**Site:** Uganda

**Excerpt Range:** 63809-66719

I:Now, as you were informed, we intend to launch a research study in future for pregnant and breastfeeding women. What advice do you have for us that will help us to recruit pregnant and breastfeeding women in a research study that involves use of those products? How should we do it?

[Carol]:I think you should do it the same way you have done it today. When you educate someone and you properly explain the advantages I think it will motivate them. Those women need to be educated because most women at home are not empowered enough, hiding things away from your partner usually end up in problems. Family planning methods in the beginning were used without a partner’s consent but men used to complain until health workers requested women to come with their husbands so that they get to know that you are using a family planning method. Some women work very closely with their partners and they might want their partners to know right from the beginning but some men are faithful and when they get to know that you are using those products it might land you into problems, your partner might start doubting you “Since you never told me it seems you have some dubious actions.”

I:Anyone else with a different idea?

[Barbra]:Some women might be so interested in this but they are not well informed. There is need to inform them about such activities [Focus Group Discussions] so that they get to know about it before time and I think most of them will like these products.

I:How should they get to know about these activities?

[Barbra]:You should go through their community leaders like counsellors and we as well who are here can inform others about such activities but we need to know when it is about one week or two weeks before. We can talk to them “There is this activity…”

R: I also wanted to give the same idea because we are here today but most women do not know about such activities [Focus Group Discussions].

I:How should we get to them?

R:Pass on the information, you can use radios, televisions, counsellors, LCs…we will also go and talk to them “There is a vaginal ring which does this and that…” so that when that time comes when you need participants people already know about it. The organization can get some mobilizers who can go out there and mobilize people telling them…they should get peer educators stationed at health facilities and at every place where necessary so that everyone gets the information.

R:I think it will not be so hard for women to enroll because the time when family planning methods had just come women used to talk a lot about them “These are so hard, they have so many disadvantages” but we ended up using them. I think even for this they will accept because its aim is to help them and I think it will not take long before everyone accepts, they should try to educate them. There was a lot of education around family planning methods until women got convinced.

**Zimbabwe:**

**FGD Number:** FGD_41-F43

**Site:** Zimbabwe

**Excerpt Range:** 32509-33184

I: As explained before, we are interested in getting your opinion about two different products that women can use for HIV prevention, daily oral PrEP tablets and the monthly vaginal ring. I would like now to show you a video of the two products [Video is played]. Okay, so that’s the video showing the two products, the ring and the pill. So these are the PrEP pills, I want you to see them. [Pills are passed around]. The PrEP pill is taken every day just like the family planning pills. This is the ring, you insert it once for the for the whole month. It’s not removed during sex, during menses or even when bathing.

Tau: So the male partner doesn’t feel the ring inside?

**FGD Number:** FGD_41-F43

**Site:** Zimbabwe

**Excerpt Range:** 33248-34214

Let’s start by talking about daily oral PrEP tablets. What is your first thought when you think about taking oral PrEP to prevent HIV while pregnant?

Tanya: As a pregnant mother, what first comes into my mind is the issue of side effects to me and the baby, because just like any other pill there are side effects. The other thing is, since my hormones are already tempered around with because of pregnancy, will the PrEP pill go down well with me?

Ropa: I will be very happy to take this pill to protect me from HIV since I have always taken the vitamin pills every day. In this case I will be protected from HIV so I will be happy.

Tsitsi: I will be happy to be protected since I don’t want to get HIV.

Tau: It’s okay because I and the baby will be safe.

Charlene: I think using these pills is better because we do not have anything to prevent HIV with. Maybe your husband might fail to understand you but if they do not have side effects that will be good for us.

**FGD Number:** FGD_41-F43

**Site:** Zimbabwe

**Excerpt Range:** 34215-35547

I: What about during breast feeding, what do we think about taking these pills when breast feeding?

Linda: I would appreciate their use because currently I am not using anything, so if I do not react or anything I would definitely like the idea of using them.

Charlene: During breastfeeding, you would not know if they will not affect the milk or cause side effects on the breastfeeding baby. So I think it’s a bit tricky using the PrEP pill when breastfeeding.

Tanya: In as much as I want to prevent myself from HIV, the other thing that comes into my mind is what is going to happen if they dry up my milk, yet I am supposed to do exclusive breast feeding in the first 6 months.

I: Okay, so as we are here right now, would we be interested in using this product to prevent HIV as pregnant and breast feeding women?

Maka: I would love to use it as long as it prevents from HIV because if I get HIV I am still going to take pills [ARVs], though I might encounter some challenges.

Charlene: I would be comfortable using it during pregnancy, but when breast feeding I would not know what it does to the baby.

I: Okay, What worries do you have about oral PrEP, about your baby’s health or your health as a mother when using the pill?

Tsitsi: We might fear reactions on me and the baby.

Mary: I fear that maybe it might affect my milk.

**FGD Number:** FGD_41-F43

**Site:** Zimbabwe

**Excerpt Range:** 35548-37194

I: Okay, looking at the pill as it is, what do you think about it?

Tau: (laughs).

Shami: It’s big.

I: Okay what do you think the size of the pill is going to affect?

Shami: Generally I and pills do not see eye to eye. I prefer injections to pills. Just looking at the pill, taking it will be a big challenge for me.

I: Why would it be a challenge?

Shami: I do not know, I just don’t like them.

Tau: I agree with what everyone is saying.

Linda: I am used to small pills so I do not know if I will be able to get used to this pill.

Tsitsi: When I look at it, it’s big indeed. Some people generally find it hard to swallow pills, so I think this one is too big.

Tanya: I think maybe if it can be made a bit smaller. I fear for my throat!

Tau: People do not appreciate, they are scared for their throat, than HIV, you do not have to fear for your throats, you strive to swallow it so that it works for you. (Participants laugh).

I: That is what we want to hear.

Vicky: Personally I am not impressed by the blue colour. If I were to take it around people, what will they say? Maybe if it was white, it would at least look like you are just taking your paracetamols.

I: So you are afraid of what people will say, what do you think they will say?

Vicky: If a person asks me why I am taking it, then I say, “To prevent HIV”. Someone might not understand that you are preventing. It will need someone who would have been explained to but explaining what will be going on - -.

I: What do you think they will think if they see you taking the pill?

Vicky: They will think that I am HIV positive.

Tau: Why would you bother when you know you are HIV negative?

**FGD Number:** FGD_41-F43

**Site:** Zimbabwe

**Excerpt Range:** 37195-37970

I: Okay, what would facilitate taking oral PrEP for P and BF women out there?

Tanya: I think outreach programs can teach people to understand more about the pill, for example like what was done when the male circumcision programme was introduced. The more outreach programs they did the more people got to understand and feel encouraged.

I: So how do you think taking PrEP will interfere with our everyday lives?

Vicky: Feeling hungry all the time.

I: So how does that interfere with your everyday life?

Vicky: I am used to my own time table, but now feeling hungry after every 30 minutes will just not be okay.

I: Okay others, Tau?

Tau: I think at times you will not be used to it, and also the size will make you think twice, and also you might forget taking them as well.

**FGD Number:** FGD_41-F43

**Site:** Zimbabwe

**Excerpt Range:** 37971-38521

I: What period of time during P or BF would it be most feasible to take oral PrEP?

Ropa: At conception because you would not know when you will be exposed to the risk of contracting HIV. So it’s best to just protect yourself right from the beginning so that the baby will be fine.

Tanya: I think you need to take the pill when you feel that there is no longer trust in your relationship because I might just take the pills when he in the actual fact is not even doing anything. So I think it would be logical to take it when I feel I am now at risk.

**FGD Number:** FGD_41-F43

**Site:** Zimbabwe

**Excerpt Range:** 38522-38891

I: Depending on the culture, it may be permitted or taboo to take bitter medicine while pregnant. How would this interfere with women in your community’s ability to take oral PrEP while pregnant?

Charlene: I think that the fact that this pill now exist it means the doctors approved that we can use it. So if they say we can use it, there is nothing bad I see on that.

**FGD Number:** FGD_41-F43

**Site:** Zimbabwe

**Excerpt Range:** 38892-39072

I: Okay, what about on breast feeding, how can taking PrEP affect breast feeding?

Linda: I think this pill was tested and seen that it is safe for us to take during breast feeding.

**FGD Number:** FGD_41-F43

**Site:** Zimbabwe

**Excerpt Range:** 39084-39414

what are the things that are prohibited when someone is pregnant or breast feeding that can affect the uptake of PrEP?

Maka: Can you come again?

I: What other local taboos or practices would make taking oral PrEP while pregnant difficult?

Maka: Alcohol consumption is prohibited, so maybe that can affect the working of the pill.

**FGD Number:** FGD_41-F43

**Site:** Zimbabwe

**Excerpt Range:** 39415-41330

I: Now let’s talk about the vaginal ring. What is your first thought when you think about using a vaginal ring to prevent HIV while pregnant?

Charlene: Won’t the male partner feel the ring during sex? Because one would think you are up to something, so I want to know if it won’t disturb him during sex.

I: Okay others, Charlene thinks the male partner might feel it?

Tau: You cannot just take the ring and insert it without telling him what you want to do? You first have to tell him in case he feels it.

Charlene: I want to support that, you might insert it and he will know of it and he would think that you do not trust him. It’s just the same on HIV testing, he might think I am accusing him of being unfaithful. So I think just inserting it quietly is better because if you tell him he will say you do not trust him yet you just wanted to protect yourself.

Tsitsi: I think it’s okay to use the ring, but you need to first of all talk about it. You tell him that there are a lot of ways of HIV can be transmitted hence the need to protect herself.

I: So why do you say the idea of using the ring to prevent HIV during pregnancy is good?Tsitsi: It is good because ring cannot be forgotten like pills. It can also be used discreetly in case partner does not agree to ring use.

I: Okay, others what do you think?

Linda: I think it’s good because you can visit somewhere and fail to come back and sleep over. You will not have to worry because your ring will be in place unlike when you are taking the pill.

Tanya: My fear on accepting the ring is the fact that it is a foreign body, I wasn’t born with it. So I start to think, “What if it falls when I am around people and won’t it cause pain”. Of course the video mentioned that you won’t feel the ring and that it is comfortable, but I think you will not feel it later on. At first you might feel it because it’s something that you did not have all along.

**FGD Number:** FGD_41-F43

**Site:** Zimbabwe

**Excerpt Range:** 41600-42343

I: So if you were to be given the ring today to use to prevent HIV during pregnancy and breast feeding, would you be interested in using the ring?

Tsitsi: Personally I think the ring is better. These pills are tricky, if your family members start to see you taking these pills every day they will not understand you. Even when you try explaining that it is to prevent HIV some of our parents might take long to understand or might even think that you are on ART.

I: What do you think would make the ring difficult to use?

Linda: I think the ring is too hard, (baby cries).

Shami: I think other people might not insert it correctly then it affects things in there.

Maka: I think the man might equate the ring to a condom and refuse to use it.

**FGD Number:** FGD_41-F43

**Site:** Zimbabwe

**Excerpt Range:** 42344-42537

I: Okay, what can encourage and motivate people to use this ring when pregnant and breast feeding to prevent HIV?

Mary: Personally, what would push me is the need to prevent my child from HIV.

**FGD Number:** FGD_41-F43

**Site:** Zimbabwe

**Excerpt Range:** 42547-43152

Depending on the culture, it may be permitted or taboo to insert things in the vagina while pregnant. How would this interfere with women in your community’s ability to use the vaginal ring while pregnant?

Maka: I think the ring is good because it is something that was examined and tested by scientists and doctors unlike herbs that are just taken from and you are told to insert. The ring is safer.

Tsitsi: I wanted to say the same thing. These herbs are tricky because you do not know if you will react, but these rings would have been examined by doctors and they know that they have no side effects.

**FGD Number:** FGD_41-F43

**Site:** Zimbabwe

**Excerpt Range:** 43153-46295

I: So would the pregnant and breast women in the society like to use products like these in HIV prevention?

Maka: My view is that they will use them because they are very worried. Men are giving them a hard time and they wish if they could have something to protect them and some actually say if only the virus would only infect the one who goes out to acquire it. So I think that they would be really happy.

Ropa: I think what’s important is learning and teaching about these products, because people can value the advantages over the disadvantages of using these products. Personally I think they are good products.

I: Okay, so comparing the two products, which product would women like the most?

Charlene: I think they will prefer the ring because some women forget a lot especially if they are not used to taking pills. So it’s good if the ring just stays in situ then you remember at the end of the month to replace.

Maka: I think that they will prefer the pill because they are just the same as a family planning pills and generally contraceptive pills are the most used method of contraception. Inserting things inside, women might be reluctant.

I: Why would they be reluctant?

Maka: They have the fear that it might move because there are stories that some contraception that are inserted under the skin like Jadelle moved, so people might just want the pill.

Mary: I think they will prefer the pill because it will not affect anything. With the ring I have fear that if it is pushed it will go further into the vagina or that maybe it might fall in front of people.

Tau: I am scared that during sex the man can push it further or come out with the ring stuck on his penis. (All participants laugh).

I: Why are you afraid that it might be pushed further?

Tau: It might get inside the cervix.

I: Do you think P & BF women would be able to use these products successfully?

Mary: I think that if they are taught they will be able to use them successfully because women we are afraid of getting infected with HIV. There is nothing painful like being infected with HIV when you know you were you were very faithful. So I think if they are taught like I have been taught here they will be able to use them appropriately.

Tanya: I think when you are running, your speed is determined by what is chasing you. So product use will be determined by a person’s situation.

I: Okay can you simplify?

Tanya: Marriage is not all about this person has cheated or done that because a lot of people are doing that. So if you know that you or your partner has multiple sexual partners, then you will need to take it. But if you feel there is something [Some unfaithfulness] but there is no evidence to prove what you are thinking, you might have that negligence or feel reluctant to use the products successfully.

I: So in other words you are saying the ability of using these products successfully depends on the person’s perception of risk?

Tanya: Exactly.

Tsitsi: I want to add that if you know that your partner is not faithful then you will rely on the products for protection, but if he is not then you might as well forget taking the pills. .

**FGD Number:** FGD_41-F43

**Site:** Zimbabwe

**Excerpt Range:** 48057-49232

I: What can encourage or motivate women who are pregnant or breast feeding to use these products?

Tanya: Unfaithfulness in marriage. If the woman knows that she is promiscuous, it will motivate her to use them.

I: What can be done to encourage or motivate the use of these products?

Tanya: I think outreach programs that can teach people, or doing short stories about the products on TVs and radios.

I: What can be done to remove the fears that might affect the use of the pill or ring?

Maka: If there are other countries who are already using these rings or pills, they can show case on TVs giving testimonies about these products’ use. That can help.

I: What can we change or add so people who have never heard of oral PrEP or the vaginal ring for HIV prevention understand the products?

Tanya: For the products to be understood well, people already using them can come and do adverts on the products than using cartoons with a voice in the background.

Tsitsi: I want to support that. If there are people who are using these products, we need those to come and share their testimonies in the same manner adverts about support groups for people living with HIV are done.

**FGD Number:** FGD_41-F43

**Site:** Zimbabwe

**Excerpt Range:** 49242-50433

What suggestions do you have on how we can recruit pregnant and breastfeeding women for a future study in which they will use these products?

Maka: I think you should really explain to them that you will stand with them 100% throughout the study, even in challenges. The first thing is to emphasise on the fact that there are no side effects, and in the event that there are there, then you will be with them all the way.

Tanya: For pregnant women, firstly promise free delivery and checkups, this will make the person want to join. Then secondly, the biggest thing that everyone wants, money! It motivates a lot of people.

I: So these women who are breastfeeding and pregnant, where can we find them?

Ropa: Most of them we find them in our communities and the churches we go to.

I: Okay how can we find them there?

Charlene: I think as we have been taught like this, we can spread the word, and you guys you can keep on advertising so that they can come. Right now we are going to teach them about the advantages and that will make them want to come.

I: Others?

Mary: I think when we come to the baby clinic, the nurses can teach about it like 10 minutes, think they can be found there.

**FGD Number:** FGD_41-F41

**Site:** Zimbabwe

**Excerpt Range:** 39607-41284

I: So, wwould oral PrEP be something you would be interested in using while P or BF, Jane?

Jane: I think it is something good for us to use but I am not sure whether this this thing is 100 percentage effective so that I do not get infected, will I be well protected. That is where we are not clear whether we will be 100 percent protected, 100 percent safe or there are some side effects, or there are some risks that can cause the baby to get sick.

I: Ok.

Jane: That’s where it is not clear.

I: Alright. So you are worried because you do not know whether it is 100 percent--.

Jane: Whether this thing is 100 percent safe or not.

I: And the issue of side effects.

Jane: Yes.

I: Ok. It is fine. That is what comes into Jane’s mind, what about others? Ok, considering what Jane mentioned, concerns about whether it’s 100 percent effective and fear of side effects, what type of side effects are you afraid of about PrEP?

Jane: I am not sure whether PrEP does not have the same side effects with anti-retroviral [ARV] pills.

I: Ok.

Jane: We see people reacting differently. Some have their faces enlarged or get puffed that shows that this person is taking pills [ARVs]. There is a lot that happens to people who take ARVs.

I: Alright.

Jane: That is what we are worried about.

Tanatswa: We are also worried because there are some drugs that people with certain conditions like heart problems are not supposed to take. I might not be HIV positive but might have another condition. That is what I am worried about though this is a very good thing.

I: Uhm.

Tanatswa: Because we would have managed to protect our children together with us as well.

I: Ok.

Tanatswa: Yes.

**FGD Number:** FGD_41-F41

**Site:** Zimbabwe

**Excerpt Range:** 41285-42805

I: Ok, what about others? Ok, so what about the tablet, would it be hard to take it every day? It was said for it to be effective in preventing HIV it has to be taken everyday right?

All: Uhm.

I: So what can make it difficult for women to take it every day, Tendai?

Tendai: I don’t see anything bad because they are just like family planning pills. I have always taken them every day so it will not be difficult for me to take this pill knowing that it is protecting me. That is what I can say.

I: Uhm. Jane?

Jane: I don’t think it will be difficult for us to take this pill, or it will not be difficult for me because I use oral contraceptive pills. But there are other women who forget to take their family planning pills.

I: Uhm.

Jane: But now I am not sure that if this one is forgotten, will it not cause side effects? We hear with ARVs you are not supposed to skip.

I: Uhm.

Jane: So we are not sure that if this one is forgotten, will there not be any problems.

I: Ok. Someone else, what can make taking this pill everyday difficult for women, Sarah?

Sarah: I don’t see anything bad. It can be taken every day but there are some who forget. They are the ones who will have problems. Such women would need to use things like the ring, there is nowhere they can forget it because it will always be inserted.

I: Uhm.

Sarah: Yes.

I: TK?

TK: I wanted to say what Sarah has just said that if you know that you forget, you just choose to use the ring. To some of us who do not forget-- [Other participants laugh].

**FGD Number:** FGD_41-F41

**Site:** Zimbabwe

**Excerpt Range:** 43149-43722

Tanatswa: I just wanted to support what Jane said that us women, if we really understand how important this pill is to our babies and to us as well, we will be able to accept it and take it every day.

I: Alright. How can women come to this understanding, what can be done for women to understand this, Tanatswa?

Tanatswa: I think if there can be community educators, they can be health workers or--, who can teach since these are new things that are coming in right.

I: Uhm.

Tanatswa: Teaching so that woman do understand. I think most women will be interested in it.

**FGD Number:** FGD_41-F41

**Site:** Zimbabwe

**Excerpt Range:** 43723-44057

I: Ok. What period of time during P or BF would it be most feasible to take oral PrEP, Sarah?

Sarah: This has no appropriate time. You should always be protected.

I: Ok.

Sarah: HIV has no time because you can get infected before the time you are anticipating to start taking the pills.

I: Ok.

Sarah: So it is good to stay protected.

**FGD Number:** FGD_41-F41

**Site:** Zimbabwe

**Excerpt Range:** 44058-45203

I: Uhm. Anyone who wants to add? It is fine. How would the tablet interfere with your daily life, say you are now taking PrEP, how would it interfere with your daily life, things like your social life, sex life, how would it interfere, Nyasha?

Nyasha: I don’t think it will interfere with anything.

I: Uhm, why?

Nyasha: Because it is just like other pills that we have always been taking on a daily basis.

Tendai: It is good, I am not being negative.

I: Yes.

Tendai: But sometimes like what the family planning pills do, you can take and have side effects like a headache. In that case can you come back to report your challenges and have them changed.

I: Jane?

Jane: Since we do not know how this pill works, it might be having some side effects like what Tendai is saying. For example, the family planning pill, I have 8 years taking them. Every morning I would wake up vomiting like someone who is pregnant. So when you take this pill for a long time, won’t it cause some things in your life?

I: Uhm.

Jane: Such things can now affect your daily life because in the morning you can fail to do your daily chores feeling dizzy or something.

**FGD Number:** FGD_41-F41

**Site:** Zimbabwe

**Excerpt Range:** 45219-46258

Depending on the culture, it may be permitted or taboo to take bitter medicine while pregnant. How would this interfere with women in your community’s ability to take oral PrEP while pregnant?

TK: Can I have the question again?

I: Depending on culture right, it may be permitted or taboo to take bitter medicine when you are what,

All: When you are pregnant.

I: When you are pregnant right. Because of that culture, how can it interfere with the community’s ability to take oral PrEP while pregnant when people know that they are not supposed to take bitter medicines while pregnant?

Tendai: As something that was tested and seen that we can use it, I do not think it will be difficult when we are taught and we understand. I do not foresee any problems for us to use it because it was already tested by doctors and they saw it fit for us to use it. So we can accept it.

I: It is ok. Memory?

Memory: I do not think there is anything bad for us to use it because it was tested worldwide and was seen that it works.

I: Ok.

Memory: Uhm.

**FGD Number:** FGD_41-F41

**Site:** Zimbabwe

**Excerpt Range:** 47000-48654

I: What is your first thought when you think about using a vaginal ring to prevent HIV while pregnant, you have seen it?

All: [Laugh].

I: You have laughed. Jane, using it during pregnancy, to prevent HIV?

Jane: Fear comes first. That thing [Ring] is too big. [I and other participants laugh].

I: Alright. The issue of size, yes.

Jane: Plus the thickness, won’t that thing be painful when inside [The vagina]?

I: Ok. So you are afraid that it is too big?

Jane: I just started thinking that this thing is big.

I: So what do you think the big size will do?

Jane: Won’t it enlarge the vagina?

I: Ok.

Jane: Isn’t it will be placed inside right?

I: Yes.

Jane: With that big size now if inserted inside what is it going to be like in there [In the vagina]?

I: What are you afraid of if the vagina enlarges during pregnancy?

Jane: [Laughs]. Already as I progress in pregnancy the vagina will be enlarging and with this [Ring] now, what will the sex partners say. They will not enjoy sex.

I: So you are afraid that it will affect sex?

Jane: I doubt its acceptability by sexual partners.

I: They will not accept it?

Jane: These are just my views.

I: That is what we want.

Jane: Men might fail to accept this thing. I see as if it is big. That is how I see it.

I: That is what we want. It is fine. What about others, TK?

TK: Aah, this thing [Ring] is big.

I: Uhm.

TK: As I am seeing it.

I: Ok. So what does the big size affect?

TK: Aah, I am just afraid of pain and won’t sexual partners feel it?

I: Ok. So you are afraid of pain and that sexual partners might feel it?

TK: I am afraid that sexual partners might feel it [Other participant s laugh].

**FGD Number:** FGD_41-F41

**Site:** Zimbabwe

**Excerpt Range:** 50842-51918

I: You also mentioned that you are afraid of pain and that partner might feel the ring, what other worries or concerns do you have concerning ring use during pregnancy and breastfeeding?

Nyasha: We want to know whether this thing [Ring] will not move from its position while inside the vagina.

I: Alright. What are your fears about that?

Nyasha: Shifting might affect ring effectiveness.

I: Alright. Jane?

Jane: I heard that it contains the drug. Will it not cause infection or things like cancer on the site it will be placed?

I: It is fine. Would the vaginal ring be something you would be interested in using to prevent HIV during P or BF?

Jane: Since HIV is a problem in the world, nothing can be difficult for this ring to be used except the fears of side effects people might have. Otherwise every person needs a method to prevent HIV especially when you are aware that you don’t have HIV. You will be interested in using anything that can prevent HIV but we will only be worried about the possible side effects. You will be afraid of developing things like cancer.

**FGD Number:** FGD_41-F41

**Site:** Zimbabwe

**Excerpt Range:** 52255-52767

I: It is fine. What would facilitate using the vaginal ring for P and BF women, maybe comparing it with other HIV prevention products?

Tanatswa: From the way I see the ring.

I: Uhm.

Tanatswa: We said the ring lasts for a month right?

I: Uhm.

Tanatswa: It is different from a pill because a pill can be easily forgotten and it is taken daily. As for condoms, men do not like them. So if you have the ring, you know that it is for the whole month. That is its advantage.

Jane: I wanted to say the same point.

**FGD Number:** FGD_41-F41

**Site:** Zimbabwe

**Excerpt Range:** 54500-54745

Jane: Women with uterus problems can have challenges using this ring.

I: Uhm.

Jane: I have a problem with my uterus and when I get cold it becomes painful. So now when there is something like the ring inserted closer to it, won’t it cause pain?

**FGD Number:** FGD_41-F41

**Site:** Zimbabwe

**Excerpt Range:** 54746-55944

I: Ok. What would facilitate using the vaginal ring for P and BF women?

Tanatswa: I don’t know whether it can be felt by sexual partners, but the ring is good in that it is different from pills that can be easily seen.

I: Ok.

Tanatswa: Yes. People will say, “Ah, what are these pills for?” and they can actually say that you have HIV. The ring is discreet. If husbands are not cooperative, women can just use it as long as it will not be felt during sex.

I: Alright.

Tanatswa: You just use it in privacy. That is the advantage of this ring.

I: So you are saying it is private?

Tanatswa: Yes.

Tendai: I support Tanatswa because for sure the husband may fail to accept it when you are worried about your health.

I: Uhm.

Tendai: So you will see that using the ring is good. I will have it inserted in a private place [in the vagina] so that my life will be preserved. He can say that, “You don’t trust me,” but you wouldn’t know what he will be doing out there. So, I will be worried about my life and the baby that will be in my womb and even during breastfeeding. The ring is good. You can just use it privately if you fail to agree on ring use with partner. So, it is something that is good.

**FGD Number:** FGD_41-F41

**Site:** Zimbabwe

**Excerpt Range:** 55945-56322

I: It is fine. Say you are using the ring, how would it interfere with your daily life? Besides the issue of partner feeling the ring during sex as said earlier on by Tendai, how can the ring interfere with your daily life?

Tendai: I think we need to use the ring first so that we can see its disadvantages. That is when you can learn whether the ring is good for you or not.

**FGD Number:** FGD_41-F41

**Site:** Zimbabwe

**Excerpt Range:** 56323-57824

I: Ok. What about by just merely looking at it, what do you think...Ok. Depending on the culture, it may be permitted or taboo to insert things in the vagina while pregnant. How would this interfere with women in your community’s ability to use the vaginal ring while pregnant?

Tendai: I just want to ask, maybe I am getting lost. Say you have this thing [Ring] inserted right.

I: Uhm.

Tendai: Won’t dirt accumulate where it will be placed or when the dirt accumulates, are we going to have our uterus cleaned? Where exactly will the ring be placed? Maybe I don’t know, where will it be placed, will the ring be clean where it will be placed?

I: What do you think will be happening to the ring when placed up the vagina?

Tendai: That is what I want to know. When releasing the drug for the prevention of HIV, will it also be releasing the dirty that would have accumulated on it or the dirty will just be kept there?

I: Uhm, I want to hear what you think, or what are your fears concerning that, feel free?

Jane: I think infection develops where the ring will be placed and that is where most people lose interest in using the ring because when infection develops it can cause uterus diseases.

I: Ok.

Jane: So I think the interest of using the ring in women can decrease because of that.

I: Uhm. Tendai you said you are afraid that the ring will accumulate dirt. What do you think will get affected?

Tendai: That is what Jane is saying that maybe it will affect the uterus.

I: Uhm.

Tendai: Yes.

**FGD Number:** FGD_41-F41

**Site:** Zimbabwe

**Excerpt Range:** 57825-59795

I: It’s ok. Others, what do you think? I was asking about the norms and taboos in your culture about inserting things during pregnancy. How can they affect ring use? You mentioned that at the clinic you are told not to insert things into the vagina because that is what causes cervical cancer. So how can such beliefs affect ring use which is supposed to be inserted inside the vagina?

Tendai: I am taking you back again.

I: Yes.

Tendai: The ring was said [from the video] it is supposed to prevent all the diseases that can come through sexual partners like sexually transmitted infections [STIs] and HIV. So the ring will be fighting all that.

I: Ok.

Tendai: Yes.

I: E-eh, this ring was meant to prevent HIV only.

Tendai: Uhm.

I: I want that to be clear. It is aimed at preventing HIV only. So the teachings that we are not supposed to insert things into the vagina and the ring that is supposed to be inserted into the vagina, how can that be solved since these two are clashing. How can that be solved, TK?

TK: Can you please come again?

Tendai: That is where our request comes to you researchers to say, “Will the ring not cause other diseases like cancer and so forth even though it can prevent HIV”.

I: Uhm.

Tendai: Like what Jane said, the ring is likely to cause problems where it is placed inside the body?

I: In other words you are saying if there could be teachings so that people will be able to understand better?

Tendai: So that we can accept it.

I: So how do you want the lessons to get to women?

Jane: I think such teaching sessions can be done at antenatal clinics. People from different areas will come and meet with such teachings. This will be very helpful since most women are found there.

I: It’s ok.

Note taker: I wanted to add that we are doing MTN 041, knowing that these product are able to prevent from HIV. The ring itself and the pill. So the aim is to see whether pregnant and breastfeeding women will be able to use them.

**FGD Number:** FGD_41-F41

**Site:** Zimbabwe

**Excerpt Range:** 59796-60175

Jane: We want to use them but we need to be taught, to have adequate teachings especially about the ring because our fear is whether there will not be many side effects from the ring. The pill is not complicated, maybe it might cause some side effects but with the ring we are afraid that maybe partners might feel it, and because of the big size, there is a lot we can think of.

**FGD Number:** FGD_41-F41

**Site:** Zimbabwe

**Excerpt Range:** 60617-61122

I: Uhm. It is fine. So from your own perspective right, will woman from your community be interested in using these products, the ring and PrEP during pregnancy and breastfeeding?

Tanatswa: I think women will be very happy to use the products.

I: Uhm.

Tanatswa: Because they are very good to us, you will be able to protect your baby from HIV or even yourself during pregnancy when you are using the ring and the pill. So it is something very good to us, women will accept them.

I: Ok.

Tanatswa: Uhm.

**FGD Number:** FGD_41-F41

**Site:** Zimbabwe

**Excerpt Range:** 61123-62408

I: Comparing the ring and the pill, what would women prefer to use between these two products, ring or pill during pregnancy and breastfeeding?

Sarah: Women will prefer the ring. Ring is easy to use than the pill.

I: Ok. Why do you say the ring is easier to use than the pill?

Sarah: The pill can be easily forgotten and the big size, one can find it difficult to swallow and generally some people do not like pills.

I: Uhm.

Sarah: With the ring once inserted you will not have any problems. Once inserted one will wait till end of the month.

I: Ok. Others?

Tendai: Most women in the community will prefer using the ring because if I go and tell my husband about the ring he might not accept it when I actually see the need to use it. It is important in protecting my life. So I see that it is good for me because I will just have the ring inserted quietly because I will be protecting my health.

I: Ok.

Tendai: Most women will like the ring, it is very important to most women.

Tanatswa: I support what Tendai is saying about the ring. Most women will like using it.

I: Uhm.

Tanatswa: Because at times men do not understand. If you try to tell them that this is a pill they don’t understand because they do not have adequate knowledge. They will think that you are lying.

**FGD Number:** FGD_41-F41

**Site:** Zimbabwe

**Excerpt Range:** 62409-63265

I: Others with different views? What about comparing the condom and these two products, the ring and the pill. What would most women prefer?

Memory: They will prefer the condom. Ring might cause pain during insertion and the pill might be forgotten.

I: Ok. What is the advantage of using the condom?

Memory: A condom is good because it is used with each sexual act.

Tendai: The products are good compared to the condom because I will be having sex without a condom. That increases sexual pleasure for me and my husband. [Other participants laugh].

Tanatswa: Comparing the products with the condom, condoms can burst.

I: Ok.

Tanatswa: The way I see it, comparing the two products and the condom, a condom is not very safe because it can burst and you will infect the baby just because the condom had a burst. So I see that these products are good.

**FGD Number:** FGD_41-F41

**Site:** Zimbabwe

**Excerpt Range:** 64490-65587

I: Ok. So what can we change or add so people who have never heard of oral PrEP or the vaginal ring for HIV prevention understand the products?

Jane: I don’t think there is anything that needs to be changed. But what can be done is to increase people awareness of the products.

I: Uhm.

Jane: Telling them about these two products highlighting the possible side effects the products can have. That is the information that can be made available to pregnant and breastfeeding women as well.

I: Ok, to add on to what Jane said, what kind of messages would appeal to P and BF women in your community?

Jane: Messages that people like?

I: That will make women like the products, or that will make them accept these products?

Jane: The fact that it prevents HIV will make people like and accept the products. So it is adequate enough teaching people that the products prevent HIV and they help your baby not to get infected by HIV during pregnancy and breastfeeding.

I: Ok. What can be done to motivate women to like the products?

Tendai: Teaching and encouraging each other to accept the products.

**CODES:** PILL (T), BARRIERS (T), ACCEPTABILITY (F)

**TRANSCRIPTS:** ALL P&BF WOMEN FGDs

**MALAWI:**

**FGD Number:** FGD_41-F84

**Site:** Malawi

**Excerpt Range:** 63308-63810

I: So, the way we have seen the tablet, what is it about the pill that would make the women not to take it daily?

[Triza]: The problem with the pill is being forgetful… if we forget to take the contraceptive pills…we do remember late, waking up past something…to take the pill…[laughing]…so the pill, I think for those that can remember they can take it but not for those that are forgetful like me…I can’t try it…[giggling]…it is better I opt for the ring because I will just stay with it…[laughing]

**FGD Number:** FGD_41-F84

**Site:** Malawi

**Excerpt Range:** 64320-65312

I: So our friends have shared with us what would encourage them to take this pill…yes Monica, do you have different views from what our friends have shared?

[Monica]: [sighing] …no everything is okay.

I: What would encourage you to take the drug every day?

[Monica]: For me I would opt for the ring.

I: You would opt for the ring…would you share with us why you have chosen the ring and not the pill…that is your right…but we just want to know what would stop you from taking the pill every day?

[Monica]: Pills are bitter.

I: Oh bitterness…so that would make you choose the ring?

[Monica]: Yeah… it thought the ring remains inserted for a month?

I: Yes, for a month…so what is the main reason you would choose the ring?

[Monica]: The ring has pleased me and not the pills.

I: How has it pleased you?...please clarify on that.

[Monica]: It has pleased me that the ring can remain for a month.

I: A month still inserted?

[Monica]: Unlike taking the pill daily…I can easily forget.

**FGD Number:** FGD_41-F81

**Site:** Malawi

**Excerpt Range:** 29587-30276

I: What kind of worries can the women have in taking these tablets daily and why can they have those worries? [Baby crying irritably though softly].

Lucy: Yes worries are inevitable. Taking these tablets to protect yourself from HIV while you are pregnant can be worrisome. This can be so, because you don't know how the drugs will work in your body while you are pregnant. They may come with so much strength that may lead to fatigue, or can even destroy the baby you are expecting. So, yes it is good that the drugs will protect from HIV, but they may bring some undesirable side effects while you are pregnant; as it is said that when one is pregnant, she should not be taking drugs.

**FGD Number:** FGD_41-F81

**Site:** Malawi

**Excerpt Range:** 33128-34423

I: Culturally, how acceptable or not acceptable is it to take bitter medication when a woman is pregnant and how would taking this medication disturb women in our communities from taking the tablet that prevent HIV acquisition?

NT: In other words, let us go back to where we have come from. Let us look closely how our cultural beliefs are. Our cultural beliefs on how we can take medications, and the cultural beliefs on taking bitter medications. And on the same culture, what does it say on taking bitter medications and how about taking the bitter medications when you are pregnant? Is it acceptable or not according to the cultural beliefs that we know from the communities we are coming from? That is why we want to hear different views and that is the question she is asking.

Alice: Taking bitter medication can cause an abortion.

I: Taking bitter medication can cause an abortion?

Alice: Umm.

I: So, that is what cannot be acceptable in taking bitter drugs?

Alice: Umm.

I: Are there any more additions…any more comment?...

Patricia: Some say taking drugs anyhow can make you have a baby that behaves abnormally or physically abnormal.

I: There is a risk that when a pregnant woman is taking drugs it can destroy the baby she is expecting eh?

Patricia: umm [Yes].

**FGD Number:** FGD_41-F81

**Site:** Malawi

**Excerpt Range:** 34475-35714

What do you think are the things that can prohibit from taking these drugs, these tablets, from practises and cultural beliefs that are there in our communities? What cultural beliefs can prohibit a woman from taking the tablets that can protect them from HIV the time they are pregnant?

Favour: What can prohibit women from taking these tablets is that, in our communities there a people who say a lot of things, they may say a lot of discouraging things about the PrEP tablets. But as an individual, you just have to see how things are going now in this world and listen to what the hospital personnel are saying and also how our health is these days, so that you should use these tablets.

I: What kinds of discouraging things?

Favour: It may happen that people can say lots of things about these tablets that it can make you think of not using them.

I: I want you to tell me the exact discouraging words that can be said.

Favour: They may say the tablets are bad and may destroy you inside.

I: Alright, Is there any addition?

Ethel: There are some who believe in religion. They may go collect the tablets then just dump them home and they say you should just be praying and the Lord is going to deal with the infection.

**FGD Number:** FGD_41-F81

**Site:** Malawi

**Excerpt Range:** 43803-45211

So, beside the doctor, who can get involved in making decisions on whether a woman can use the products we have discussed, ring and oral tablets when she is pregnant or breastfeeding. Besides the doctor explaining to you the importance of ring and tablets that should protect, who else do you think can encourage us in the communities?

Esther: Friends we chat with can encourage us.

I: When you say friends, do you mean males, those we sleep with or...?

Esther: No, just mere friends.

I: Your fellow women, the ones you chat with, eh? Is there anybody else we think can encourage us, or with whom we can encourage each other to be using these products in order to protect ourselves from acquiring HIV?

Favour: The chiefs are also supposed to take part in our communities in encouraging people to take these products.

I: Thank you very much. Are there some more people whom we think can encourage us, we have talked about chiefs, friends.

Kheliwe: Health Surveillance Assistants.

I: Health Surveillance Assistants who are working in our communities eh?

Kheliwe: Yes.

I: What about our mothers and mother- in- laws?

Lucy: It will depend on what kind of mothers they are. There are some mothers that like traditional herbs so much, so such type may not encourage you to take these products. But there are others who believe that traditional herbs are not good, they the type that can encourage you.

**SOUTH AFRICA:**

**FGD Number:** FGD_41-F24

**Site:** South Africa

**Excerpt Range:** 77823-80703

I: Okay as explained before we are interested in getting your opinions about the two different products that women can use for HIV prevention, the daily oral PrEP tablets and the monthly vaginal ring. Let’s start by talking about daily oral PrEP tablet, what is your first thought when you think about taking oral PrEP to prevent HIV while pregnant?

Nonhlanhla: I think it’s alright to prevent while you are pregnant and after…The pill is alright.

I: After when?

[Nonhlanhla]: Like after you have found out that you are pregnant, and you have tested and know about your status then its fine, the pill is alright to take.

I: Why do you say the pill is alright?

[Nonhlanhla]: I fear the other one… [The vaginal ring].

I: What are you scared of?

[Nonhlanhla]: What is it…?

I: The ring…?

[Nonhlanhla]: Yes, it scares me, so I prefer the tablets.

I: Others what do you think of taking the pills to prevent HIV while pregnant?

[Makhosi]: I also prefer the pill I am also scared of it [the ring] what if…Isn’t that the sizes of the vagina are not the same, what if you can’t get it in or while inside it doesn’t come out.

I: Why do you say you prefer tablets, I want us to talk about the pills now and we will then discuss the ring afterwards? I want to talk about whatever things that may make you prefer to use the tablets or not prefer the vaginal ring, let’s talk about those?

[Grey]: Okay earlier when we were sitting on that side and the lady who was reading the informed consent for us she said these pills can help for both oral and vaginal sex because there are those who prefer oral sex to vaginal sex. Then you can help for both oral and vaginal sex.

I: How can they help with that?

[Grey]: They prevent infections through both vaginal and oral sex that is what she said to us. I think it is best to take the pills for when your partner wants to do oral sex they will help you because the ring protects only the vagina.

I: Okay we have heard [Grey], what do others say about taking daily oral PrEP to prevent HIV? Let’s talk so that we can finish.

[Pink]: We agree that it is better to take the pills to prevent…

I: While pregnant…

[Pink]: While and after because when you are pregnant you still have sex with your partner, even after delivery you still have. And you will never know whether sometimes he has got another partner, so it is alright to use pills to protect yourself and the baby.

I: Can everyone take the daily oral PrEP while pregnant?

[Nonhlanhla]: No, not everyone there are people who don’t do well on tablets especially while pregnant, some vomit when they drink something, so it’s not everyone.

I: Let’s talk?

[India]: I am of the same opinion as [Nonhlanhla] has said that many people don’t like the tablets maybe they would prefer the ring.

[Juicylips]: I agree with [Nonhlanhla] people don’t like the pill when they are pregnant.

**FGD Number:** FGD_41-F24

**Site:** South Africa

**Excerpt Range:** 81766-83241

Would oral PrEP be something you would be interested in using while pregnant or breastfeeding, you here and not other people outside this discussion?

Respondents: Yes.

I: I noticed [Juicylips] seems to be surprised, so as you are pregnant would you take this pill to prevent HIV?

[Juicylips]: It will depend on how it [PrEP] treats me because I may say that I will take it while it will not treat me well. It will need nurses’ involvement so that they could intervene if it does not treat me well.

I: [Pink] What do you think?

[Pink]: With me pills is not my thing, so I cannot promise when I will take or not take it. I think the ring would be better for me.

I: This pill if you take it at 8.am. you must then take it at 8.p.m. all the time?

[Pink]: I might forget you see. It is better if you inserted the ring that’s it, but with the pill no.

I: Do you think you might forget to take these pills at the same time every day?

[Nonhlanhla]: I might forget.

[Makhosi]: I would not forget, I would have to set an alarm to remind me to take them, when the alarm goes off, I would take it.

I: I see [Grey] is like…?

[Grey]: I think you might forget because sometimes you would sleep out, and it would be hard to leave someone’s house you are visiting.

I: Someone’s house, you mean at a boyfriend’s house?

[Grey]: Yes, and then maybe you didn’t take them with you, or you forgot them at home and 8 a.m. has passed you have not taken them, so you will be defaulting.

**FGD Number:** FGD_41-F24

**Site:** South Africa

**Excerpt Range:** 87303-89025

I: How do you think this pill will interfere with women’s use of contraceptives?

[Pink]: I think if we can ask a doctor how it can interfere with what you will be using whether pills or injection, so it would be better to talk to a doctor first.

I: But do you think daily oral PrEP can interfere with your use of contraceptives?

[India]: I don’t think so because with oral PrEP, you will use it daily and contraceptives you will use them once a month and you will skip other months, so I don’t think there will be a problem.

I: Okay. If a woman is using this pill daily [PrEP] and use the contraceptive pill daily, do you think that can cause a disturbance or a problem?

[Nonhlanhla]: There could, because you will have to set a certain time for one pill and another time for the other pill, you see now you must set the alarm for one pill and another time for the other pill…

I: If someone is using the contraceptive pill…?

[Nonhlanhla]: Yes, the contraceptive pill.

I: What about a person who is using contraceptive injection like the 2 months or 3 months one. Will there be a disturbance or problem?

Respondents: No.

I: If someone is using the injection and the pill?

Respondents: Yes.

I: Why then should there be a disturbance or a problem when you take PrEP and the contraceptive pill?

[Nonhlanhla]: Because you will be taking the pills every day, and you will have a time for the PrEP and a different time for the oral contraceptives, so for the injection, you only have it after two months and you know on which date you must go for your injection, that’s it, you are only busy with the PrEP.

I: But what kind of disturbance would be experienced?

[Nonhlanhla]: There is a possibility of forgetting.

**FGD Number:** FGD_41-F21

**Site:** South Africa

**Excerpt Range:** 74427-75864

I: Would oral PrEP be something you would be interested in using while pregnant or breastfeeding?

Ngwanenyana: Hence I have said before that I don’t take any medication that is not prescribed by my doctor. So I don’t think I would be interested in using it unless it is prescribed by my doctor that I must use.

Red: I think if they can say okay PrEP is allowed to be taken by pregnant or breastfeeding women I think I would be okay.

I: Why?

Red: Just because PrEP prevents someone from being infected, yes.

I: Others, would you be interested?

Asanda: For me I don’t think so because it’s the first time I hear about it and I am not quite sure how it works unless I have observed it from someone that it treated her alright, so I don’t think so.

I: What worries do you have about using oral PrEP and why, besides the side- effects, besides that it might affect the baby to come out not right?

Pink: As you know people are not the same so it might work for instance for Ngwanenyana and for me it takes its time or it doesn’t work at all.

I: PK what do you think?

PK: What was the question?

I: Taking PrEP while pregnant or breastfeeding, do you have any worries about taking it while pregnant or breastfeeding?

PK: Yeah, it might not work for me…

I: How?

PK: You will find that maybe you forget to take it.

I: Mpho?

Mpho: I don’t think I will have a big problem with it especially if it helps me prevent HIV/AIDS, yeah.

**FGD Number:** FGD_41-F21

**Site:** South Africa

**Excerpt Range:** 77951-79543

I: How do you think taking a pill every day will interfere with your daily lives?

Lisa: I don’t think it will interfere in a bad way if I may say in my life because after all it’s not a stress taking a pill, you just take a pill and swallow it that’s it. It is not like have to dig and put someone in hole, you just get water and take a pill and be strong.

I: Depending on the culture it may be permitted or taboo to take bitter medicine while pregnant how will this interfere with the women in your community’s abilities to take oral PrEP while pregnant?

Lisa: I think packaging on this one will play a big role because you said it was used for HIV before, if its packaging it’s the same as the packaging for HIV pills and we get them from the clinic we queue with people…Like…It’s going to be a problem. [People will say] “I have seen her at clinic carrying a blue packet of the tablets, it’s those ones, it means she is like that,” you know stigma.

I: Okay I hear Lisa, what do others think, what taboos exists in the community about taking bitter medicine while you are pregnant, what do or what will people say?

Red: People always talk even if you can be pregnant for instance as for me when I am pregnant I have an ulcer problem, so they were saying of course it doesn’t go alone it has a companion, they will first treat it and then treat your other problem.

I: What to do they mean, it doesn’t go alone?

Red: They mean that when you are HIV positive you also get another disease, like others would have TB [tuberculosis], others would have diabetes and others would have ulcer.

**FGD Number:** FGD_41-F21

**Site:** South Africa

**Excerpt Range:** 86105-87417

I: Okay. What are other local taboos or practices will make using vaginal ring while pregnant difficult, are there any taboos you know of in your community that may make a person or you unable to use the vaginal ring while pregnant?

Ngwanenyana: I don’t think there are because I think the ring is the new product in our communities so most people don’t know about it, that’s why there are not.

I: Okay. Would pregnant women in the community want to use products like this, in your opinion about other women where we live?

Apple: Not really because there are many stereotypes, there was this female condom it still exist but then most of the women don’t know about it. Or how to use it because it is a stereotype of saying you have to wait for certain hours before you sleep with the guy, so which they say it is useless because the[male] condom you just insert and have sex. Why with the female you have to wait for certain hours to use it, so hence for this one it will also take time for people to intend to use it, because there is a female condom that people don’t use it at all. And now they have introduced Max male condoms with flavors and so on and they are like boom, everyone knows about them because they have got flavor and so on, while female [condoms] they don’t take them into consideration.

**UGANDA:**

**FGD Number:** FGD_41-F62

**Site:** Uganda

**Excerpt Range:** 50412-51684

I:But do you think that women might use that tablet? Pregnant and breastfeeding women, will they use that tablet?

Chorus:Yes, they can use it.

I:Would you think that there are certain things that might stop them from using the Oral PrEP?

[Barbra]:I think that tablet might increase a woman’s appetite yet she doesn’t have what to eat and she is pregnant. She might also swallow it and it causes some side effects like dizziness and others…but if it doesn’t have side effects then women might be able to take it.

R:Why I think we might be able to take it, men claim that they do not have time, when you ask him to go for testing they will always say that they do not have time.

I:What might stop women from using that tablet?

[Carol]:Some women fear taking daily tablets…and the other thing, her partner might ask “Why are you taking that tablet?” he might think that “This woman got infected and she never told me.” You know you can’t hide something forever to a partner you stay with at home, he might get to know about it and it brings trouble to you. You might have hidden it from him because you do not want him to engage into sex with other women after knowing that you are taking ARV tablet to keep you safe but if you hide it that might also cause problems at home.

**FGD Number:** FGD_41-F62

**Site:** Uganda

**Excerpt Range:** 51685-52022

I:What can be done to make it easier for women to use this tablet?

[Agatha]:To make it easy for anyone to take tablets, there is need to take a lot of drinks like juice, eat well…that might make it easy food you to take your tablets.

I:What else?

R:It might push some women who have been faithful to their partners to start promiscuity.

**FGD Number:** FGD_41-F61

**Site:** Uganda

**Excerpt Range:** 50901-52138

I: Now, would that pill be something you would have wanted to use during pregnancy?

Chorus: Yes.

R: No.

I: During pregnancy?

Samantha: I would think that a vaginal ring is better.

I: I want us to first talk about the pill, we will also talk about the vaginal ring.

Sarah: I think I would take it because it would stop me from worrying about getting infected with HIV.

Annet: I would think it is not a good option for me because I do not like pills.

I: Why don’t you like pills?

Annet: Just thinking about it I vomit.

Pamela: It is not something I would like to use because I can forget to take it.

Aida: I do not have any problem with taking pills but my question is what if I forget to take it on that day.

I: If you forget to take it?

Aida: If you forget to take it on that day, what do you do?

Samantha: On my side, I would say no to that pill, after seeing the other woman’s experience who got a miscarriage when she started taking those pills I can’t take them.

Vanessa: It might not work for me because during pregnancy I vomit a lot and that makes me doubt whether it can stay inside when I vomit.

Shanitah: I think I would take it to stay safe in case my partner engages in sex with other women.

Angel: I also think I can use it.

**FGD Number:** FGD_41-F61

**Site:** Uganda

**Excerpt Range:** 52139-53745

I: What are you worried about this tablet?

Sarah: We are told that if someone starts taking that medicine and then stop you can die. My question is “Does that happen with only those who are infected with HIV or even those who are not infected?”

I: What did we say the tablet is for?

Chorus: Prevention.

Samantha: I heard that when the blood gets used to medicine it weakens [immunity reduces] if one stops taking that medicine. So, that would mean that in case I start taking that medicine and then stop my immunity will also reduce where it can’t fight off the virus. That would mean that I have to take it forever just like HIV positive patients.

Pamela: What might stop me from taking that tablet, I might have not told my partner that I am taking it and we get misunderstandings when he finds out.

I: What might he think in case he found out?

Pamela: That maybe I have a certain infection that I am treating.

I: How about the rest, what worries do you have about this tablet?

Ritah: Now, in case you are pregnant and you have to take them daily, don’t they have some side effects? Won’t they affect the baby who is inside the womb?

Annet: We are told that HIV can take some time before you get to know that you have it. What if I start taking it when I have it, won’t that cause some problems?

I: That is what you are worried about?

Annet: Yes.

Aida: Doesn’t that tablet cause some side effects if taken daily?

I: Anyone else with some worries?

Sarah: We are told that when you take that tablet it causes dizziness, you lose energy in your body…does that also happen to me who is not infected with HIV?

**FGD Number:** FGD_41-F61

**Site:** Uganda

**Excerpt Range:** 63469-64833

I: Now, would you think pregnant and breastfeeding women from where you stay might want to use such things?

Ritah: It is acceptable the moment you know the purpose of it like if you get to know that it is used to prevent HIV then you will be allowed to use that medicine or the vaginal ring.

Pamela: People will be so happy about it when they get to know it because they are tired of the HIV infection. They will not be worried anymore.

Angel: Some women will decline to use it saying that, “the Whites have come to kill us just like it is for family planning methods.” When they give birth to babies who have some disabilities they say that it was due to family planning methods. So, I think some women will say that it is about Whites being trying to get involved in everything.

Samantha: To add on what she has said, some women will say that Whites have always tried to kill us; those are Whites’ tricks. So, some will accept and others will decline.

Annet: I think people will like it so much but won’t it increase promiscuity? Because they won’t be worried of anything.

I: It might increase promiscuity…

Annet: Yes.

I: Who else hasn’t told us anything?

Sarah: I think most people will accept [to use it] because we are many mothers and we are the ones who are in most need of staying healthy. I think women will accept to use it because they do not trust men.

**ZIMBABWE:**

**FGD Number:** FGD_41-F43

**Site:** Zimbabwe

**Excerpt Range:** 39084-39414

what are the things that are prohibited when someone is pregnant or breast feeding that can affect the uptake of PrEP?

Maka: Can you come again?

I: What other local taboos or practices would make taking oral PrEP while pregnant difficult?

Maka: Alcohol consumption is prohibited, so maybe that can affect the working of the pill.

**FGD Number:** FGD_41-F43

**Site:** Zimbabwe

**Excerpt Range:** 41600-42343

I: So if you were to be given the ring today to use to prevent HIV during pregnancy and breast feeding, would you be interested in using the ring?

Tsitsi: Personally I think the ring is better. These pills are tricky, if your family members start to see you taking these pills every day they will not understand you. Even when you try explaining that it is to prevent HIV some of our parents might take long to understand or might even think that you are on ART.

I: What do you think would make the ring difficult to use?

Linda: I think the ring is too hard, (baby cries).

Shami: I think other people might not insert it correctly then it affects things in there.

Maka: I think the man might equate the ring to a condom and refuse to use it.

**FGD Number:** FGD_41-F41

**Site:** Zimbabwe

**Excerpt Range:** 44058-45203

I: Uhm. Anyone who wants to add? It is fine. How would the tablet interfere with your daily life, say you are now taking PrEP, how would it interfere with your daily life, things like your social life, sex life, how would it interfere, Nyasha?

Nyasha: I don’t think it will interfere with anything.

I: Uhm, why?

Nyasha: Because it is just like other pills that we have always been taking on a daily basis.

Tendai: It is good, I am not being negative.

I: Yes.

Tendai: But sometimes like what the family planning pills do, you can take and have side effects like a headache. In that case can you come back to report your challenges and have them changed.

I: Jane?

Jane: Since we do not know how this pill works, it might be having some side effects like what Tendai is saying. For example, the family planning pill, I have 8 years taking them. Every morning I would wake up vomiting like someone who is pregnant. So when you take this pill for a long time, won’t it cause some things in your life?

I: Uhm.

Jane: Such things can now affect your daily life because in the morning you can fail to do your daily chores feeling dizzy or something.

**CODES:** PILL (T), FACILITATORS (T), ACCEPTABILITY (F)

**TRANSCRIPTS:** ALL P&BF WOMEN FGDs

**MALAWI:**

**FGD Number:** FGD_41-F84

**Site:** Malawi

**Excerpt Range:** 64320-65312

I: So our friends have shared with us what would encourage them to take this pill…yes Monica, do you have different views from what our friends have shared?

[Monica]: [sighing] …no everything is okay.

I: What would encourage you to take the drug every day?

[Monica]: For me I would opt for the ring.

I: You would opt for the ring…would you share with us why you have chosen the ring and not the pill…that is your right…but we just want to know what would stop you from taking the pill every day?

[Monica]: Pills are bitter.

I: Oh bitterness…so that would make you choose the ring?

[Monica]: Yeah… it thought the ring remains inserted for a month?

I: Yes, for a month…so what is the main reason you would choose the ring?

[Monica]: The ring has pleased me and not the pills.

I: How has it pleased you?...please clarify on that.

[Monica]: It has pleased me that the ring can remain for a month.

I: A month still inserted?

[Monica]: Unlike taking the pill daily…I can easily forget.

**FGD Number:** FGD_41-F84

**Site:** Malawi

**Excerpt Range:** 69882-70901

others have already said their views that they would prefer the ring and they have also explained their reasons… for us after seeing the ring, what are our thoughts about the ring as a way a woman can use by inserting to protect themselves from HIV?

[Debora]: I would prefer the ring for the following reasons; with the ring on its own, once inserted one can move about with it, you can have sex with the husband just as you always do without any problem so I think the ring is much better because it is done once in a whole month…so I would prefer the ring because I can’t forget, I can just be staying until the month ends.

I: Alright…so in your view we can say that between the two you have opted for the ring and not the other method.

[Memory]: I also would prefer the ring…why for the pill, you have said that once one forgets to take it and you have sex, it means you are at risk, while the ring, once inserted it is until you remove it…[giggling]…no one will see it and you can’t forget…so I think it is good.

**FGD Number:** FGD_41-F81

**Site:** Malawi

**Excerpt Range:** 27110-28344

I: So let us first discuss about PrEP, the daily oral tablets. What are your first thoughts when you think of taking the oral PrEP tablets to protect yourselves from acquiring HIV while you are pregnant?.. What are our thoughts when we have heard about this issue? [Baby chuckles]

Favour: I feel this method of taking oral tablets is very helpful because when you are pregnant, you will not be worried of getting infected at any time. You will know that you are protected, together with the child you are expecting.

I: Thank you. Is there anyone with a different thought from that of Favour?

Lucy: I just want to add on what Favour has said, that these tablets can really help us because men do whatever they want when we are pregnant. They take it that we are tired and think of all sorts of things for us and they also take advantage of the same time to go out with other women. So, if you use these tablets to protect yourself from HIV, then you and your unborn child will be protected from acquiring the virus. .

Patricia: You cannot get worried when you become pregnant, especially, when you start Antenatal care, since you know that you are okay because you are taking tablets to protect yourself from HIV infection.

**FGD Number:** FGD_41-F81

**Site:** Malawi

**Excerpt Range:** 28346-29122

I: Are there any different thoughts from the ones that have been said?... What about the time we are breastfeeding, what are our thoughts in using these tablets when we are breastfeeding?

Kheliwe: The time we are breastfeeding, it may happen that the baby is just fine and he was not infected, and even after having a test it shows he is not infected, but if the husband goes his own ways, and gets infected, he will then pass on the infection to the mother. Since the baby is breast feeding, he may get infected through the mother’s breast milk. [Sound of driller in the background]So, this is worrisome to the mother. So, it would be very helpful to use the oral tablets method while breast feeding, in order to protect yourself as well as the baby from getting infected.

**FGD Number:** FGD_41-F81

**Site:** Malawi

**Excerpt Range:** 38606-38943

Favour: And also this vaginal ring I feel is a good method because when you put it once, it means it is there for the whole month and you will be using that very same ring while with tablets you may forget due to being occupied, while with the ring, once you insert it, you3 are done and you just have to remember the date to change it.

**FGD Number:** FGD_41-F81

**Site:** Malawi

**Excerpt Range:** 43803-45211

So, beside the doctor, who can get involved in making decisions on whether a woman can use the products we have discussed, ring and oral tablets when she is pregnant or breastfeeding. Besides the doctor explaining to you the importance of ring and tablets that should protect, who else do you think can encourage us in the communities?

Esther: Friends we chat with can encourage us.

I: When you say friends, do you mean males, those we sleep with or...?

Esther: No, just mere friends.

I: Your fellow women, the ones you chat with, eh? Is there anybody else we think can encourage us, or with whom we can encourage each other to be using these products in order to protect ourselves from acquiring HIV?

Favour: The chiefs are also supposed to take part in our communities in encouraging people to take these products.

I: Thank you very much. Are there some more people whom we think can encourage us, we have talked about chiefs, friends.

Kheliwe: Health Surveillance Assistants.

I: Health Surveillance Assistants who are working in our communities eh?

Kheliwe: Yes.

I: What about our mothers and mother- in- laws?

Lucy: It will depend on what kind of mothers they are. There are some mothers that like traditional herbs so much, so such type may not encourage you to take these products. But there are others who believe that traditional herbs are not good, they the type that can encourage you.

**FGD Number:** FGD_41-F81

**Site:** Malawi

**Excerpt Range:** 45212-45892

I: Thank you. What are the things that we think are important that can help in encouraging women to use the products we have discussed with you when they are pregnant or breastfeeding?

Esther: Can you repeat the question, I did not understand it?

I: What are the important things in our communities that can help in encouraging women to use the products we have learnt today?

Esther: Organizations can help in discussing with people and encouraging them in their communities.

I: Is there any different thought from that one?

Ethel: We are encouraged if the message goes to the chief, and then the chief organizes a meeting with the youth and shares with them these messages.

**SOUTH AFRICA:**

**FGD Number:** FGD_41-F24

**Site:** South Africa

**Excerpt Range:** 80714-81755

What about while breastfeeding what do you think about taking daily oral PrEP?

[Nonhlanhla]: I think the pills are alright you just take it and swallow it, it doesn’t go to the breast, not this saying that everything you consume goes to the breast I don’t think it is going to go there, maybe it will just protect, it is airtight.

I: I heard [Makhosi] is responding…?

[Makhosi]: The pill is alright because…

I: We are talking about while breastfeeding and taking this pill...?

[Makhosi]: Yes, because it will protect the baby also.

I: I heard you commenting about whether its goes to the breast while [Nonhlanhla] was still talking…?

[Makhosi]: Yes, it does go because it melts and goes there. It goes through the veins right, yes, it goes there, and it is alright.

[Grey]: I think it should be the clinic or hospital that tell you whether you should take it or not while breastfeeding. There might be chances that it affects or not, so I think it should be the professionals who tell you whether it is okay for you take it or not.

**FGD Number:** FGD_41-F24

**Site:** South Africa

**Excerpt Range:** 81766-83241

Would oral PrEP be something you would be interested in using while pregnant or breastfeeding, you here and not other people outside this discussion?

Respondents: Yes.

I: I noticed [Juicylips] seems to be surprised, so as you are pregnant would you take this pill to prevent HIV?

[Juicylips]: It will depend on how it [PrEP] treats me because I may say that I will take it while it will not treat me well. It will need nurses’ involvement so that they could intervene if it does not treat me well.

I: [Pink] What do you think?

[Pink]: With me pills is not my thing, so I cannot promise when I will take or not take it. I think the ring would be better for me.

I: This pill if you take it at 8.am. you must then take it at 8.p.m. all the time?

[Pink]: I might forget you see. It is better if you inserted the ring that’s it, but with the pill no.

I: Do you think you might forget to take these pills at the same time every day?

[Nonhlanhla]: I might forget.

[Makhosi]: I would not forget, I would have to set an alarm to remind me to take them, when the alarm goes off, I would take it.

I: I see [Grey] is like…?

[Grey]: I think you might forget because sometimes you would sleep out, and it would be hard to leave someone’s house you are visiting.

I: Someone’s house, you mean at a boyfriend’s house?

[Grey]: Yes, and then maybe you didn’t take them with you, or you forgot them at home and 8 a.m. has passed you have not taken them, so you will be defaulting.

**FGD Number:** FGD_41-F24

**Site:** South Africa

**Excerpt Range:** 108557-109916

What do you think is the most important factor that will motivate pregnant or the breast-feeding woman to use the ring or the tablets for HIV prevention?

[Makhosi]: I think the pill because they might be afraid to insert the ring while pregnant, but they can take the pill.

I: Others, we are done, and this is my last question, do you have your last closing comments?

[Grey]: Because human safety comes first, so to know that the ring and the pill can prevent HIV will motivate people to use the ring and the pill.

I: Anyone with a different view from [Makhosi] and [Grey]?

Respondents: No, we agree with them.

I: Alright, what should we do to encourage women to use these products?

[India]: I think you should encourage them by educating them about these products, as much as we have heard about them when we leave here we should also tell people about them.

[Makhosi]: There must be pamphlets that people can read.

[Grey]: I think it should be put up on social media because many people use the internet and social media almost every day, so people will go into the internet, learn about these products and gain knowledge.

I: What social media are you talking about?

[Grey]: YouTube and these posts we always put up on WhatsApp, and there must be a Facebook page about these products.

[Pink]: And they must educate about them in the clinics.

**FGD Number:** FGD_41-F21

**Site:** South Africa

**Excerpt Range:** 74427-75864

I: Would oral PrEP be something you would be interested in using while pregnant or breastfeeding?

Ngwanenyana: Hence I have said before that I don’t take any medication that is not prescribed by my doctor. So I don’t think I would be interested in using it unless it is prescribed by my doctor that I must use.

Red: I think if they can say okay PrEP is allowed to be taken by pregnant or breastfeeding women I think I would be okay.

I: Why?

Red: Just because PrEP prevents someone from being infected, yes.

I: Others, would you be interested?

Asanda: For me I don’t think so because it’s the first time I hear about it and I am not quite sure how it works unless I have observed it from someone that it treated her alright, so I don’t think so.

I: What worries do you have about using oral PrEP and why, besides the side- effects, besides that it might affect the baby to come out not right?

Pink: As you know people are not the same so it might work for instance for Ngwanenyana and for me it takes its time or it doesn’t work at all.

I: PK what do you think?

PK: What was the question?

I: Taking PrEP while pregnant or breastfeeding, do you have any worries about taking it while pregnant or breastfeeding?

PK: Yeah, it might not work for me…

I: How?

PK: You will find that maybe you forget to take it.

I: Mpho?

Mpho: I don’t think I will have a big problem with it especially if it helps me prevent HIV/AIDS, yeah.

**FGD Number:** FGD_41-F21

**Site:** South Africa

**Excerpt Range:** 92065-92766

I: Okay. What do you think is the most important factor that will motivate a pregnant or breastfeeding woman to use the ring or the tablet for HIV prevention?

Apple: While pregnant is the offspring because you are thinking about the offspring it is no longer about you as an individual you have to think about the offspring so yeah.

I: What about when breastfeeding?

Mpho: I think it’s the child because we all want to see how the child lives, right.

I: Mmh, what could be done to help facilitate or encourage women to use these products, the ring and oral PrEP?

Lisa: I think to educate women and to initiate more awareness campaigns, like these ones a woman will be more motivated to use these.

**UGANDA:**

**FGD Number:** FGD_41-F62

**Site:** Uganda

**Excerpt Range:** 51685-52022

I:What can be done to make it easier for women to use this tablet?

[Agatha]:To make it easy for anyone to take tablets, there is need to take a lot of drinks like juice, eat well…that might make it easy food you to take your tablets.

I:What else?

R:It might push some women who have been faithful to their partners to start promiscuity.

**FGD Number:** FGD_41-F61

**Site:** Uganda

**Excerpt Range:** 53746-54653

I: Now, what might help women who are pregnant and breastfeeding to take that tablet?

R: What might help women to take that tablet is the fact that a woman will not have any fear that she will get infected with HIV.

I: Not having fear about getting infected with HIV helps her to take the tablet?

R: She will take it because she wants to prevent HIV and she will not have that fear that she will get infected.

I: How about the rest?

Sarah: I was told that it is easy to know that you are infected during pregnancy. The fact that you have been tested several times during that period and you do not have the virus you will be assured that you will never get infected. You will be able to take it and complete the required period [period during pregnancy and breastfeeding] because you know you do not have HIV.

R: The fact that she knows she is fighting for her own health and the health of the unborn child.

**FGD Number:** FGD_41-F61

**Site:** Uganda

**Excerpt Range:** 63469-64833

I: Now, would you think pregnant and breastfeeding women from where you stay might want to use such things?

Ritah: It is acceptable the moment you know the purpose of it like if you get to know that it is used to prevent HIV then you will be allowed to use that medicine or the vaginal ring.

Pamela: People will be so happy about it when they get to know it because they are tired of the HIV infection. They will not be worried anymore.

Angel: Some women will decline to use it saying that, “the Whites have come to kill us just like it is for family planning methods.” When they give birth to babies who have some disabilities they say that it was due to family planning methods. So, I think some women will say that it is about Whites being trying to get involved in everything.

Samantha: To add on what she has said, some women will say that Whites have always tried to kill us; those are Whites’ tricks. So, some will accept and others will decline.

Annet: I think people will like it so much but won’t it increase promiscuity? Because they won’t be worried of anything.

I: It might increase promiscuity…

Annet: Yes.

I: Who else hasn’t told us anything?

Sarah: I think most people will accept [to use it] because we are many mothers and we are the ones who are in most need of staying healthy. I think women will accept to use it because they do not trust men.

**FGD Number:** FGD_41-F61

**Site:** Uganda

**Excerpt Range:** 64834-66006

I: Now, we have looked at the two products; the tablets and the vaginal ring. Which would be most preferred?

Angel: A vaginal ring.

I: You should also tell us the reason why women will like that product the most.

Angel: A vaginal ring because you insert it once for a month.

Ritah: Tablets because even men will be able to use them. They will be for both women and men.

I: Both women and men will be taking them…

Ritah: Yes, yet a man can’t insert a vaginal ring.

Annet: I think it is a vaginal ring because you insert it once and you do not have to keep inserting it every day.

Vanessa: I think it should be a vaginal ring because it doesn’t keep me worried of anything.

I: Which kind of worries are you talking about? How do

Vanessa: Having to remember to take medicines every day.

Sarah: I think it should be a vaginal ring because it is confidential.

Shanitah: Pills because I will be able to talk to my partner so that I do it he is in the know and we keep it to ourselves.

Samantha: I think it should be a vaginal ring because you won’t have to be worried whether you inserted it or not. And for any rumours that so and so found you taking ARVs will be eliminated.

**FGD Number:** FGD_41-F61

**Site:** Uganda

**Excerpt Range:** 70123-73057

I: Now, we would like you to advise us on how we could recruit pregnant and breastfeeding women in future research to see how these products work?

Ritah: Teaching them about the benefits of the tablets and the vaginal ring.

Annet: Identifying one of them who is either pregnant or breastfeeding and is using one of the products to speak to them. Someone who is using it to educate them when they come for their antenatal.

I: What do the rest think?

R: Those who have used it should convince them.

I: Now, looking at the video we have just played for you, do you think it would convince a woman to participate in this research or there is something that should be added?

Aida: I think we need to add what we have just said that someone who has used it might motivate women.

I: Someone who has used it to speak to people…

Aida: To speak to people and explain what she is experiencing.

Samantha: I also think that the video is lacking but if we include someone who has used it showing exactly when she is inserting it but not these cartoons you have just showed us I would think someone cannot decline to use them.

I: A video showing someone who has used it.

Samantha: Exactly and how she is inserting it.

I: And how she is removing it?

Samantha: Removing it would not be a problem but how she is inserting it is most important.

Aida: It is just like when you come to a hospital for delivery and you have fears but when you see someone delivering it can encourage you.

R: Those who have used it should testify that they have used it and it can prevent HIV.

I: Anything else? Angel?

Angel: What I was suggesting is that they should show how a person who has used the ring or tablet looks like, like we see how the HIV infected looks like and how the one who is not infected looks like.

I: How should the video show the person who has used the vaginal ring?

Angel: They should let us see who has used it before because there are people who have used family planning methods and they either gained weight or lost weight, so we need to see someone who has used the vaginal ring and someone who has swallowed the tablets before.

Sarah: We need to see a couple whereby a man is infected with HIV and they have used it and the woman hasn’t been infected, and then people will use it.

I: They should bring a couple…

Sarah: Yes…where one is infected and the other is not.

I: Where one who is HIV negative takes tablets?

Sarah: Yes…whereby they engage in sex and the one who is HIV negative remains negative. That is when it will become easy for them to understand that those products are effective.

Pamela: They should come to our communities and tell the people.

Samantha: I have a witness who was raped by an HIV positive man and she was given that medicine and within a few weeks she was tested and found HIV negative. The man eventually died.

I: Is there anyone who wants to add on something before we end?

[Silent].

I: Is that all?

R: Yes.

**ZIMBABWE:**

**FGD Number:** FGD_41-F43

**Site:** Zimbabwe

**Excerpt Range:** 33248-34214

Let’s start by talking about daily oral PrEP tablets. What is your first thought when you think about taking oral PrEP to prevent HIV while pregnant?

Tanya: As a pregnant mother, what first comes into my mind is the issue of side effects to me and the baby, because just like any other pill there are side effects. The other thing is, since my hormones are already tempered around with because of pregnancy, will the PrEP pill go down well with me?

Ropa: I will be very happy to take this pill to protect me from HIV since I have always taken the vitamin pills every day. In this case I will be protected from HIV so I will be happy.

Tsitsi: I will be happy to be protected since I don’t want to get HIV.

Tau: It’s okay because I and the baby will be safe.

Charlene: I think using these pills is better because we do not have anything to prevent HIV with. Maybe your husband might fail to understand you but if they do not have side effects that will be good for us.

**FGD Number:** FGD_41-F43

**Site:** Zimbabwe

**Excerpt Range:** 38522-38891

I: Depending on the culture, it may be permitted or taboo to take bitter medicine while pregnant. How would this interfere with women in your community’s ability to take oral PrEP while pregnant?

Charlene: I think that the fact that this pill now exist it means the doctors approved that we can use it. So if they say we can use it, there is nothing bad I see on that.

**FGD Number:** FGD_41-F41

**Site:** Zimbabwe

**Excerpt Range:** 38224-39208

Tendai: I think that if I take the PrEP pill it will help me protect my health.

I: Uhm.

Tendai: And my child too. I will get to deliver well without HIV.

I: Ok.

Tendai: That is my thought.

I: Those are your thoughts, Nyasha. If you think of taking PrEP pills to prevent yourself from getting HIV during pregnancy, what first comes into your mind? There are no right or wrong answers.

Nyasha: I agree with what Tendai has said. I will be considering the health of the baby I am carrying in my womb, that I will give birth to a child with good health and my health too will be safe.

I: Ok, Jane?

Jane: I just think that I will have peace of mind because I will be knowing that I am on the safe side. We might have been tested [During antenatal care] and told that we are fine [HIV negative], but you will always be thinking that, “Since the last time I tested, am I still alright [HIV negative], am I going to give birth to a child without HIV?” I think my mind will be at peace.

**FGD Number:** FGD_41-F41

**Site:** Zimbabwe

**Excerpt Range:** 39209-39606

I: Ok. What about when breastfeeding, when you think of taking this pill to prevent HIV, what comes into your mind first, TK?

TK: I will have the same thoughts because you will have prevented your child during breastfeeding because that is when most children contract diseases. So when you think of taking it you would have thought about your baby’s protection that she will be on the safe side.

**FGD Number:** FGD_41-F41

**Site:** Zimbabwe

**Excerpt Range:** 42806-43123

I: Alright. What would facilitate taking oral PrEP for P and BF women, Sarah?

Sarah: We would want our lives to be protected.

I: Alright, Jane?

Jane: The thought that this is where the health of my baby depends on will motivate me to take the pill; and also the thought that this is also where my health depends on.

**FGD Number:** FGD_41-F41

**Site:** Zimbabwe

**Excerpt Range:** 43149-43722

Tanatswa: I just wanted to support what Jane said that us women, if we really understand how important this pill is to our babies and to us as well, we will be able to accept it and take it every day.

I: Alright. How can women come to this understanding, what can be done for women to understand this, Tanatswa?

Tanatswa: I think if there can be community educators, they can be health workers or--, who can teach since these are new things that are coming in right.

I: Uhm.

Tanatswa: Teaching so that woman do understand. I think most women will be interested in it.

**FGD Number:** FGD_41-F41

**Site:** Zimbabwe

**Excerpt Range:** 45219-46258

Depending on the culture, it may be permitted or taboo to take bitter medicine while pregnant. How would this interfere with women in your community’s ability to take oral PrEP while pregnant?

TK: Can I have the question again?

I: Depending on culture right, it may be permitted or taboo to take bitter medicine when you are what,

All: When you are pregnant.

I: When you are pregnant right. Because of that culture, how can it interfere with the community’s ability to take oral PrEP while pregnant when people know that they are not supposed to take bitter medicines while pregnant?

Tendai: As something that was tested and seen that we can use it, I do not think it will be difficult when we are taught and we understand. I do not foresee any problems for us to use it because it was already tested by doctors and they saw it fit for us to use it. So we can accept it.

I: It is ok. Memory?

Memory: I do not think there is anything bad for us to use it because it was tested worldwide and was seen that it works.

I: Ok.

Memory: Uhm.

**FGD Number:** FGD_41-F41

**Site:** Zimbabwe

**Excerpt Range:** 46515-46933

Ok, what other local taboos or practices would make taking oral PrEP while pregnant and breastfeeding difficult, Tanatswa?

Tanatswa: From my own perspective, there is nothing that can affect the taking of PrEP. If it is something that was tested and other countries supporting that it is a good thing,

I: Uhm.

Tanatswa: It can be accepted.

I: Uhm.

Tanatswa: There is nothing that can hinder us from taking this pill.

**CODES:** RING (T), BARRIERS (T), ACCEPTABILITY (F)

**TRANSCRIPTS:** ALL P&BF WOMEN FGDs

**MALAWI:**

**FGD Number:** FGD_41-F84

**Site:** Malawi

**Excerpt Range:** 63308-63810

I: So, the way we have seen the tablet, what is it about the pill that would make the women not to take it daily?

[Triza]: The problem with the pill is being forgetful… if we forget to take the contraceptive pills…we do remember late, waking up past something…to take the pill…[laughing]…so the pill, I think for those that can remember they can take it but not for those that are forgetful like me…I can’t try it…[giggling]…it is better I opt for the ring because I will just stay with it…[laughing]

**FGD Number:** FGD_41-F84

**Site:** Malawi

**Excerpt Range:** 64320-65312

I: So our friends have shared with us what would encourage them to take this pill…yes Monica, do you have different views from what our friends have shared?

[Monica]: [sighing] …no everything is okay.

I: What would encourage you to take the drug every day?

[Monica]: For me I would opt for the ring.

I: You would opt for the ring…would you share with us why you have chosen the ring and not the pill…that is your right…but we just want to know what would stop you from taking the pill every day?

[Monica]: Pills are bitter.

I: Oh bitterness…so that would make you choose the ring?

[Monica]: Yeah… it thought the ring remains inserted for a month?

I: Yes, for a month…so what is the main reason you would choose the ring?

[Monica]: The ring has pleased me and not the pills.

I: How has it pleased you?...please clarify on that.

[Monica]: It has pleased me that the ring can remain for a month.

I: A month still inserted?

[Monica]: Unlike taking the pill daily…I can easily forget.

**FGD Number:** FGD_41-F84

**Site:** Malawi

**Excerpt Range:** 70903-72495

I: What concerns could a woman have about the ring even though you have said that it is fine than the pill…what concerns can a woman had concerning the ring?...the concerns could be on the breast milk, her health… what could be the concerns?

[Angel]: My concerns could be that, when the man is making love to you, can’t he be pushing the ring further inside?...mmmh…or push it sideways…can’t that happen?

[silence]

I: Is there anyone who can respond to [Angel]?

[Memory]: I have heard that once it is inserted the man cannot notice…like reaching it or anything.

I: From what you have heard from the video, right?

[Memory]: There is any cause for worry about the man feeling the ring or pushing it deep inside…no, once inserted it is done.

[Debora]: Let me add on that…I also asked the same question when we were at that other place…so they responded that, there was a certain family where the woman told the man that, ‘ I inserted such a thing, so the man said that, I can’t feel any change in the way we have sex, there is no change and I can’t feel it’…..but the woman said, but I inserted it….the man said not you are lying to me, let’s go to the hospital for me to verify… so when they came here and explained to the people that were here, they took them to a room and the woman laid down and the doctor inserted his fingers and brought out the ring and the man said, aaah that is very good, I never knew anything’…yeah so I would answer her like that maybe she can feel good.

I: [Angel], has your question been answered?

[Angel]: Yes.

I: Has it been answered?

[Angel]: Yes.

**FGD Number:** FGD_41-F81

**Site:** Malawi

**Excerpt Range:** 35822-37084

Now let us discuss on the vaginal ring. What are your first thoughts on using the vaginal ring to protect yourself from HIV while you are pregnant? what are our thoughts in inserting the ring in the vagina?...some of us have heard it for the first time that there is a ring that is inserted down there and some of us heard it before, when we heard about this, what were our thoughts?

Lucy: When I heard about the ring, I felt on the other hand it is good and on the other hand it is not good. The good part of it is that I felt the ring is going to help in women’s' life because when we have inserted the ring, we have all the possibility of being protected from HIV/AIDS which you may get without knowing it or even by ourselves, as women if we sleep around. So using this ring in such circumstances, it can help them in different ways. Now on the other hand, where I feel the ring can give us problems, is because some people say it is not good to be inserting fingers/things on the cervix as this may cause some things or even cancer or introducing bacteria and the time you want to insert the ring it will be that you will be inserting every now and then which can also give some little problems when we are frequently inserting and re inserting the ring.

**FGD Number:** FGD_41-F81

**Site:** Malawi

**Excerpt Range:** 37219-38515

What about when we heard about using the ring when we are breastfeeding? What are our worries in using the ring and why do we have them?.......It can be worries concerning our life, or worries concerning our baby, or worries concerning our husband or any other worries we might have, what are they?.....

Esther: When you are using the ring and you are pregnant or breastfeeding you cannot be worried when you have the ring because you know that you are protected from infection. But sometimes, worries can be inevitable because problems are a part of a person’s life, they don’t come to an end. There are so many worries people can have different of them.

I: So I want you to share with us those worries? [Laughs] The lots of worries that you say are there, what are they?

NT: Concerning the use of the vaginal ring?

Kheliwe: There can be a worry that the husband has not agreed, he is not happy that you should put something down there. So, you may be worried that if you insert it, will he be happy or not.

I: Is there anymore addition? Thank you Kheliwe. Is there anymore addition there? ...What things do you think can encourage women to use this vaginal ring?...

Lucy: I feel women can be encouraged to use the ring so that they should protect themselves from HIV/AIDS disease.

**FGD Number:** FGD_41-F81

**Site:** Malawi

**Excerpt Range:** 38968-39710

How do you think the ring can disturb our daily life?...

Kheliwe: It cannot disturb our daily life because as we have seen here, it does not have any problems. When you have inserted the ring, you do not feel anything and you can freely move around and don't think about anything.

I: Thank you very much. Is there any addition?

Lucy: I feel the ring can disturb your daily life, because it is a new thing in your life. When you have inserted that thing, and you are not yet used, especially if it’s at the beginning, from time to time, you may feel like it has come out or keep asking yourself if it’s in place or if it has moved every time you have had sex. You can somehow be in a disturbed state because you are not yet used to it.

**FGD Number:** FGD_41-F81

**Site:** Malawi

**Excerpt Range:** 41212-41753

I: Are there some cultural beliefs or taboos that we feel can interfere in using the ring?

Esther: Yes, there are, like some religions. Believers from these religions do not believe in using any type of medications on their bodies.

Ethel: There are still some who believe in traditional practices and discourage others from going to the hospital or following hospital guidance. There are times when one understands something and explains it to the elders/parents, but they still stop her from using the product or following the guidance.

**FGD Number:** FGD_41-F81

**Site:** Malawi

**Excerpt Range:** 41754-42465

I: What else can stop women from using the vaginal ring besides what has been said?... Let us start with you Lucy then Kheliwe.

Lucy: There are some men who do not understand things easily, so, when you raise this issue of ring they may not accept it. As it is up to us women to take a family planning method, so we do likewise here, by explaining to the husband that there is this family planning method, that we should be using a ring. He may take it to be a new thing to him and may not accept it. The challenge comes in because most women in the families would like to protect and save their marriages, so in an attempt to do that, they listen to their husbands and may think of not using the ring at all.

**FGD Number:** FGD_41-F81

**Site:** Malawi

**Excerpt Range:** 43803-45211

So, beside the doctor, who can get involved in making decisions on whether a woman can use the products we have discussed, ring and oral tablets when she is pregnant or breastfeeding. Besides the doctor explaining to you the importance of ring and tablets that should protect, who else do you think can encourage us in the communities?

Esther: Friends we chat with can encourage us.

I: When you say friends, do you mean males, those we sleep with or...?

Esther: No, just mere friends.

I: Your fellow women, the ones you chat with, eh? Is there anybody else we think can encourage us, or with whom we can encourage each other to be using these products in order to protect ourselves from acquiring HIV?

Favour: The chiefs are also supposed to take part in our communities in encouraging people to take these products.

I: Thank you very much. Are there some more people whom we think can encourage us, we have talked about chiefs, friends.

Kheliwe: Health Surveillance Assistants.

I: Health Surveillance Assistants who are working in our communities eh?

Kheliwe: Yes.

I: What about our mothers and mother- in- laws?

Lucy: It will depend on what kind of mothers they are. There are some mothers that like traditional herbs so much, so such type may not encourage you to take these products. But there are others who believe that traditional herbs are not good, they the type that can encourage you.

**SOUTH AFRICA:**

**FGD Number:** FGD_41-F24

**Site:** South Africa

**Excerpt Range:** 77823-80703

I: Okay as explained before we are interested in getting your opinions about the two different products that women can use for HIV prevention, the daily oral PrEP tablets and the monthly vaginal ring. Let’s start by talking about daily oral PrEP tablet, what is your first thought when you think about taking oral PrEP to prevent HIV while pregnant?

Nonhlanhla: I think it’s alright to prevent while you are pregnant and after…The pill is alright.

I: After when?

[Nonhlanhla]: Like after you have found out that you are pregnant, and you have tested and know about your status then its fine, the pill is alright to take.

I: Why do you say the pill is alright?

[Nonhlanhla]: I fear the other one… [The vaginal ring].

I: What are you scared of?

[Nonhlanhla]: What is it…?

I: The ring…?

[Nonhlanhla]: Yes, it scares me, so I prefer the tablets.

I: Others what do you think of taking the pills to prevent HIV while pregnant?

[Makhosi]: I also prefer the pill I am also scared of it [the ring] what if…Isn’t that the sizes of the vagina are not the same, what if you can’t get it in or while inside it doesn’t come out.

I: Why do you say you prefer tablets, I want us to talk about the pills now and we will then discuss the ring afterwards? I want to talk about whatever things that may make you prefer to use the tablets or not prefer the vaginal ring, let’s talk about those?

[Grey]: Okay earlier when we were sitting on that side and the lady who was reading the informed consent for us she said these pills can help for both oral and vaginal sex because there are those who prefer oral sex to vaginal sex. Then you can help for both oral and vaginal sex.

I: How can they help with that?

[Grey]: They prevent infections through both vaginal and oral sex that is what she said to us. I think it is best to take the pills for when your partner wants to do oral sex they will help you because the ring protects only the vagina.

I: Okay we have heard [Grey], what do others say about taking daily oral PrEP to prevent HIV? Let’s talk so that we can finish.

[Pink]: We agree that it is better to take the pills to prevent…

I: While pregnant…

[Pink]: While and after because when you are pregnant you still have sex with your partner, even after delivery you still have. And you will never know whether sometimes he has got another partner, so it is alright to use pills to protect yourself and the baby.

I: Can everyone take the daily oral PrEP while pregnant?

[Nonhlanhla]: No, not everyone there are people who don’t do well on tablets especially while pregnant, some vomit when they drink something, so it’s not everyone.

I: Let’s talk?

[India]: I am of the same opinion as [Nonhlanhla] has said that many people don’t like the tablets maybe they would prefer the ring.

[Juicylips]: I agree with [Nonhlanhla] people don’t like the pill when they are pregnant.

**FGD Number:** FGD_41-F24

**Site:** South Africa

**Excerpt Range:** 81766-83241

Would oral PrEP be something you would be interested in using while pregnant or breastfeeding, you here and not other people outside this discussion?

Respondents: Yes.

I: I noticed [Juicylips] seems to be surprised, so as you are pregnant would you take this pill to prevent HIV?

[Juicylips]: It will depend on how it [PrEP] treats me because I may say that I will take it while it will not treat me well. It will need nurses’ involvement so that they could intervene if it does not treat me well.

I: [Pink] What do you think?

[Pink]: With me pills is not my thing, so I cannot promise when I will take or not take it. I think the ring would be better for me.

I: This pill if you take it at 8.am. you must then take it at 8.p.m. all the time?

[Pink]: I might forget you see. It is better if you inserted the ring that’s it, but with the pill no.

I: Do you think you might forget to take these pills at the same time every day?

[Nonhlanhla]: I might forget.

[Makhosi]: I would not forget, I would have to set an alarm to remind me to take them, when the alarm goes off, I would take it.

I: I see [Grey] is like…?

[Grey]: I think you might forget because sometimes you would sleep out, and it would be hard to leave someone’s house you are visiting.

I: Someone’s house, you mean at a boyfriend’s house?

[Grey]: Yes, and then maybe you didn’t take them with you, or you forgot them at home and 8 a.m. has passed you have not taken them, so you will be defaulting.

**FGD Number:** FGD_41-F24

**Site:** South Africa

**Excerpt Range:** 93927-94964

I: What worries do you have about the ring and why?

[Nonhlanhla]: My first worry is that you will insert it and then it doesn’t want to come out, and you will be searching for it and don’t know where to search then you must go to the doctors to search for it…They will be navigating your vagina more.

[Makhosi]: And you might be hurting yourself.

[Nonhlanhla]: You might be hurting yourself, then you must go to the doctors and they will be navigating your vagina more, that’s my main worry.

[Grey]: I agree with her. Do you remember the time they had just introduced the Implant? Others were saying it disappeared from their arms they could not locate it. So, what if it dislodges and you can’t find it, I agree with her.

I: Okay, any other worries about using the vaginal ring?

[Makhosi]: Maybe while having sex he will be pushing it further and further, it goes up and up…

[Nonhlanhla]: Until it gets lost.

[Pink]: I want to know if you have inserted it how will you know that its sitting properly where it’s supposed to sit.

**FGD Number:** FGD_41-F21

**Site:** South Africa

**Excerpt Range:** 82450-83875

I: Do you think using the vaginal ring will interfere with your daily life?

Lisa: If the vaginal ring is inside and people are able to forget about it because…As Apple said it is like a tampon, so I don’t think it can interfere. Even the tampon is worse because it gets full and you can feel it is full but the vaginal ring stays there for a month so I don t think it can interfere with your life. But as Mpho has said if it can fall out, yeah.

I: So when you look at pregnancy what do you think is the right time for someone to use the ring, first, second or last tri-mester?

Ngwanenyana: I think since pregnant women are not allowed to insert anything in your vagina while pregnant so I don’t think you can use it while still pregnant.

I: Mmh, oh pregnant woman is not supposed to insert things in the vagina while pregnant?

Ngwanenyana: Yes.

I: What do they say happens? I am almost done I am going to the last page of my questions so if you can talk now and we will finish soon?

Ngwanenyana: It is not like they explain what happens when you insert things in your vagina while pregnant but doctors and gynecologist always advise us not to insert anything in the vagina.

I: Okay. Red?

Red: Some doctors do say if you insert some things here, those things do affect the head of the baby and the baby might become slow and the baby’s eyes might have a discharge, and it can lead do wounds and a baby will end up blind.

**FGD Number:** FGD_41-F21

**Site:** South Africa

**Excerpt Range:** 86105-87417

I: Okay. What are other local taboos or practices will make using vaginal ring while pregnant difficult, are there any taboos you know of in your community that may make a person or you unable to use the vaginal ring while pregnant?

Ngwanenyana: I don’t think there are because I think the ring is the new product in our communities so most people don’t know about it, that’s why there are not.

I: Okay. Would pregnant women in the community want to use products like this, in your opinion about other women where we live?

Apple: Not really because there are many stereotypes, there was this female condom it still exist but then most of the women don’t know about it. Or how to use it because it is a stereotype of saying you have to wait for certain hours before you sleep with the guy, so which they say it is useless because the[male] condom you just insert and have sex. Why with the female you have to wait for certain hours to use it, so hence for this one it will also take time for people to intend to use it, because there is a female condom that people don’t use it at all. And now they have introduced Max male condoms with flavors and so on and they are like boom, everyone knows about them because they have got flavor and so on, while female [condoms] they don’t take them into consideration.

**UGANDA:**

**FGD Number:** FGD_41-F61

**Site:** Uganda

**Excerpt Range:** 50901-52138

I: Now, would that pill be something you would have wanted to use during pregnancy?

Chorus: Yes.

R: No.

I: During pregnancy?

Samantha: I would think that a vaginal ring is better.

I: I want us to first talk about the pill, we will also talk about the vaginal ring.

Sarah: I think I would take it because it would stop me from worrying about getting infected with HIV.

Annet: I would think it is not a good option for me because I do not like pills.

I: Why don’t you like pills?

Annet: Just thinking about it I vomit.

Pamela: It is not something I would like to use because I can forget to take it.

Aida: I do not have any problem with taking pills but my question is what if I forget to take it on that day.

I: If you forget to take it?

Aida: If you forget to take it on that day, what do you do?

Samantha: On my side, I would say no to that pill, after seeing the other woman’s experience who got a miscarriage when she started taking those pills I can’t take them.

Vanessa: It might not work for me because during pregnancy I vomit a lot and that makes me doubt whether it can stay inside when I vomit.

Shanitah: I think I would take it to stay safe in case my partner engages in sex with other women.

Angel: I also think I can use it.

**FGD Number:** FGD_41-F61

**Site:** Uganda

**Excerpt Range:** 59756-61104

I: But is it something you would have liked to use?

Chorus: Yes.

I: Why would you like to use it?

R: To prevent HIV.

Angel: It doesn’t worry like pills and it keeps it confidential because your partner cannot know that you have a vaginal ring or not.

Samantha: I think it is good because it doesn’t worry like tablets. And in a way you would be saved from the gossip of neighbours because if she entered your house and saw the tablets she will talk about it with the entire village. She will not think that you are taking it for prevention; she will think you are infected. But a vaginal ring keeps it confidential; no one will know that you have it.

Vanessa: It would work for me as an individual but it is too big.

Chorus: [Laughing].

I: How big is it?

Vanessa: It is big in size and it is hard.

I: Why are you scared of the size?

Vanessa: It is big and the man’s penis can just pass through it

Chorus: [Laughing].

I: Tell us.

Vanessa: You told us that we insert it ourselves but how can you insert such a big thing? That thing is hard and big.

R: Do you insert it using something or just the way she is holding it?

R: It is inserted the way she is holding it.

R: So, when it gets inside it unfolds?

R: Yes.

R: What if it fails to unfold?

R: Can’t that vaginal ring fail to unfold?

R: It can’t fail to unfold.

Chorus: [Speaking at the same time].

**FGD Number:** FGD_41-F61

**Site:** Uganda

**Excerpt Range:** 62590-63468

I: Is it acceptable for a woman to insert something in her vagina when she is pregnant traditionally?

Angel: It is not acceptable because it might affect the baby in the womb.

I: Is it a taboo?

Ritah: It depends, when a woman has a UTI there are some tablets she is given to insert in her vagina.

I: You are saying?

Ritah: That it is acceptable because when you are given UTI tablets you insert them.

I: Angel, you were saying that it is not acceptable?

Angel: Health workers do not advise us to insert anything in our vagina. That we shouldn’t disturb that area.

I: How about if it is the health workers who have given it to you and have recommended you to use it? Do our mothers, grandmothers and the rest accept that a woman should insert things in her vagina?

R: It is acceptable.

I: It is acceptable…

R: It is acceptable because you might find that it is required to do so.
