# Supplementary material for: Pregnant and breastfeeding women’s prospective acceptability of two biomedical HIV prevention approaches in Sub Saharan Africa: A multisite qualitative analysis using the Theoretical Framework of Acceptability
Source: PLoS One. 2021 Nov 16;16(11):e0259779. doi: 10.1371/journal.pone.0259779 (PMC8594804; doi:10.1371/journal.pone.0259779)
Supplement: Attachment — Submitted filename: Response to Reviewers.docx [file pone.0259779.s003.docx]

2**0^th^ July 2021**

Dear Dr Dubé,

**RE: Manuscript Number:** PONE-D-21-07808 Pregnant and breastfeeding women's prospective acceptability of two biomedical HIV prevention approaches in Sub Saharan Africa: a multisite qualitative analysis using the Theoretical Framework of Acceptability

Thank you for considering the first version of our manuscript. We would like to thank you and the reviewer for the helpful comments and suggestions on how to improve the manuscript. We have addressed each of the minor comments and fully describe these in table format below. We have attached a clean version of the updated manuscript as requested.

We hope the revisions above address comments. We look forward to hearing from you in due course.

Yours Sincerely,

Dr Mandeep Sekhon

Research Associate

School of Population Health & Environmental Sciences

Faculty of Life Sciences and Medicine

King’s College London

2^nd^ Floor, Addison House

Guy’s Campus

London, SE1 1UL

[Mandeep.sekhon@kcl.ac.uk](mailto:Mandeep.sekhon@kcl.ac.uk)

**Manuscript Number:** PONE-D-21-07808 Pregnant and breastfeeding women's prospective acceptability of two biomedical HIV prevention approaches in Sub Saharan Africa: a multisite qualitative analysis using the Theoretical Framework of Acceptability

**Table 1 : Reviewer comments, and how our revisions address these comments**

| **Comment/ suggestion for improvement** | **Author response and changes made** | **page number in revised m/s** |
| --- | --- | --- |
| **Editor** | | |
| 1. Please ensure that your manuscript meets PLOS ONE's style requirements, including those for file naming. | Thank you for providing links to the PLOS One templates. We have now revised our submission in line with the templates.  We also have updated our reference list so that it meets the journals requirements. | N/A |
| 2. Thank you for stating the following in the Competing Interests section:  "The authors have declared that no completing interests exist. "  We note that one or more of the authors are employed by a commercial company: ASTRA Consulting.  2.1. Please provide an amended Funding Statement declaring this commercial affiliation, as well as a statement regarding the Role of Funders in your study. If the funding organization did not play a role in the study design, data collection and analysis, decision to publish, or preparation of the manuscript and only provided financial support in the form of authors' salaries and/or research materials, please review your statements relating to the author contributions, and ensure you have specifically and accurately indicated the role(s) that these authors had in your study. You can update author roles in the Author Contributions section of the online submission form. | The commercial affiliation (ASTRA consulting) had no role in the study or the paper writing. ASTRA consulting is Dr. van der Straten’s current affiliation as an independent consultant to the MTN, but this change happened after the study and the writing of the manuscript was completed.  The funder provided support in the form of salaries for authors [MS and AVDS] but did not have any additional role in the study design, data collection and analysis, decision to publish, or preparation of the manuscript.  The specific roles of these authors are articulated in the ‘author contributions’ section.” | N/A |
| 2.2. Please also provide an updated Competing Interests Statement declaring this commercial affiliation along with any other relevant declarations relating to employment, consultancy, patents, products in development, or marketed products, etc | We added Competing Interests Statement that Dr van der Straten is currently an independent consultant and sole proprietor of “ ASTRA consulting”. This affiliation does not alter our adherence to  PLOS ONE policies on sharing data and materials.”  We have updated the Funding Statement and Competing Interests Statement in our manuscript. Thank you for the editors for changing the online submission form on our behalf. | 36 |
| 3. We noted in your submission details that a portion of your manuscript may have been presented or published elsewhere.  Please clarify whether this publication was peer-reviewed and formally published. If this work was previously peer-reviewed and published, in the cover letter please provide the reason that this work does not constitute dual publication and should be included in the current manuscript | The current study reported in this manuscript was embedded within the Microbicide Trial Network (MTN)- funded Microbicide/PrEP Acceptability among Mothers and Male Partners in Africa (MTN-041/MAMMA), a multi-site qualitative study to identify individual, interpersonal, social and cultural factors that may affect the uptake by pregnant and breast feeding women in Africa of the monthly dapivirine vaginal ring and daily oral pre-exposure prophylaxis (PrEP).  Primary objectives of the MAMMA study included exploring amongst pregnant and breastfeeding women, and men with pregnant or breast feeding partners (1) perceptions of HIV ; (2) attitudes about the use of the vaginal ring and oral PreP during pregnancy and breastfeeding; (3) willingness to use, recommend or support the vaginal ring or oral PrEP during pregnancy and breastfeeding in the future, and (4) prospective acceptability of the vaginal ring and oral PrEP amongst pregnant and breastfeeding women.  The first three objectives of MAMMA study are reported by van der Straten and colleagues 2020 (the primary publication which was peer reviewed):   - van der Straten, A., Ryan, J. H., Reddy, K., Etima, J., Taulo, F., Mutero, P., ... & MTN‐041/MAMMA Study Team. (2020). Influences on willingness to use vaginal or oral HIV PrEP during pregnancy and breastfeeding in Africa: the multisite MAMMA study. *Journal of the International AIDS Society*, *23*(6), e25536.   In the above paper, 16 single sex focus group discussions with HIV un-infected pregnant and breastfeeding women (n= 65) and men with partners who were currently or recently (within two years) pregnant or breastfeeding (n=63) were completed. A thematic analysis and a socio-ecological framework was applied to analyse all data, to identify the perceptions of risk, attitudes about the use of both PrEP products, and the willingness to use in the future, recommend or support the use of both PrEP products. Within our submitted manuscript, in the introduction, we summarise the findings from the above paper, and we highlight the key difference for the current manuscript submitted to PLOS one.  In the current manuscript, we report in detail findings for the fourth objective of the MAMMA study: prospective acceptability of the vaginal ring and oral PrEP amongst pregnant and breastfeeding women. We completed a secondary qualitative analysis, focusing specifically on the 65 pregnant and breastfeeding women (same female cohort as the van der Straten et al 2020 paper). The secondary analysis was guided, by the Theoretical Framework of Acceptability to explore pregnant and breastfeeding women’s prospective acceptability of the daily oral pills and the monthly vaginal ring, and to identify how the acceptability of both PrEP products can be enhanced.  In our manuscript the primary publication is cited, and it does not constitute dual publication. It leverages a portion of the primary data (for FGD with P/BF women) that were re-analyzed with the Theoretical framework of Acceptability (TFA) in this manuscript, but otherwise is complementary, not duplicative to the primary study paper. | N/A |
| 4. In your Data Availability statement, you have not specified where the minimal data set underlying the results described in your manuscript can be found. PLOS defines a study's minimal data set as the underlying data used to reach the conclusions drawn in the manuscript and any additional data required to replicate the reported study findings in their entirety. All PLOS journals require that the minimal data set be made fully available. For more information about our data policy, please see <http://journals.plos.org/plosone/s/data-availability> | The minimal data set underlying the results reported in this manuscript are now included as supplementary files. The data consists of five code reports produced from the primary thematic analysis (completed for the Van Straten et al, 2020 paper) . The code reports were produced from in Dedoose software (v7.23).  We have added a reference to the supplementary file within the main text:  Only five code reports were deemed relevant for the acceptability analysis of both PrEP methods (Acceptability, barriers ring, facilitators ring, barriers oral pill, facilitators oral pill) (Supplementary file 2). | 9 |
| **Reviewer 1** | | |
| Overall, the study was thoughtfully conceptualized, and it addresses an area in which there is very little research. HIV prevention during pregnancy and lactation is very important, as these are times of high HIV susceptibility; but PrEP is underutilized during these times in a woman’s life.  An additional strength of the study is that it explores acceptability as a multifactorial concept, including not only product attributes, but also user, partner and community factors. Applying the TFA construct in this realm is a methodological advance. Inclusion from women from four different countries increases generalizability of findings, even though the number of women from each site was relatively small.  The findings also highlighted the importance of personal beliefs and societal/cultural norms as determinants of product acceptability, particularly during pregnancy and breastfeeding, and suggested factors that are important to include in informational and counselling materials of these PrEP modalities when they are being implemented. | Thank you for taking the time in reviewing the manuscript. We are pleased that the study reported in the paper is considered thoughtfully conceptualised and has been acknowledged as an area with very little research.  Thank you for the positive feedback. | N/A |
| In Introduction: The statement “Primary findings indicate that there was consensus amongst men and women, that P/BF women are at higher risk of HIV due to their partners infertility.” What does that mean? Why are the partners of pregnant women infertile and why is this relevant to the women’s higher risk for HIV? Please clarify | Thank you, this was a typo in the manuscript. We have now changed this in the text, so this section reads as follows:  Primary findings indicate that there was consensus amongst men and women, that P/BF women are at higher risk of HIV due to their partners **infidelity**. | 4 |
| **Reviewer 2** | | |
| Thank you for the opportunity to review this manuscript which describes a qualitative exploration of the prospective acceptability of oral PrEP and a vaginal ring PrEP. The authors have clearly and rigorously performed a secondary analysis to tie PrEP usage themes to the TFA. This is a thorough analysis on a concept that has received little attention previously. | Thank you for taking the time in reviewing the manuscript. We are pleased that the reviewer considers our secondary analysis guided by the TFA as a rigorous and thorough approach. | N/A |
| I have only a few minor comments including potentially incorporating differences among the participants at different sites since the four countries will likely have differing norms and values in regards to pregnancy, breastfeeding, and HIV prevention. | Thank you for this suggestion. As the main aim of the primary study was not to complete a cross- country comparison we are unable to discuss the potential variability in cultural norms and practices and how these may influence perceptions of acceptability of PrEP and the vaginal ring. We have added the following sentence in the discussion to account for this:  The main limitation of this study was the small number of FGDs per participant per site, as we didn’t intend to do cross country comparisons at the outset. The study was intended for overall analysis in high HIV prevalence and incidence settings, and we found many similarities in responses across the study sites. Some site variations were previously noted, for example with respect to P/BF women’s differing views about the effects of taking bitter medications, or regarding the ring being possibly misperceived by providers as an abortion tool (7). Because the study was not designed to assess attitudes by geographical or cultural settings, more research would be needed to ascertain whether these relate or not to cultural norms | 30 |
| Introduction 1. Paragraph 2, Line 4, please spell out acronyms prior to use (fTC/TDF). | Thank you for spotting this we have now changed this. The sentence has now been changed:  The Word Health Organisation recommends offering daily Truvada™ or emtricitabine(FTC) / tenofovir disoproxil fumarate (TDF) pills to women at considerable risk of HIV infection (5). | 3 |
| 2. Paragraph 6, Line 2, use of study twice in the same sentence, “the Microbicide Trial Network (MTN)- funded a study multi-site qualitative study.” | Thank you for spotting this typo. The sentence has now been changed in the manuscript and reads as follows:  the Microbicide Trial Network (MTN)- funded a multi-site qualitative study.” | 3 |
| 3. Paragraph 6, Line 2, please explain the primary findings from reference 7, indicating that participants felt P/BF women were at higher risk of HIV due to partners’ infertility. Was infertility an inclusion criterion for study participation? | Thank you, this was a typo in the manuscript. We have now changed this in the text, so this section reads as follows:  Primary findings indicate that there was consensus amongst men and women, that P/BF women are at higher risk of HIV due to their partners **infidelity**. | 4 |
| 4. Final paragraph, Line 2, in the sentence, “In this paper, we apply the TFA to complete a secondary analysis of the eight FGDs completed with B/BF as part of the MAMMA study” B/BF is likely a typo and should be P/BF. | Thank you, this was a typo. We have now changed this in the text, and reads as follows:  In this paper, we apply the TFA to complete a secondary analysis of the eight FGDs completed with P/BF as part of the MAMMA study | 6 |
| Discussion 1. Paragraph 4, line 3-4, can the authors expand upon why it was difficult to code data on side effects and safety concerns? | Thank you for the suggestion. We have expanded on our explanation providing an example of women’s perceptions of side effects and safety concerns. The section now reads as follows:  However, it was challenging to code data that focused on women’s thoughts on the potential side effects, and safety concerns associated with the pills. Women’s perspectives on side effects centred around the potential for oral PrEP to cause side effects similar to the contraceptive pill. Concerns relating to the safety of oral PrEP were reflected as women’s misunderstanding of how that pill works. Women stated that taking the oral pill could lead to miscarriages or that the baby may be born with disabilities.  This issue highlights the importance of understanding the nuances of the TFA definitions. Through iterative discussions, data referring to the potential side effects anticipated with the pills was coded into the construct of burden, on the basis that each intervention is likely to have potential benefits and potential disadvantages. Here, perceptions that women may experience side effects (e.g. vomiting) were considered a high burden (i.e. high levels of effort) and a disadvantage of taking oral PrEP. Data referring to anticipated safety concerns with taking the pills was coded into the construct of intervention coherence (i.e. poor understanding of how oral PrEP works). | 28-29 |
| 2. Given that the interviews were conducted in four very different countries and settings, could the authors include some discussion of the variability in cultural norms and practices and their effect on the acceptability of PrEP and the vaginal ring? | Thank you for this suggestion. As the main aim of the primary study was not to complete a cross- country comparison we are unable to discuss the potential variability in cultural norms and practices and how these may influence perceptions of acceptability of PrEP and the vaginal ring. We have added the following sentence in the discussion to account for this:  The main limitation of this study was the small number of FGDs per participant per site, as we didn’t intend to do cross country comparisons at the outset. The study was intended for overall analysis in high HIV prevalence and incidence settings, and we found many similarities in responses across the study sites. Some site variations were previously noted, for example with respect to P/BF women’s differing views about the effects of taking bitter medications, or regarding the ring being possibly misperceived by providers as an abortion tool (7). Because the study was not designed to assess attitudes by geographical or cultural settings, more research would be needed to ascertain whether these relate or not to cultural norms | 30 |
